# Climatic refugia and reduced extinction correlate with underdispersion in mammals and birds in Africa

**DOI:** 10.1002/ece3.8752

**Published:** 2022-03-23

**Authors:** Jacob C. Cooper, Nicholas M. A. Crouch, Adam W. Ferguson, John M. Bates

**Affiliations:** ^1^ Committee on Evolutionary Biology University of Chicago Chicago Illinois USA; ^2^ Negaunee Integrative Research Center Field Museum Chicago Illinois USA; ^3^ Department of Geophysical Sciences University of Chicago Chicago Illinois USA; ^4^ Gantz Family Collections Center Field Museum Chicago Illinois USA; ^5^ Present address: University of Kansas Biodiversity Institute Lawrence Kansas USA

**Keywords:** biogeography, birds, community structure, macroecology, macroevolution, mammals, museums and cradles

## Abstract

Macroevolutionary patterns, often inferred from metrics of community relatedness, are often used to ascertain major evolutionary processes shaping communities. These patterns have been shown to be informative of biogeographic barriers, of habitat suitability and invasibility (especially with regard to environmental filtering), and of regions that function as evolutionary cradles (i.e., sources of diversification) or museums (i.e., regions of reduced extinction). Here, we analyzed continental datasets of mammal and bird distributions to identify primary drivers of community evolution on the African continent for mostly endothermic vertebrates. We find that underdispersion (i.e., relatively low phylogenetic diversity compared to species richness) closely correlates with specific ecoregions that have been identified as climatic refugia in the literature, regardless of whether these specific regions have been touted as cradles or museums. Using theoretical models of identical communities that differ only with respect to extinction rates, we find that even small suppressions of extinction rates can result in underdispersed communities, supporting the hypothesis that climatic stability can lead to underdispersion. We posit that large‐scale patterns of under‐ and overdispersion between regions of similar species richness are more reflective of a particular region’s extinction potential, and that the very nature of refugia can lead to underdispersion via the steady accumulation of species richness through diversification within the same ecoregion during climatic cycles. Thus, patterns of environmental filtering can be obfuscated by environments that coincide with biogeographic refugia, and considerations of regional biogeographic history are paramount for inferring macroevolutionary processes.

## Graphical abstract

We compared and contrasted macroevolutionary patterns between mammals and birds within the African continent. Using empirical and theoretical data and metrics such as phylogenetic dispersion, shown here, we found that community structure can be explained by relative extinction rate between habitats. Our study improves our understanding of African vertebrate community dynamics while highlighting the importance of climatic structure.

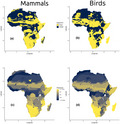

## 1 INTRODUCTION

**FIGURE 1:**
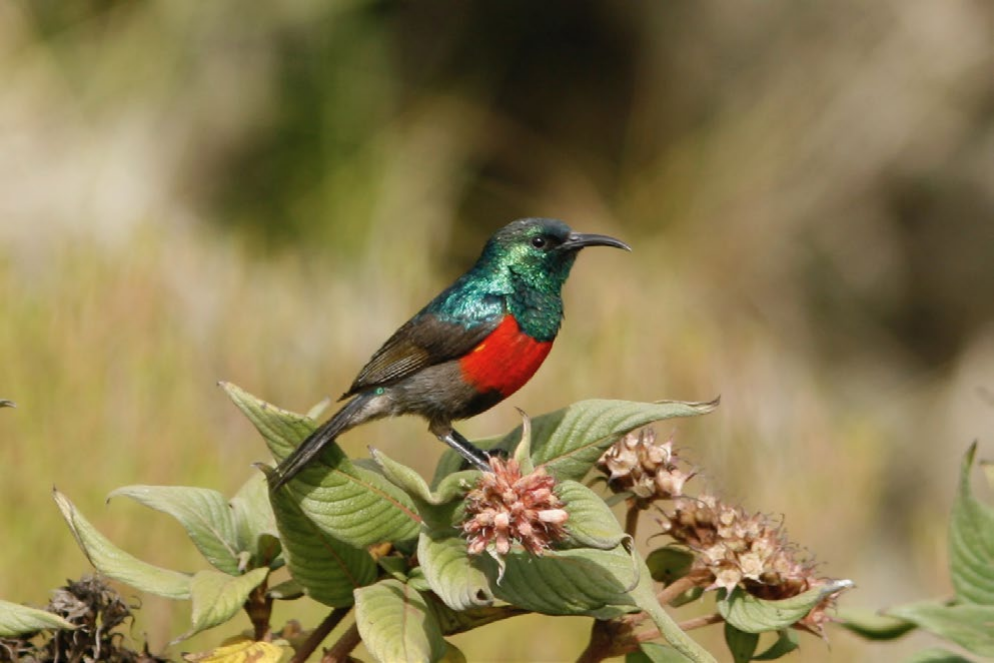
A Northern Double‐collared Sunbird *Cinnyris reichenowi preussi* on Pico Basilé, Equatorial Guinea. While the genus *Cinnyris* is well known for its montane diversity (including the species shown here), the complex is also diverse and widespread in the tropical lowlands, areas often considered “museums.” Photo by Dr. Oscar Johnson, ML269531451; used with permission

Quantitative assessments of community assembly and relatedness are often used to assess macroevolutionary patterns of diversification at large spatial scales (Crouch et al., 2019; Gerhold et al., 2015). On the African continent, range disjunctions shared at the community level have long been studied and interpreted from the perspective of how climate can shape biogeographic patterns (Fjeldså & Bowie, 2008; Hall & Moreau, 1970; Kingdon, 1989; Voelker, Outlaw, & Bowie, 2010; Vrba, 1993). In addition, spatial patterns of species richness and diversification across Africa have led to the identification of evolutionary “cradles” such as mountains that play a crucial role in building African biodiversity (Fjeldså & Bowie, 2008; Fjeldså et al., 2011). Similarly, lowland habitats, especially more stable, tropical environments, have been referred to as “evolutionary museums”—areas in which species diversification is comparatively older and tempered, meaning communities (and species assemblages themselves) have been relatively stable through time (Figure 1) (Azevedo et al., 2020; Gaston & Blackburn, 1996; Marks, 2010).

Whether on islands or across continents, local and regional species assemblages are created through a combination of diversification and colonization events, balanced by extinctions (Lomolino, 2016). Truly in situ diversification (i.e., wholly sympatric speciation) appears to be relatively rare in nature or requiring specific conditions, with parapatric and allopatric speciation being far more common (Coyne & Price, 2000; Smith, 1966). Allopatric speciation relies on geographic isolation of species, which only increases the overall species richness when taxa come into secondary contact and their species integrity is maintained (Tobias et al., 2020). This secondary contact results in the colonization of one or both species into an area that is ecologically suitable but hitherto inaccessible for one or both species. Over evolutionary time, the probability of secondary contact increases, such that on large timescales (i.e., hundreds of thousands or millions of years) increasingly distantly related taxa overlap. Across shorter evolutionary timescales, these divergences can result in evolutionary radiations (including geographic or adaptive radiations) of species that are closely related. These patterns can repeat as evolutionary time increases and a clade’s ecological niche expands. For example, multiple evolutionary radiations of different ecologies exist for Afrotherian mammals such as the Tenricidae (tenrecs) of Madagascar, with more recent radiations in some of the differentiated clades (Everson et al., 2016).

From a meta‐community perspective, an appearance of in situ diversification can be explained by diversification events that are limited to the same biome, with limited migration to other biomes (Marks, 2010; Nicolas et al., 2020). In situ diversification is thus driven primarily by temporary allopatry caused by climatic cycles (Prigogine, 1987; Vrba, 1993) or fluctuating geographic boundaries such as rivers (Crouch et al., 2019; Naka & Brumfield, 2018) or, to a lesser extent, by resource allopatry via ecological divergence among spatially overlapping populations (Benkman et al., 2009). These diversification processes create communities that are phylogenetically underdispersed (i.e., with low phylogenetic diversity relative to species richness) compared to other biomes that are more easily colonizable and thus more likely to possess less related lineages (Graham et al., 2009). This process could be accelerated by relatively frequent fragmentation of habitats by environmental cycling, where repeated opportunities for allopatry arise and are subsequently followed by the re‐establishment of sympatry or parapatry, thus causing underdispersion to co‐occur with temporally unstable environments with low community extinction rates (Vrba, 1993). In other biogeographic regions, underdispersion is often related to “filtering,” wherein only a few lineages are able to colonize and diversify within a specific environment (Jabot et al., 2008; Kraft et al., 2015). However, the root cause of the “filtering” has been called into question, and it is argued that environmental causes for filtering cannot be fully determined even if phylogenetic underdispersion is found to be associated with specific environments (Cadotte & Tucker, 2017).

Geographic characteristics affect different lineages in similar ways, such that diversity hotspots for multiple taxonomic groups are frequently spatially coincident (Hawkins et al., 2012; Lomolino et al., 2006). The ways in which climate and geography have influenced evolution (and continue to do so) are further dependent on the evolutionary histories of species and lineages. Like all continents, Africa is biogeographically complex, with northern Africa (namely, the Maghreb) being more closely allied to Eurasia, and with sub‐Saharan Africa is unique due to its greater biogeographic isolation (Cox, 2001; Husemann et al., 2014). Furthermore, sub‐Saharan Africa is associated with several island groups that vary dramatically in their biogeographic histories, with the largest island, Madagascar, possessing endemic incipient evolutionary radiations including Vangidae (vangas), Bernieridae (Tetrakas), Eupleridae (Malagasy carnivorans), Tenrecidae (tenrecs), and Lemuridae (lemurs) (Everson et al., 2016; Reddy et al., 2012; Yoder et al., 2003; Younger et al., 2018).

The biomes of sub‐Saharan Africa can be divided into biogeographic areas. Two major rainforests regions exist, the Upper Guinea forests and the Congo Rainforest (here considered with the connected Lower Guinea Forests), as well as several areas of varying aridity such as the Sahel and the Namib (one of the oldest deserts in the world), and several clusters of geographically disparate but historically connected montane habitats (Kingdon, 1989). Diversity for vertebrate groups peaks in the rugged highlands of the Lacustrine Rift (alternatively known as the Albertine Rift) (Cooper, 2021; Plumptre et al., 2007) where the Congo Rainforest and more xeric East African habitats intersect with some of the highest mountains on the continent. Unlike the Andes, which are a highly cohesive mountain chain bisected by deep river valleys and stretching over the breadth of the South American continent, the mountains of Africa primarily consist of scattered clusters of highlands, with the largest geological phenomenon being the Rift Valley. This region, stretching from Malawi and Mozambique to the Red Sea, has resulted in a discontinuous mix of mountains, depressions, and lakes that form an environmental mosaic lacking the uplifted continuity of the Andes or the Himalaya. While it is difficult to assess stability comparatively on continental regions through time, these differences in mountain building across continents may greatly influence comparative climates across continents. During the ice age, the lowland rainforests of Africa were spatially fragmented and restricted in distribution multiple times, creating fractured habitats that were archipelagic in their own right between periods of connectivity (Maley, 1996; Voelker, Outlaw, & Bowie, 2010). Thus, compared to these fracturing and reconnecting humid lowlands and highlands, many of the xeric and arid habitats of Africa have maintained relative stability, and are known for their remarkable biodiversity (especially for large mammals, Alaudidae [larks], and *Cisticola* [cisticolas; Cisticolidae]) (Alström et al., 2013; Davies, 2014; Kingdon, 2015).

The generation of broad phylogenetic datasets, coupled with documented distribution data in mammals and birds, offers the potential for continent‐wide comparisons that can distill the processes that skew phylogenetic dispersion in different habitats and different lineages. The archipelagic aspects of many African habitats through evolutionary time present an opportunity to examine the effects of environmental history and geography on patterns of richness and phylogenetic diversity. Specifically, the mix of mountains and lowlands provides an opportunity to determine whether similar biogeographic histories have led wet montane forest and wet lowland rainforest regions to possess similar patterns of community assembly. The geographically large regional environmental fluctuations and disjunctions are well documented, with evidence for a historically savanna‐like Sahara extending well into northern Africa (Skonieczny et al., 2019; Tierney et al., 2017) and it is possible that there have been repeated disjunctions and reconnections between different lowland rainforest refugia (Maley, 1996; Vrba, 1993).

To this end, we coupled a continent‐scale presence–absence matrix of mammals and birds in Africa with their respective global phylogenies. We performed continental comparisons of richness, mean phylogenetic distance (MPD), mean nearest taxon distance (MNTD), and phylogenetic dispersion (PD) for both mammals and birds, Africa’s most well‐known vertebrate groups. We supplement these analyses with a theoretical test of species diversification in similar sized communities with different extinction rates to compare how biogeographic refugia and stability may shape modern communities.

## 2 METHODS

### 2.1 General programs

All computational analyses were performed in **R** 4.0.4 locally or with **R** 3.6.3 on a server (R Core Team, 2021), using the package *tidyverse* and its dependencies for general data manipulation (Wickham et al., 2019) and the packages *colourvalues* (Cooley, 2020), *ggpubr* (Kassambara, 2020), *gridExtra* (Auguie, 2017), *rasterVis* (Perpiñán & Hijmans, 2021), and *viridis* (Garnier, 2018) to assist with plot creation. We performed spatial manipulations of the data using the **R** packages *raster* (Hijmans, 2020) and *sf* (Pebesma, 2018) as well as in **QGIS** 3.18 & 3.20 (QGIS Development Team, 2021). Image manipulation further used the GNU Image Manipulation Program (Kimball et al., 2020), ImageMagick (ImageMagick Studio, 2019), latex2rtf (http://latex2rtf.sourceforge.net/), and Inkscape (Inkscape Project, 2021).

### 2.2 Datasets

We processed mammals and birds independently, managed by JCC and NMAC, respectively. We downloaded global range maps for mammals from the International Union for the Conservation of Nature (IUCN, 2020) and global range maps for birds from BirdLife International and NatureServe (2012). We generated a presence–absence matrix for both groups using a grid of points that covered Africa and its adjacent offshore islands, sampling at .1° intervals using the *sp* function “over” in **R** (Bivand et al., 2013; Pebesma & Bivand, 2005). Subsequently, we downloaded taxonomic trees for mammals and birds from existing studies of global diversity. For mammals, we downloaded a phylogenetic dataset of all African taxa from <http://vertlife.org/phylosubsets/> with 100 trees (Upham et al., 2019). For birds, we used the genetic backbone of the Jetz tree (Jetz et al., 2012), placing species that lacked genetic information using Taxonomy Addition for Complete Trees (TACT) (Chang et al., 2020) and the taxonomic data of Jetz et al. (2012). This more recent approach for placing species using taxonomic data enables more accurate branch length estimation (Chang et al., 2020). We downloaded ecoregion information from the World Wildlife Federation website on 5 March 2021 (Olson et al., 2001).

### 2.3 Community analyses

We imported our presence–absence matrices into **R**, and calculated α diversity (i.e., richness) as the number of species reported present at each sampling point. For our analyses of MPD, MNTD, and phylogenetic dispersion, we used code from Crouch et al., (2018, 2019) supplemented with our own code. We imported phylogenetic trees into **R** using the package *ape* (Paradis & Schliep, 2019). We obtained values for MPD, MNTD, and phylogenetic diversity (PD) using the commands “mpd.query,” “mntd.query,” and “pd.query” in the **R** package *PhyloMeasures* (Tsirogiannis & Sandel, 2017) applied across our presence–absence matrix and all sampled phylogenetic trees. From these outputs, we obtained average values for each metric from each sampling point from our aforementioned grid, as well as the range of values obtained at each point. We calculated relative dispersion by performing a linear regression using the **R** command “lm” of mean phylogenetic diversity (from the “pd.query”) and species richness (Chambers, 1992; R Core Team, 2021; Wilkinson & Rogers, 1973). We then fit a 95% confidence interval around the regression line to define over‐ or underdispersion. We assigned a value of 0 to points that are underdispersed, 1 to points that are within the confidence interval, and 2 to points that are overdispersed. We compared the counts of points that fell into each of these categories for mammals and birds using a test called using the **R** function “chisq.test” (R Core Team, 2021).

Spatial patterns between the MPD, MNTD, and phylogenetic diversity of birds and mammals were compared using several methods. First, we performed simple linear models of the relationships between each descriptive variable (using “lm” in **R**) (Chambers, 1992; R Core Team, 2021) and we obtained the correlation between variables (using “cor” in **R**) (Becker et al., 1988; Kendall, 1938, 1945; R Core Team, 2021). To compare the landscapes of each variable, we converted our sampling points to raster format using the **R** package *raster* (Hijmans, 2020) and we normalized each variable between 0 and 1 using code adapted for our dataset (Appendix S1) (Cooper et al., 2022). We compared rasters using two commonly used metrics of niche overlap (Warren et al., 2008), Schoener’s *D* statistic and the similarly performing *I* statistic based on the Hellinger distance. These were calculated using the command “nicheOverlap” in the **R** package *dismo* (Hijmans et al., 2020), and we assessed significance by comparing the values of the real datasets to comparisons of the randomized matrices. Differences between the statistics obtained from actual datasets and the distribution of random datasets were obtained using *t*‐tests in **R** using the command “t.test” (R Core Team, 2021).

We imported the WWF ecoregions shapefile into **R** using the packages *rgdal* (Bivand et al., 2021), *rgeos* (Bivand & Rundel, 2020), *sp* (Bivand et al., 2013; Pebesma & Bivand, 2005), and *maptools* (Bivand & Lewin‐Koh, 2021) to compare values for each group across ecoregions, G200 regions (i.e., meta‐regions of multiple ecoregions), and user‐defined meta‐regions based on ecoregion type (e.g., forest, savanna, etc.). Given that African mammals and birds have different diversification histories, we focused on comparing relative amounts of phylogenetic dispersion between ecoregions. To obtain trends specific to the two classes, we assigned values of 0 for underdispersed, 1 for no significant dispersion, and 2 for overdispersed to each grid cell. We then averaged these values by ecoregion to assess spatially the distribution of over‐ and underdispersed ecoregions for both classes. We further combined mammals and birds datasets to determine which areas were relatively the most over‐ and underdispersed for both phylogenetic groups.

### 2.4 Theoretical analyses

To test for the effects of extinction alone on MPD and MNTD, we performed analyses of theoretical communities that are identical in every regard except extinction rate. These theoretical communities all originate from a single source species and then diversify to a predetermined richness, either 100 species or 10 species to mimic different levels of diversity observed in this study. In addition to the aforementioned **R** packages, we used *TreeSim* (Stadler, 2019) to create our theoretical communities. We created a custom function that would create two communities of equal richness with different extinction regimes and return a single dataframe with the community MPD and MNTD recorded (Appendix S1) (Cooper et al., 2022). For both communities, we defined three time periods, each with *λ* = 1.75 and *μ* = 0.1. We defined survival probability (*x*) manually for unstable (i.e., lacking refugium) communities, and defined survival rates for stable (i.e., possessing refugia) communities manually or according to equation (1) to create a semblance of proportionality.

(1) x.stable=0.9+x.unstable∗0.10

We performed multiple iterations of these tests, and varied them from having highly disparate levels of survival (e.g., 0.2 for unstable environments and 0.92 for stable environments; see Appendix S1:5) to similar rates of moderate survival (0.7 and 0.75) and similar rates of low survival (0.3 and 0.35) (Cooper et al., 2022). We ran 200 simulations for each scenario to get a sample distribution for comparisons between extinction rates. Distributions of MPD and MNTD were then compared with *t*‐tests using the **R** function “t.test” (R Core Team, 2021) and inspected visually using histograms.

## 3 RESULTS

### 3.1 Richness

**FIGURE 2:**
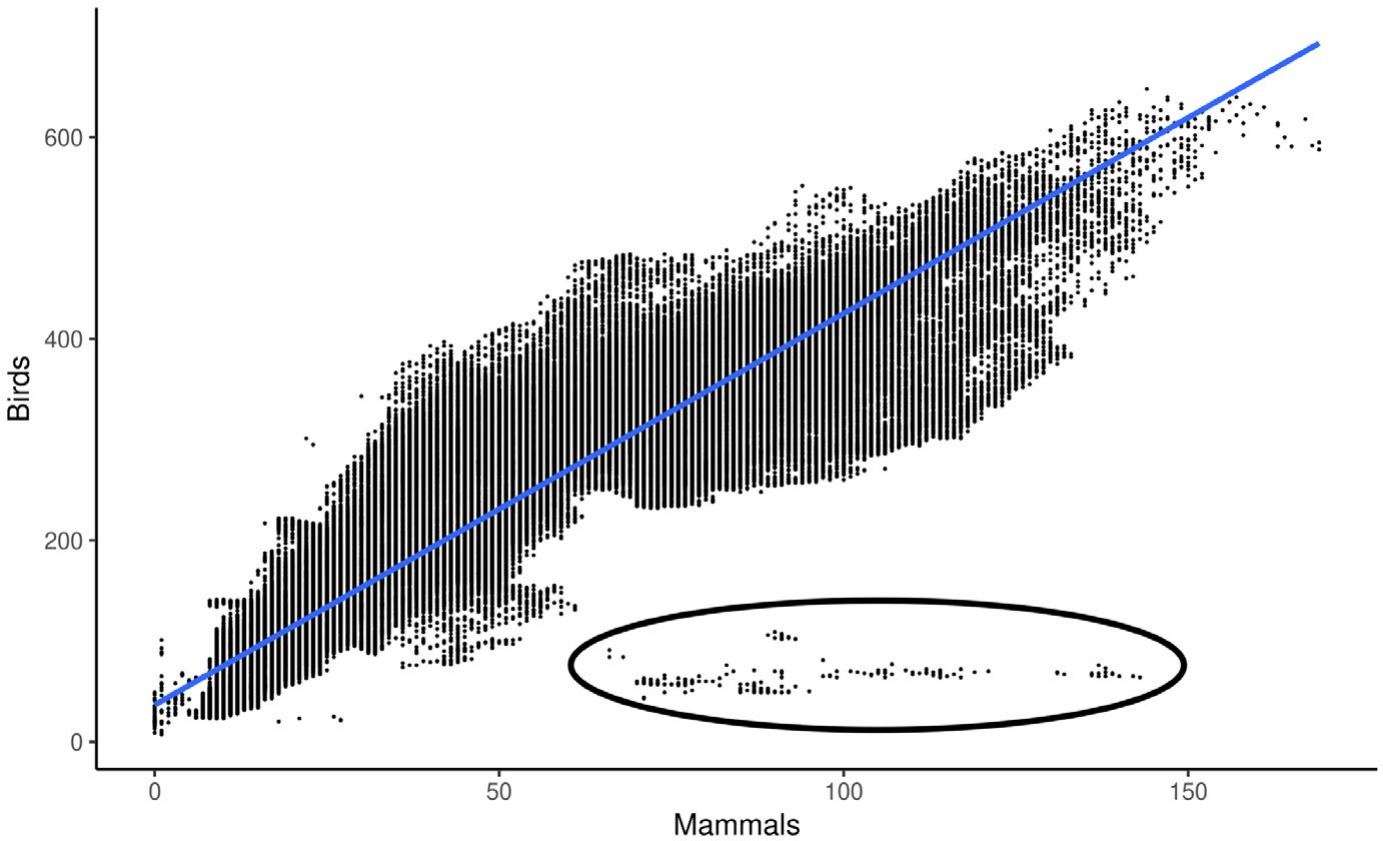
Linear regression between bird and mammal species richness across Africa. Note that the outliers (circled) denote areas close to lakes, where apparent spatial errors in mapping exist. The relationship between these mammals (m) and birds (b) can be approximated by the following equation (with 95% confidence intervals noted, and all variables supported by *p* < .05 and adjusted *R* ^2^ = .85): *m* = 3.89*b* + 36.58

**FIGURE 3:**
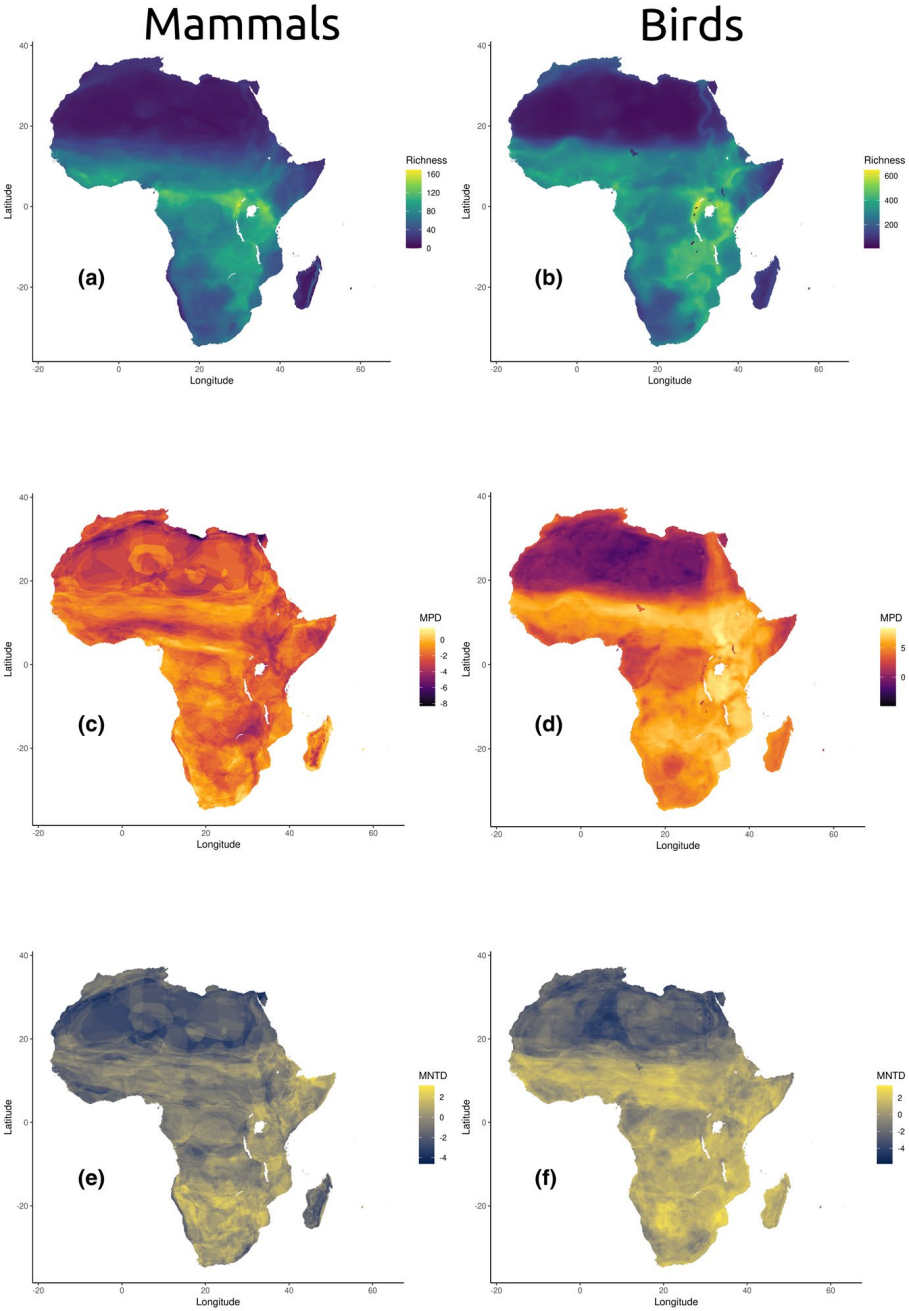
Diversity metrics for mammals and birds in Africa. a–b: Cumulative species richness (α diversity); c–d: Mean Phylogenetic Distance (MPD); e–f: Mean Nearest Taxon Distance (MNTD)

Our datasets included 1,305 species of mammals and 2,251 species of birds. We found that species richness of birds and mammals are positively correlated spatially (*p* < .05, adj. *R* ^2^ = .85, correlation = .92; *D* = .85, *p* < .05; Figure 2). Richness for both groups was highest in the topographically complex regions of Eastern Africa, especially in the Lacustrine Rift and in the highlands from Ethiopia south to Eswatini, Lesotho, and South Africa (Figure 3). Both groups also show signatures of high richness across the northern edge of the Congo Basin into Cameroon. These patterns are more easily observed in birds, almost certainly because of their higher species richness on the continent. A cluster of outlier points (*n* = 202, 0.0008% of total) was identified with high mammalian species richness and low avian species richness (i.e., more than 60 mammal taxa and fewer than 125 bird taxa). These areas are almost entirely from the fringes of large African lakes.

### 3.2 Mean phylogenetic distance

**FIGURE 4:**
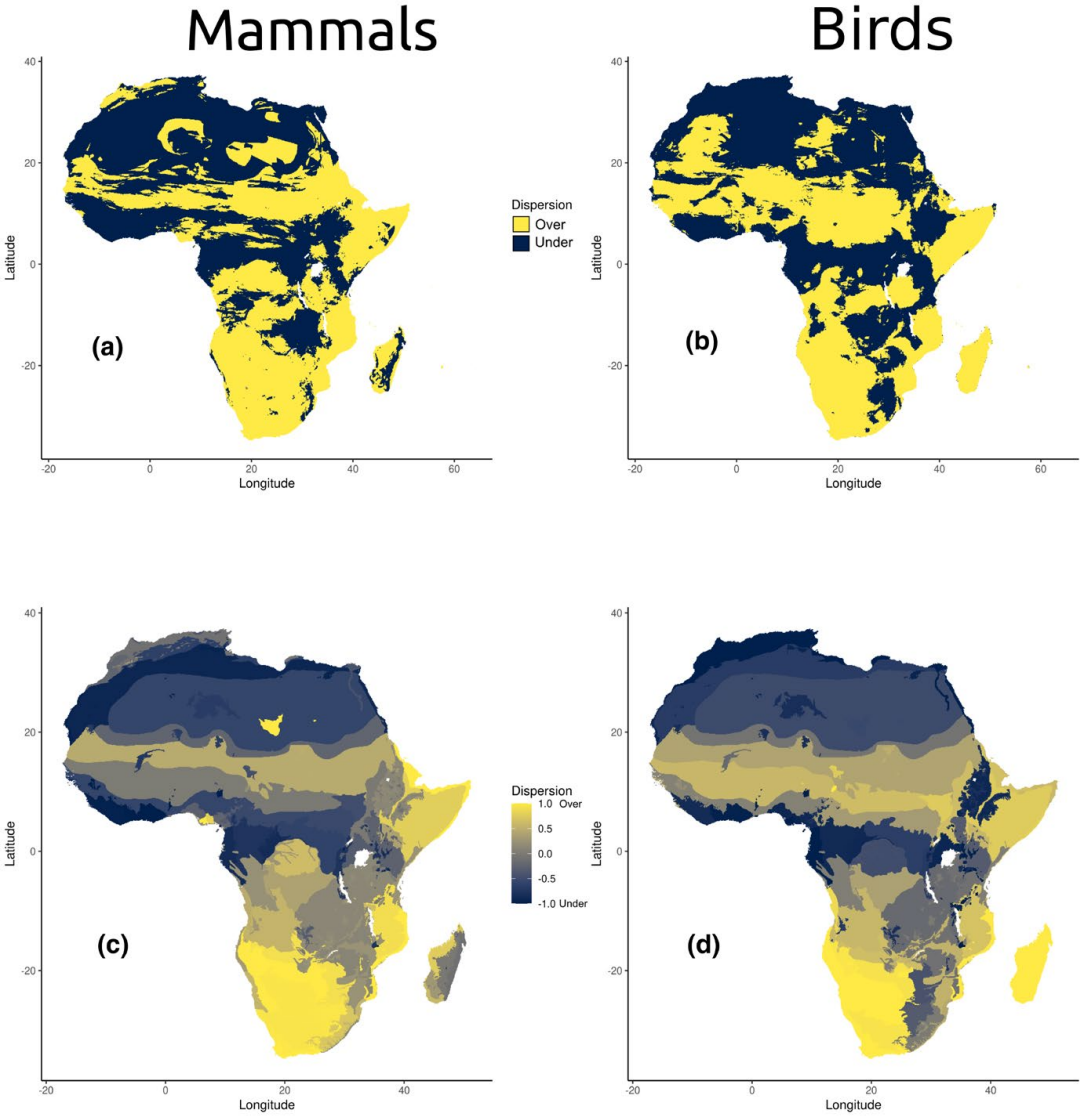
Dispersion in mammals and birds shown for individual grid cells (a–b, classified as underdispersed or overdispersed) and for values averaged within ecoregions (c–d; corrected from text to −1 for most underdispersed, 1 most overdispersed). Regional patterns that appear in the individual grid cells are accentuated within ecoregions, most notably the consistent phylogenetic underdispersion in equatorial rainforests, the Maghreb, and within the highlands of Eastern Africa. Note that some regional patterns differ between the two groups, especially within Madagascar, but the continental trends are similar

Mean phylogenetic distance differed in spatial patterning between the taxonomic groups, and, while a positive relationship between bird and mammal MPD exists, the correlation is relatively weak (*p* < .05, adj. *R* ^2^ = .13, correlation = .35; *D* = .91, *p* < .05). Birds exhibited a clear pattern of lower MPD in harsh environments (e.g., the Sahara) and higher MPD in species‐rich and mesic habitats (e.g., the Sahel), where communities are more diverse and many phylogenetically basal species (i.e., Common Ostrich *Struthio camelus*) reside (Figure 4a). Mammals had less clearly defined zones of low MPD, with parts of the Sahara, the African Rift, and transitions between the Sahel and the humid equatorial forests possessing relatively low MPD. Higher mammal MPD values predominated in the Sahel, the Congo, and more mesic habitats of southern Africa (Figure 4b).

### 3.3 Mean nearest taxon distance

Mean nearest taxon distance was found to be more correlated between birds and mammals than mean phylogenetic distance (*p* < .05, adj. *R* ^2^ = .48, correlation = .69; *D* = .90, *p* < .05). Both groups showed lower MNTD values in the Sahara and Namib deserts, and both groups also showed a signal of relatively lower MNTD in wet equatorial forests, specifically the Upper Guinea Forests, Lower Guinea Forests, Congo Rainforest, and Malagasy Rainforests. Relatively lower values also were shared in some highland regions, including the Ethiopian Highlands and the Great Escarpment of Southern Africa.

### 3.4 Phylogenetic dispersion

Direct comparisons of dispersion between birds and mammals found a weak but positive correlation between these two groups (*p* < .05, adj. *R* ^2^ = .09, correlation = .31; *D* = .94, *p* < .05). After transforming data into categorical categories of dispersion, patterns were found to be significantly more similar than random (*χ* ^2^ = 23,717, *df* = 4, *p* < .05). Specifically, both groups share underdispersion in xeric habitats (most notably, the Sahara) and in many wet equatorial habitats (specifically, Africa’s large tropical rainforests). Other regions with shared underdispersion include the higher elevations of Ethiopia, interior Southern Africa, and the East African Rift. Some areas of discordance in pattern between these groups include the Malagasy Rainforests (underdispersed in mammals, overdispersed in birds) and Atlas Mountains (overdispersed in mammals, underdispersed in birds).

When distilling patterns down to the level of ecoregion, we found clear associations between underdispersed communities and the Central African rainforests, the highlands of Eastern Africa, and parts of the Sahara and Maghreb. Some of the most notable regional differences between the two groups are lower phylogenetic dispersion in Madagascar for mammals (especially in the humid eastern part of the island) and lower phylogenetic dispersion for birds in Ethiopia and more broadly within the Southern African highlands (Figure 4).

### 3.5 Theoretical communities

**FIGURE 5:**
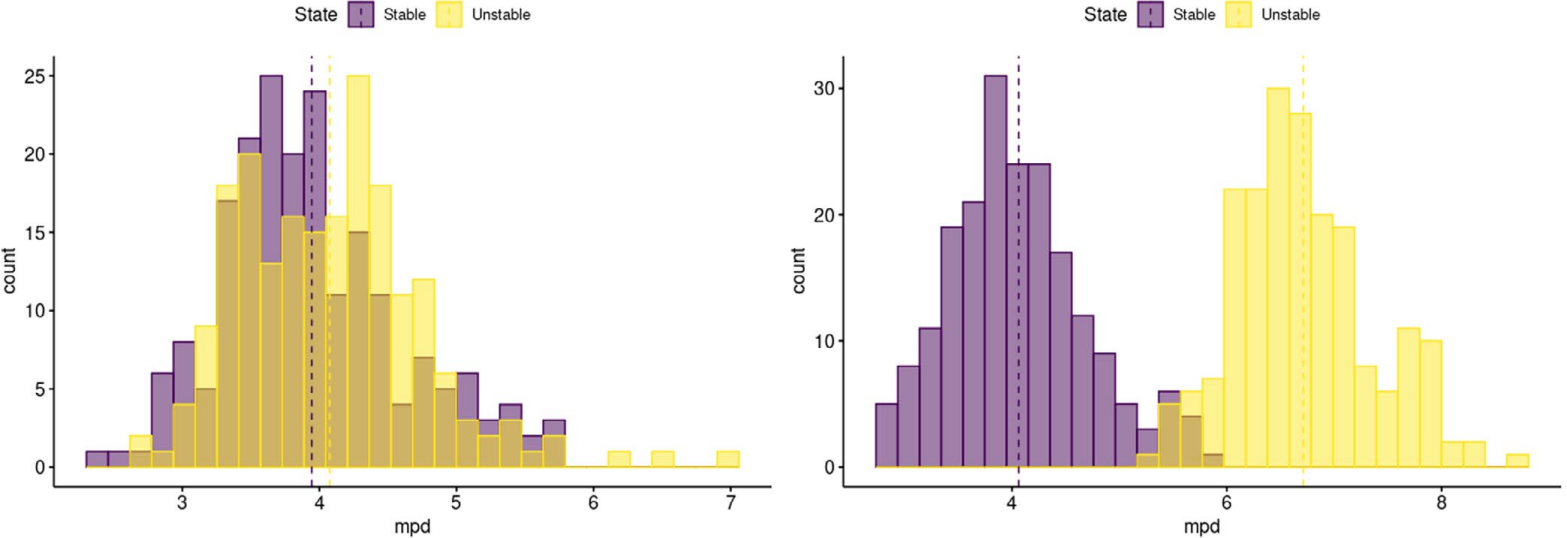
Comparisons of mean phylogenetic distance with varying extinction rates between communities with low (10%/1%) extinction rates for suitable habitats communities (i.e., refugia; left) and higher (70%/7%) extinction for habitats more greatly affected by perturbations (i.e., areas away from refugia; right). Differences between distributions are significant or near significant in both cases (*t*‐test; left: *t* = −1.96, *df* = 397.89, *p* = .05; right: *t* = −41.52, *df* = 397.99, *p* < .005). Plots are from Appendices S1:5.2.1 and S1:5.2.2, respectively (Cooper et al., 2022)

We found that, in every test performed, the average MPD and MNTD for “unstable” communities (i.e., those with higher extinction rates) exceeded those for “stable” communities (i.e., with lower extinction rates). These values were consistent, despite (sometimes broad) overlaps in the distribution of data derived from each scenario. We only observed an inability to separate these values when species diversity was low (i.e., 10) or extinction was low for both communities. Increasing the difference in extinction rates between the two communities and inflating the number of species present in a community exaggerated the disparity between MPD and MNTD distributions between the communities (Figure 5, Appendix S1:5) (Cooper et al., 2022).

## 4 DISCUSSION

African mammals and birds have important differences in their evolutionary histories, but they show strong geographic correlations across Africa with respect to their community assembly, with regions associated with climatic “refugia” possessing underdispersed communities and less climatically stable regions being more overdispersed. These areas include many regions often touted as refugia in sub‐Saharan Africa, such as the equatorial rainforests and mountains in the east, as well as the Maghreb, an area that functioned as a refugium for palearctic taxa (Griswold & Baker, 2002; Habel et al., 2011). Our theoretical models confirm that reduced extinction is sufficient for creating underdispersion in communities.

Previous studies on Afrotropical rainforest biodiversity have refuted the idea of evolutionary museums, pointing out large amounts of genetic diversity accumulated within continent‐spanning taxa, especially those within lowland humid forests (Huntley & Voelker, 2016; Marks, 2010). These patterns of diversification inherently point to the existence of multiple lowland rainforest refugia within the continent during past glacial cycles (Voelker, Outlaw, & Bowie, 2010). There is no doubt that these cycles have built diversity within this region, as evidenced by locally endemic species, but large portions of lowland rainforest assemblies are part of the same large radiations within similar habitats. A prime example of this includes Pycnonotidae (bulbuls) found throughout the wet equatorial African forests (Shakya & Sheldon, 2017). Montane areas—considered “cradles” of evolution due to their allopatric habitats and refugia during climate cycles (Fjeldså et al., 2011)—possess similar dynamics, and research elsewhere has revealed spatial coincidence between regions that would qualify as both “cradles” and “museums” (Azevedo et al., 2020). Our findings further the idea that refugia create museums in the sense that lineages persist and relative MPD and MNTD are decreased, but we show that this effect does not negate these regions’ ability to accumulate diversity, and highlights the need to focus on the processes building diversity beyond the scope of “museums” or “cradles” (Vasconcelos et al., 2021).

Our results are conditional on the accuracy of the spatial and taxonomic data for both groups. Many Afromontane and Afrotropical lowland taxa demonstrate surprising phylogeographic patterns upon close examination, yet many multispecies studies do not incorporate all described populations to determine if existing taxonomic assessments are correct (Cooper et al., 2021; Vaz da Silva, 2015; Voelker, Outlaw, & Bowie, 2010; Voelker, Outlaw, Reddy, et al., 2010). Taxonomic conservatism, a phenomenon that predominates in birds more so than mammals, is also potentially introducing bias within these studies (Watson, 2005). More thorough reviews of phylogeography and species limits are necessary to fully understand continental dynamics of diversification, although these taxonomic revisions may merely clarify which regions are “contact zones” between refugia rather than change the patterns observed within individual communities (Crouch et al., 2019). There are documented issues with IUCN range maps (such as those used here) (Herkt et al., 2017), particularly in tropical regions (Ficetola et al., 2014). We find many areas with high mammal richness and low bird richness derived from these maps are along the shorelines of lakes, suggesting spatial inconsistencies in how range maps are constructed for the two groups. While such patterns may represent a real signal in some localities, we believe that this lacustrine signal is also attributable to spatial errors in regions with complex topography (i.e., the presence or the absence of islands, steep elevation clines, etc.), especially given its prevalence across a large latitudinal range and across a variety of lake sizes, from the shores of Lake Victoria and reservoirs along the Zambezi (Appendix S1.2.2.1) (Cooper et al., 2022). The coarse detail of the taxonomic and spatial sampling of this study likely minimizes potential effects in estimated community structure regarding the inaccuracy of range margins.

Low MPD and MNTD are often inferred to be the result of environmental filtering, with the Andes being upheld as a particular example of “niche expansion” and diversification within the limited lineages that colonized the region (Graham et al., 2009). However, other factors (such as competition) also have been put forward as alternative causes to the same patterns as filtering (Cadotte & Tucker, 2017). At the level of Mammalia and Aves in Africa, we demonstrate that reduced rates of extinction can also lead to these similar patterns. Thus, patterns of filtering (i.e., underdispersion) can be obfuscated by environments that coincide with biogeographic refugia. It is, therefore, important to consider the biogeographic history of the clade and species being studied when determining the cause of underdispersion patterns. Underdispersion within specific environments across multiple families and classes of organisms is linked to climatic stability and the persistence of lineages that have diversified in situ within the greater metacommunity during disjunctions in spatially discrete refugia. Environmental filtering may be a cause of reduced MPD and MNTD for certain regions and certain lineages (such as within Afromontane sunbirds of the genus *Cinnyris*; Figure 1) (Bowie, 2003), but our findings indicate environmental filtering may also be an illusion of specific environments having reduced extinction while maintaining high levels of diversification. Thus, despite Afromontane regions being considered hotspots for diversification (Fjeldså & Bowie, 2008; Fjeldså et al., 2011; Rahbek et al., 2019), similar patterns of diversification will also occur in other geographic regions when environmental cycling creates similar patterns of habitat fracturing with stable refugia, as demonstrated by the Afrotropical rainforests (Huntley et al., 2019; Huntley & Voelker, 2016; Marks, 2010; Voelker et al., 2017), savannas of southern and eastern Africa (Aghová et al., 2017, p. 20; McDonough et al., 2015), and the Maghreb (Griswold & Baker, 2002).

Regional differences in phylogenetic dispersion illuminate how climatic cycles have shaped the bird and mammal diversity of the African continent. The disparities observed between mammals and birds highlight the differential short‐term responses of these communities to climate change while also highlighting how shared refugia result in similar patterns for both groups through evolutionary time (Riddell et al., 2021). Both birds and mammals show high richness, high MPD, and high MNTD in Eastern and Southern Africa. These areas are home to some of the most topographically complex regions within Africa, including the highest (Kilimanjaro, 5,892 m asl) and lowest (Lac Assal, −153 m asl) elevations on the entirety of the African continent (Wikipedia, 2021). In addition to hosting large amounts of habitat variability, the African Rift region is relatively centrally located within the continent (and thus relatively central for species dispersing across the continent) (Cooper, 2021). This variety has resulted in the persistence of some phylogenetic outliers, such as the Tanzanian partridges (genus *Xenoperdix*) (Dinesen et al., 1994; Fjeldså et al., 2011), but the overall pattern of underdispersion is still maintained. Notably, while locally endemic species are often associated with montane habitats, the xeric lowlands of East Africa are also home to restricted range species, such as Cosens’s Gerbil *Gerbillus cosensis* (Gerrie & Kennerley, 2016) and Beesley’s Lark *Chersomanes beesleyi* (Sinclair & Ryan, 2010).

Madagascar shows some of the clearest disparities between mammals and birds within our study, perhaps due to the nature of colonization frequency and pattern by birds and mammals (Everson et al., 2016; Reddy et al., 2012) coincident with the museum‐like nature of islands for taxonomic groups that are no longer found in continental settings (Kirchman et al., 2001; McCullough et al., 2019). Notably, the insular nature of Madagascar makes it theoretically more accessible to birds than to terrestrial mammals. Indeed, for birds, we see island wide overdispersion, undoubtedly related to the co‐occurrence of “recent” colonists radiations with more “ancient” lineages found throughout the island, such as Brachypteraciidae (ground rollers) and Mesitornithidae (mesites). Mammals, however, are dominated by infrequent colonizations that can be quite old, with contemporary climate patterns working against colonization (Stankiewicz et al., 2006). In this group, we see a repeat of the same patterns as mainland Africa. Wetter eastern rainforests, areas that have been identified as climatic refugia (Rakotoarinivo et al., 2013; Raxworthy & Nussbaum, 1995), possess underdispersed communities relative to the xeric western regions.

We found phylogenetic patterns were more highly structured for birds than for mammals. This is driven in part by distributions and taxonomy for birds being more well known, with many African mammal complexes (specifically shrews, rodents, and bats) still possessing large amounts of taxonomic flux (Hutterer et al., 2019; Monadjem et al., 2021; Nicolas et al., 2020). It is also possibly attributable to differential responses to climate change between the two groups on shorter timescales (Riddell et al., 2021). Nevertheless, we recovered positive correlations between the MPD and MNTD of both groups, and the spatial distribution of over‐ and underdispersed regions overlap extensively. Overdispersed communities for both groups are concentrated in xeric and semi‐arid habitats across the continent (with the notable exception of the Sahara), with underdispersed communities being concentrated in low‐elevation rain forest and high‐elevation habitats across the continent.

## 5 CONCLUSIONS

Underdispersion has often been associated with environmental filtering or with limited dispersal within or between specific environments. An overlooked consideration is that stability, in the form of refugia, and depressed extinction rates may result in these same patterns, and that both montane and lowland regions can exhibit similar patterns when both possess similar patterns of stability. In Africa, underdispersion in mammals and birds is clearly linked to climatic refugia, suggesting that underdispersion at large taxonomic scales can be caused by relative extinction rates.

## CONFLICT OF INTEREST

The authors declare no conflict of interest.

## AUTHOR CONTRIBUTIONS

**Jacob C. Cooper:** Conceptualization (equal); Data curation (equal); Formal analysis (lead); Methodology (equal); Visualization (equal); Writing – original draft (lead); Writing – review & editing (lead). **Nicholas M. A. Crouch:** Conceptualization (equal); Data curation (equal); Formal analysis (supporting); Methodology (equal); Writing – original draft (supporting); Writing – review & editing (supporting). **Adam W. Ferguson:** Writing – original draft (supporting); Writing – review & editing (supporting). **John M. Bates:** Conceptualization (equal); Supervision (lead); Writing – original draft (supporting); Writing – review & editing (supporting).

## Supporting information

Appendix S1

## Data Availability

Codes for this study are available in Appendix S1, available on Dryad (Cooper et al., 2022) and on GitHub. Additional codes referenced in this study are also available through Dryad (Crouch et al., 2018).
