# Supplementary material for: Climatic refugia and reduced extinction correlate with underdispersion in mammals and birds in Africa
Source: Ecol Evol. 2022 Mar 23;12(3):e8752. doi: 10.1002/ece3.8752 (PMC8941498; doi:10.1002/ece3.8752)
Supplement: Supplementary file 1 — Appendix S1 [file ECE3-12-e8752-s001.pdf]

Appendix 1

Cooper, Crouch, Ferguson & Bates

27 September 2021

1 Introduction

This is the supplementary material for the manuscript by JC Cooper, NMA Crouch, A Ferguson, and JM Bates. This document is presented *as is* and will need to be tailored for use on different machine and for different datasets. Much of this was run on `R 3.6.3` on a Linux server at UChicago. Final analyses were run locally in `R 4.0.4` on a Linux operating system.

NOTE: Some of these analyses run *every time* this document is generated, and thus values may not be an exact match to the publication, but patterns will.

The following packages were used throughout the pipeline:

```
library(ape)
library(colourvalues)
library(dismo)
```

```
## Loading required package: raster
```

```
## Loading required package: sp
```

```
##
## Attaching package: 'raster'
```

```
## The following objects are masked from 'package:ape':
##
##     rotate, zoom
```

```
library(ggpubr)
```

```
## Loading required package: ggplot2
```

```
##
## Attaching package: 'ggpubr'
```

```
## The following object is masked from 'package:raster':
##
##     rotate
```

```
## The following object is masked from 'package:colourvalues':
##
##     get_palette
```

```
## The following object is masked from 'package:ape':
##
##     rotate
```

```
library(gridExtra)
library(maptools)
```

```
## Checking rgeos availability: TRUE
## Please note that 'maptools' will be retired by the end of 2023,
## plan transition at your earliest convenience;
## some functionality will be moved to 'sp'.
```

```
library(PhyloMeasures)
library(raster)
library(rasterVis)
```

```
## Loading required package: terra
```

```
## terra version 1.3.22
```

```
##
## Attaching package: 'terra'
```

```
## The following objects are masked from 'package:dismo':
##
##     convHull, voronoi
```

```
## The following object is masked from 'package:ape':
##
##     trans
```

```
## Loading required package: lattice
```

```
## Loading required package: latticeExtra
```

```
##
## Attaching package: 'latticeExtra'
```

```
## The following object is masked from 'package:ggplot2':
##
##     layer
```

```
library(rgdal)
```

```
## Please note that rgdal will be retired by the end of 2023,
## plan transition to sf/stars/terra functions using GDAL and PROJ
## at your earliest convenience.
##
## rgdal: version: 1.5-27, (SVN revision 1148)
## Geospatial Data Abstraction Library extensions to R successfully loaded
## Loaded GDAL runtime: GDAL 3.2.1, released 2020/12/29
## Path to GDAL shared files: /usr/share/gdal
## GDAL binary built with GEOS: TRUE
## Loaded PROJ runtime: Rel. 7.2.1, January 1st, 2021, [PJ_VERSION: 721]
## Path to PROJ shared files: /home/kupeornis/.local/share/proj:/usr/share/proj
## PROJ CDN enabled: FALSE
## Linking to sp version:1.4-5
## To mute warnings of possible GDAL/OSR exportToProj4() degradation,
## use options("rgdal_show_exportToProj4_warnings"="none") before loading sp or rgdal.
```

```
##
## Attaching package: 'rgdal'
```

```
## The following object is masked from 'package:terra':
##
##     project
```

```r
library(rgeos)
```

```
## rgeos version: 0.5-7, (SVN revision 676)
##  GEOS runtime version: 3.9.0-CAPI-1.16.2
##  Please note that rgeos will be retired by the end of 2023,
## plan transition to sf functions using GEOS at your earliest convenience.
##  GEOS using OverlayNG
##  Linking to sp version: 1.4-5
##  Polygon checking: TRUE
```

```r
library(sf)
```

```
## Linking to GEOS 3.9.0, GDAL 3.2.1, PROJ 7.2.1
```

```r
library(sp)
library(tidyverse)
```

```
## -- Attaching packages --------------------------------------- tidyverse 1.3.1 --
```

```
## v tibble  3.1.4     v dplyr   1.0.7
## v tidyr   1.1.3     v stringr 1.4.0
## v readr   2.0.1     v forcats 0.5.1
## v purrr   0.3.4
```

```
## -- Conflicts ------------------------------------------ tidyverse_conflicts() --
## x dplyr::combine()      masks gridExtra::combine()
## x tidyr::extract()      masks terra::extract(), raster::extract()
## x dplyr::filter()       masks stats::filter()
## x dplyr::lag()          masks stats::lag()
## x latticeExtra::layer() masks ggplot2::layer()
## x dplyr::select()       masks raster::select()
## x dplyr::src()          masks terra::src()
```

```r
library(TreeSim)
```

```
## Loading required package: geiger
```

```
##
## Attaching package: 'geiger'
```

```
## The following object is masked from 'package:terra':
##
##     rescale
```

```
## The following object is masked from 'package:raster':
##
##     hdr
```

```r
library(viridis)
```

```
## Loading required package: viridisLite
```

2 Diversity Analyses

The following outlines the code required for analysing species diversity and phylogenetic diversity within out datset. *Note* that this code assumes the existence of the presence-absence matrices (PAMs) for birds and mammals.

2.1 Aggregating Names

The following chunk of code outlines how to strip and save names for downloading later on:

```
comm_coords=read_csv(paste0(filepath,"mammal_pam_coords.csv"))
mamm.dat=comm_coords%>%dplyr::select(-Var1,-Var2)
coords=comm_coords%>%dplyr::select(Var1,Var2)

bird.dat=read_csv(paste0(filepath,"fixed_birds_pam.csv"))

write_csv(coords,paste0(filepath,"raster_coords.csv"))
```

There are 1305 mammals and 2251 birds in this dataset.

```
names=colnames(mamm.dat)

write_csv(as.data.frame(names),paste0(filepath,"download_names.csv"))
```

2.2 Calculating metrics

2.2.1 Species Diversity

We can calculate species richness (α diversity) by calculating the row sums.

```
spdv.mamm<-rowSums(mamm.dat)
spdv.bird<-rowSums(bird.dat)
```

```
## Rows: 254361 Columns: 2

## -- Column specification --------------------------------------------------------
## Delimiter: ","
## dbl (2): spdv.mamm, spdv.bird

##
## i Use `spec()` to retrieve the full column specification for this data.
## i Specify the column types or set `show_col_types = FALSE` to quiet this message.

## Rows: 254361 Columns: 2

## -- Column specification --------------------------------------------------------
## Delimiter: ","
## dbl (2): Var1, Var2

##
## i Use `spec()` to retrieve the full column specification for this data.
## i Specify the column types or set `show_col_types = FALSE` to quiet this message.
```

We can vizualise cell counts by richness to compare the two distributions using `gghistogram`.

```
a=gghistogram(spdv.mamm,add="mean",
              rug=F,#add_density=T,
              bins=250) +
  xlab("Species Diversity")+
  labs(title="Mammal Richness")+
  scale_y_continuous(limits=c(0,11000))
```

```
## Warning: geom_vline(): Ignoring `mapping` because `xintercept` was provided.

## Warning: geom_vline(): Ignoring `data` because `xintercept` was provided.
```

```
b=gghistogram(spdv.bird,add="mean",
              rug=F,#add_density=T,
              bins=250) +
  xlab("Species Diversity")+
  labs(title="Bird Richness")+
  scale_y_continuous(limits=c(0,11000))
```

```
## Warning: geom_vline(): Ignoring `mapping` because `xintercept` was provided.

## Warning: geom_vline(): Ignoring `data` because `xintercept` was provided.
```

```
grid.arrange(a,b,ncol=2)
```

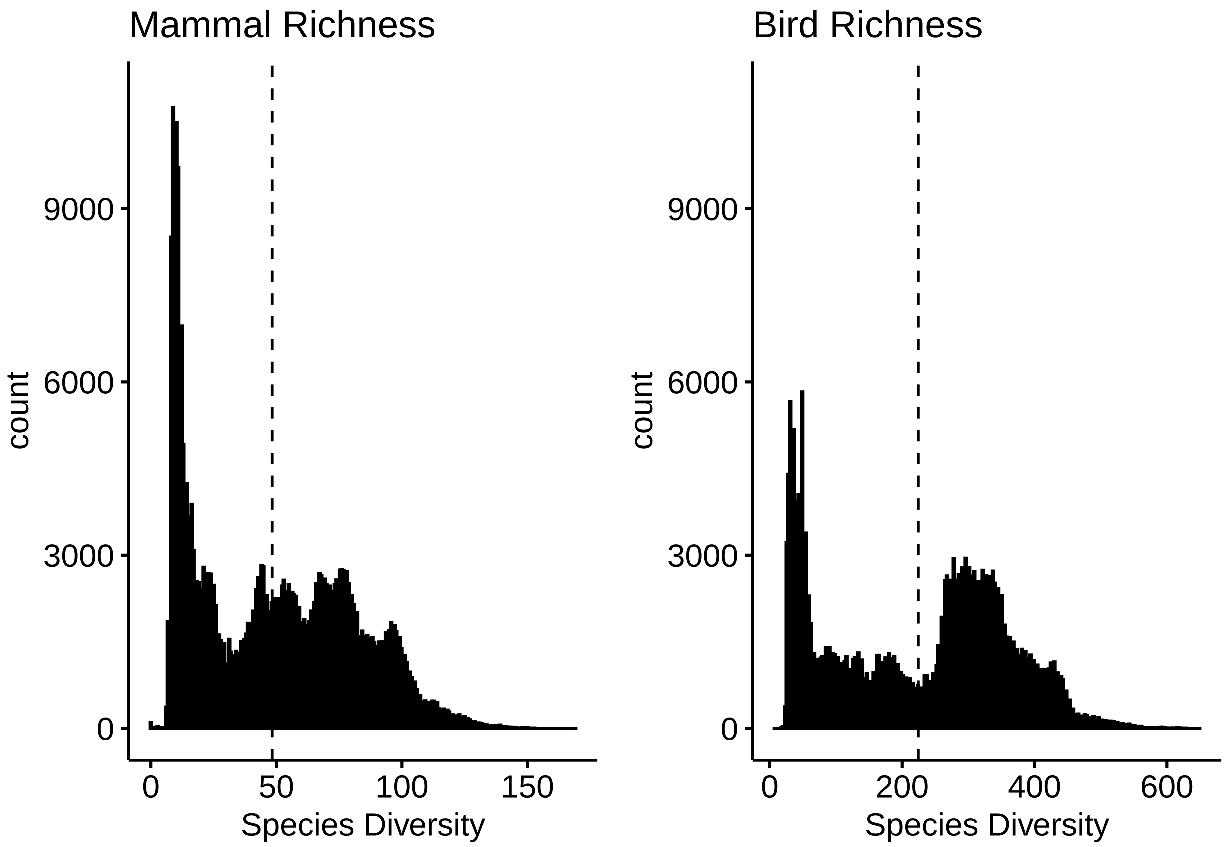

We can see that the general distributions have some similarities, even if diversity and count have variations.

We can now compare species diversity for mammals and birds. *Note* that this code also saves copies of the files separately for use in the manuscript.

```
spdv.mamm.dat=cbind(coords,spdv.mamm)
spdv.bird.dat=cbind(coords,spdv.bird)
```

```
a=ggplot()+
  geom_raster(data=spdv.mamm.dat,
              aes(x=Var1,y=Var2,fill=spdv.mamm))+
  labs(x="Longitude",y="Latitude",fill="Richness",title="Mammal Richness")+
  scale_fill_viridis_c()+
  theme_classic()+
  theme(plot.title=element_text(hjust=0.5))+
```

```
  coord_quickmap()

b=ggplot()+
  geom_raster(data=spdv.bird.dat,
              aes(x=Var1,y=Var2,fill=spdv.bird))+
  labs(x="Longitude",y="Latitude",fill="Richness",title="Bird Richness")+
  scale_fill_viridis_c()+
  theme_classic()+
  theme(plot.title=element_text(hjust=0.5))+
  coord_quickmap()
```

```
grid.arrange(a,b,ncol=2)
```

```
## Warning: Raster pixels are placed at uneven horizontal intervals and will be
## shifted. Consider using geom_tile() instead.

## Warning: Raster pixels are placed at uneven horizontal intervals and will be
## shifted. Consider using geom_tile() instead.
```

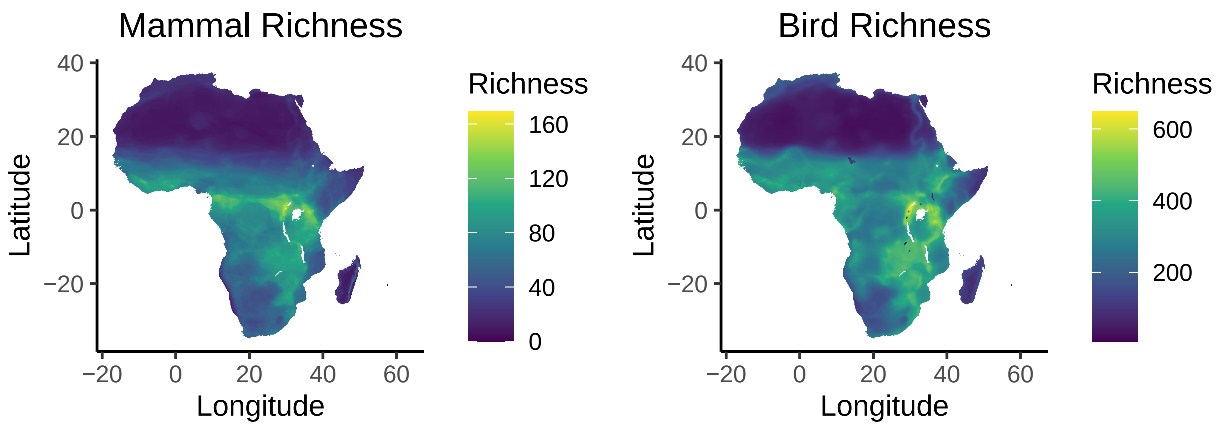


```
# mammal
a=ggplot()+
  geom_raster(data=spdv.mamm.dat,
              aes(x=Var1,y=Var2,fill=spdv.mamm))+
  labs(x="Longitude",y="Latitude",fill="Richness")+
  scale_fill_viridis_c()+
  theme_classic()+
```

```
  coord_quickmap()
# bird
b=ggplot()+
  geom_raster(data=spdv.bird.dat,
              aes(x=Var1,y=Var2,fill=spdv.bird))+
  labs(x="Longitude",y="Latitude",fill="Richness")+
  scale_fill_viridis_c()+
  theme_classic()+
  coord_quickmap()

ggsave(paste0(filepath,"mammal_richness.png"),a,dpi=400)
ggsave(paste0(filepath,"bird_richness.png"),b,dpi=400)
```

Now we can rasterize these and compare them directly. First we have to normalize a vector x between zero and one by using the equation $\frac{x'-min(x')}{max(x')-min(x')}$.

```
# normalize values

normalize=function(x){
  max=max(x)
  min=min(x)
  out=(x-min)/(max-min)
  return(out)
}

spdv.mamm.stand=spdv.mamm.dat

spdv.mamm.stand$spdv.mamm=normalize(spdv.mamm.stand$spdv.mamm)

spdv.bird.stand=spdv.bird.dat

spdv.bird.stand$spdv.bird=normalize(spdv.bird.stand$spdv.bird)

# rasterize

spdv.mamm.rast=rasterFromXYZ(spdv.mamm.stand)
spdv.bird.rast=rasterFromXYZ(spdv.bird.stand)
```

Now we also need to create random comparisons for the raster to determine significance.

```
# function specialized here, uses third (data) column

randomize=function(df,seed){
  #set.seed(81507)
  set.seed(seed)
  df.x=df[,3]
  for(i in 1:100){
    rando=sample(df.x)
    df[,(i+3)]=rando
  }

  cols.x=paste0(rep('random.',100),1:100)
  colnames(df)[-c(1:3)]=cols.x

  return(df)
```

```
}

spdv.mamm.random=randomize(spdv.mamm.stand,81507)
spdv.bird.random=randomize(spdv.bird.stand,62901)

spdv.mamm.rast=rasterFromXYZ(spdv.mamm.random)
spdv.bird.rast=rasterFromXYZ(spdv.bird.random)
```

Now, we can calculate the similarities using the real rasters and the random rasters. We are going to do this with a custom function.

```
# metric is "I" or "D"
# stack always has [[1]] as the 'real' layer, 2-101 random
# category is metric being compared
raster.compare=function(stack1,stack2,metric,category){
  mamm.stats=NULL
  bird.stats=NULL
  real.mamm=spdv.mamm.rast[[1]]
  real.bird=spdv.bird.rast[[1]]

  # Raster similarity

  # similar to niche comparison tests
  # need to test the difference of each to random rasters
  # need to compare distributions to the 'real' difference

  for(i in 2:101){
    # get randomized rasters
    r1=spdv.mamm.rast[[i]]
    r2=spdv.bird.rast[[i]]
    # perform comparisons
    # compare mamm to bird random
    mamm.stats[i-1]=nicheOverlap(real.mamm,r2,stat=metric)
    # compare bird to mamm random
    bird.stats[i-1]=nicheOverlap(real.bird,r1,stat=metric)
  }

  test.stat=nicheOverlap(real.mamm,real.bird,stat=metric)

  print(paste0("Test: Mammals vs. Random Birds: ",category))
  print(t.test(x=mamm.stats,mu=test.stat,alternative="two.sided"))
  print(paste0("Test: ",test.stat))

  print(paste0("Test: Birds vs. Random Mammals",category))
  print(t.test(x=mamm.stats,mu=test.stat,alternative="two.sided"))
  print(paste0("Test: ",test.stat))

  print("Visualization:")

  df.x=c(mamm.stats,bird.stats)
  labs.x=c(rep("mammals",100),rep("birds",100))

  df.x=as.data.frame(cbind(labs.x,df.x))
  colnames(df.x)=c("Group","Value")
```

```r
  df.x$Value=as.numeric(df.x$Value)
  df.x$Group=as.factor(df.x$Group)

  a=ggplot(data=df.x,aes(x=Value,fill=Group))
  b=scale_x_continuous(limits=c(0.5,1))
  b.5=geom_density(alpha=0.6)
  c=theme_classic()
  d=geom_vline(xintercept=test.stat,colour="black",linetype="dashed")

  print(a+b+b.5+c+d)
}
```

```r
raster.compare(stack1=spdv.mamm.rast,stack2=spdv.bird.rast,metric="I",category="Richness")
```

```
## [1] "Test: Mammals vs. Random Birds: Richness"
##
##  One Sample t-test
##
## data:  mamm.stats
## t = -4845.8, df = 99, p-value < 2.2e-16
## alternative hypothesis: true mean is not equal to 0.9923956
## 95 percent confidence interval:
##  0.8737611 0.8738582
## sample estimates:
## mean of x
## 0.8738096
##
## [1] "Test: 0.992395637641325"
## [1] "Test: Birds vs. Random MammalsRichness"
##
##  One Sample t-test
##
## data:  mamm.stats
## t = -4845.8, df = 99, p-value < 2.2e-16
## alternative hypothesis: true mean is not equal to 0.9923956
## 95 percent confidence interval:
##  0.8737611 0.8738582
## sample estimates:
## mean of x
## 0.8738096
##
## [1] "Test: 0.992395637641325"
## [1] "Visualization:"
```

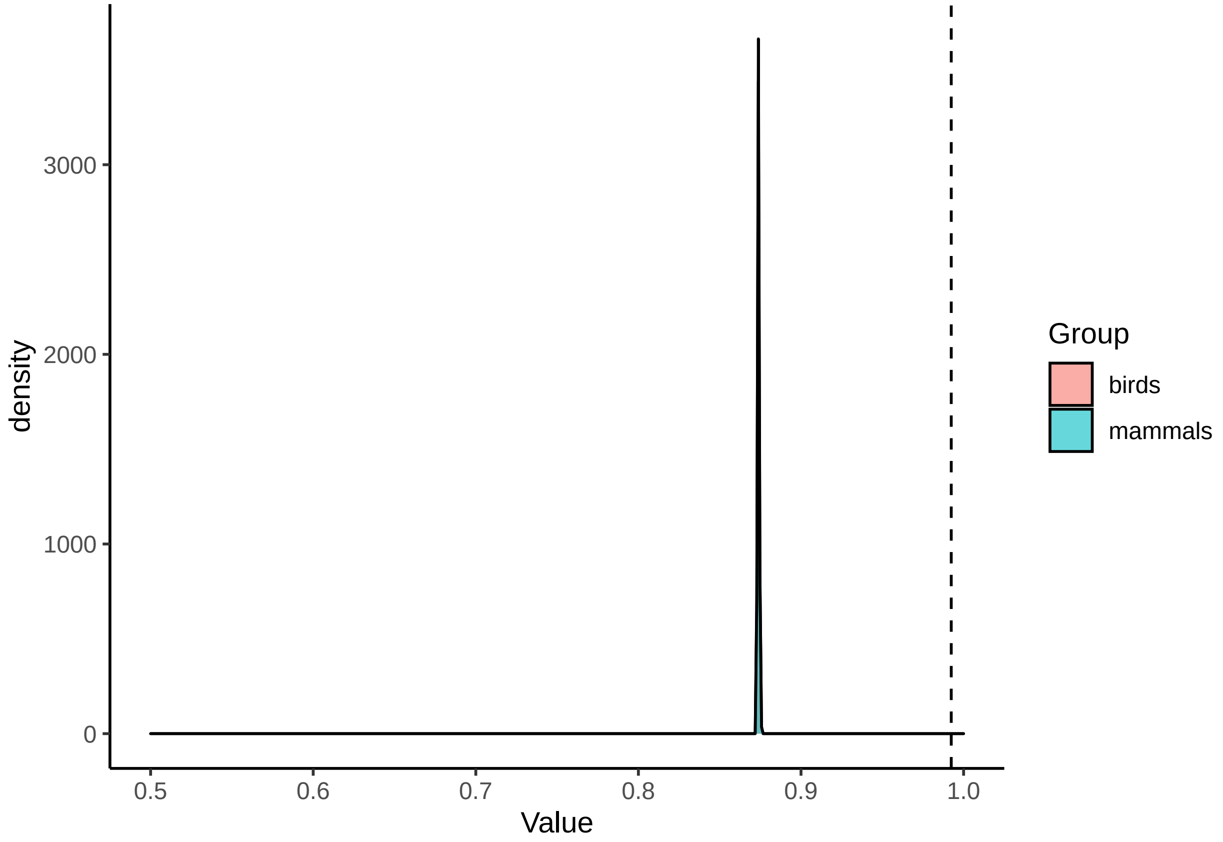


```
raster.compare(stack1=spdv.mamm.rast,stack2=spdv.bird.rast,metric="D",category="Richness")
```

```
## [1] "Test: Mammals vs. Random Birds: Richness"
##
##  One Sample t-test
##
## data:  mamm.stats
## t = -6299.3, df = 99, p-value < 2.2e-16
## alternative hypothesis: true mean is not equal to 0.9065873
## 95 percent confidence interval:
##  0.6314348 0.6316080
## sample estimates:
## mean of x
## 0.6315214
##
## [1] "Test: 0.906587331161116"
## [1] "Test: Birds vs. Random MammalsRichness"
##
##  One Sample t-test
##
## data:  mamm.stats
## t = -6299.3, df = 99, p-value < 2.2e-16
## alternative hypothesis: true mean is not equal to 0.9065873
## 95 percent confidence interval:
##  0.6314348 0.6316080
## sample estimates:
```

```
## mean of x
## 0.6315214
##
## [1] "Test: 0.906587331161116"
## [1] "Visualization:"
```

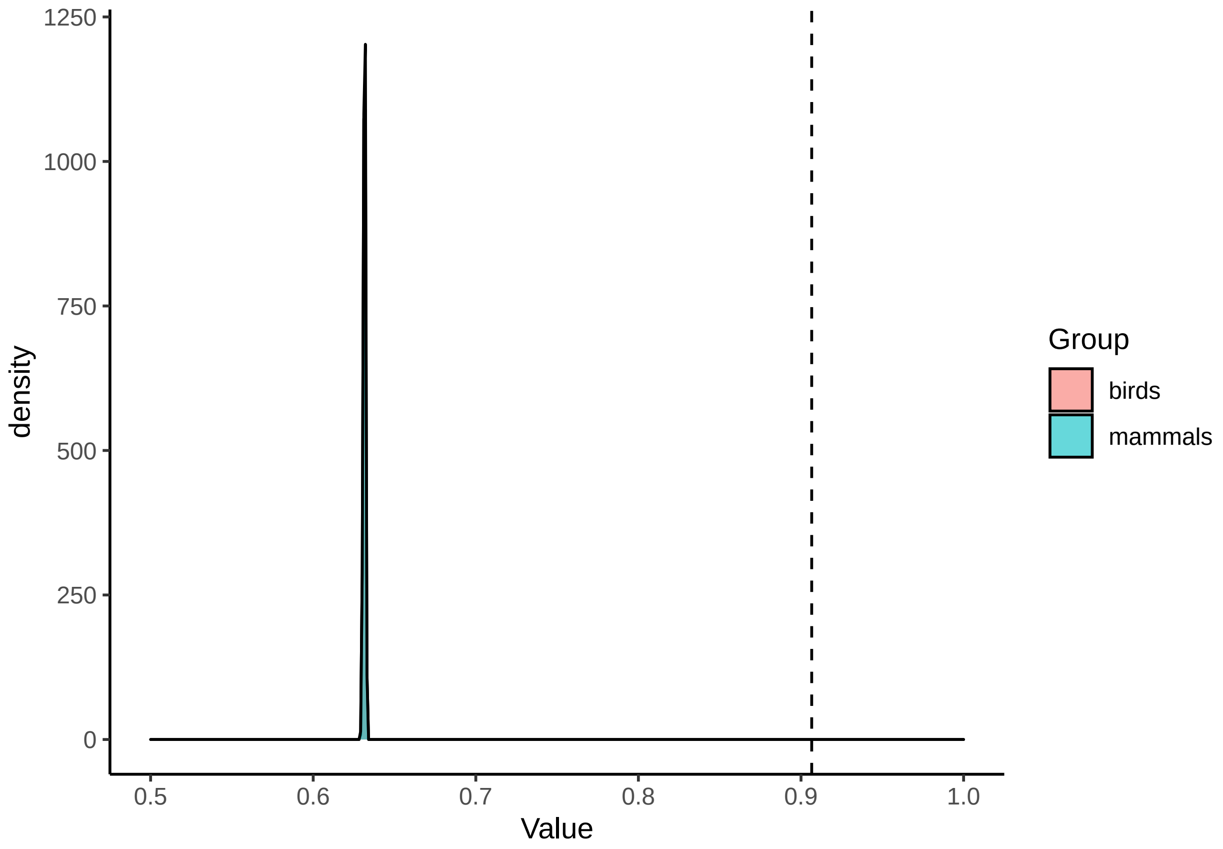

These distributions are overall quite similar.

We can also do a linear regression of the similarity between these groups.

```
compareddf=cbind(spdv.mamm.dat,spdv.bird.dat$spdv.bird)%>%as.data.frame()

colnames(compareddf)=c("Long","Lat","Mamm","Bird")

df.lm=lm(Bird~Mamm,compareddf)

summary(df.lm)
```

```
##
## Call:
## lm(formula = Bird ~ Mamm, data = compareddf)
##
## Residuals:
##     Min      1Q  Median      3Q     Max
## -528.35  -36.22   -7.93   35.69  200.96
##
## Coefficients:
##             Estimate Std. Error t value Pr(>|t|)
```

```
## (Intercept) 36.584018   0.189605   192.9   <2e-16 ***
## Mamm         3.886484   0.003272  1187.9   <2e-16 ***
## ---
## Signif. codes:  0 '***' 0.001 '**' 0.01 '*' 0.05 '.' 0.1 ' ' 1
##
## Residual standard error: 52.84 on 254359 degrees of freedom
## Multiple R-squared:  0.8473, Adjusted R-squared:  0.8473
## F-statistic: 1.411e+06 on 1 and 254359 DF,  p-value: < 2.2e-16
```

```
cor(comparedf[,-c(1:2)])
```

```
##           Mamm      Bird
## Mamm 1.0000000 0.9204788
## Bird 0.9204788 1.0000000
```

```
# with a standard linear regression

a=ggplot(comparedf,aes(x=Mamm,y=Bird))+
  geom_point(size=.05)+
  geom_smooth(method='lm',formula=y~x)+
  theme_classic()+
  xlab("Mammals")+ylab("Birds")

a
```

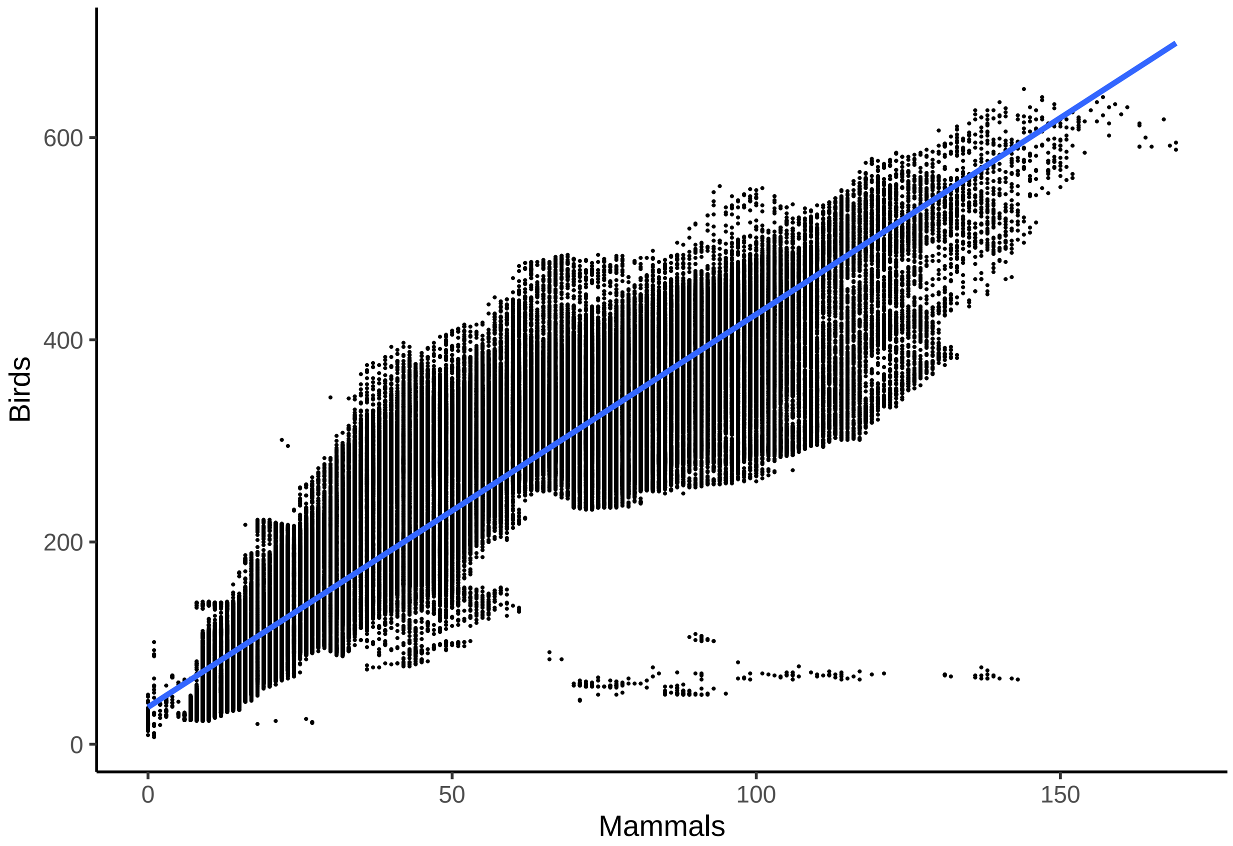


```
ggsave(filename=paste0(filepath,"richness_regression.png"),a,dpi=400)
```

```
## Saving 6.5 x 4.5 in image
```

```
# with a loess fit

a=ggplot(comparedf,aes(x=Mamm,y=Bird))+
  geom_point(size=.05)+
  geom_smooth()+
  theme_classic()

a
```

```
## `geom_smooth()` using method = 'gam' and formula 'y ~ s(x, bs = "cs")'
```

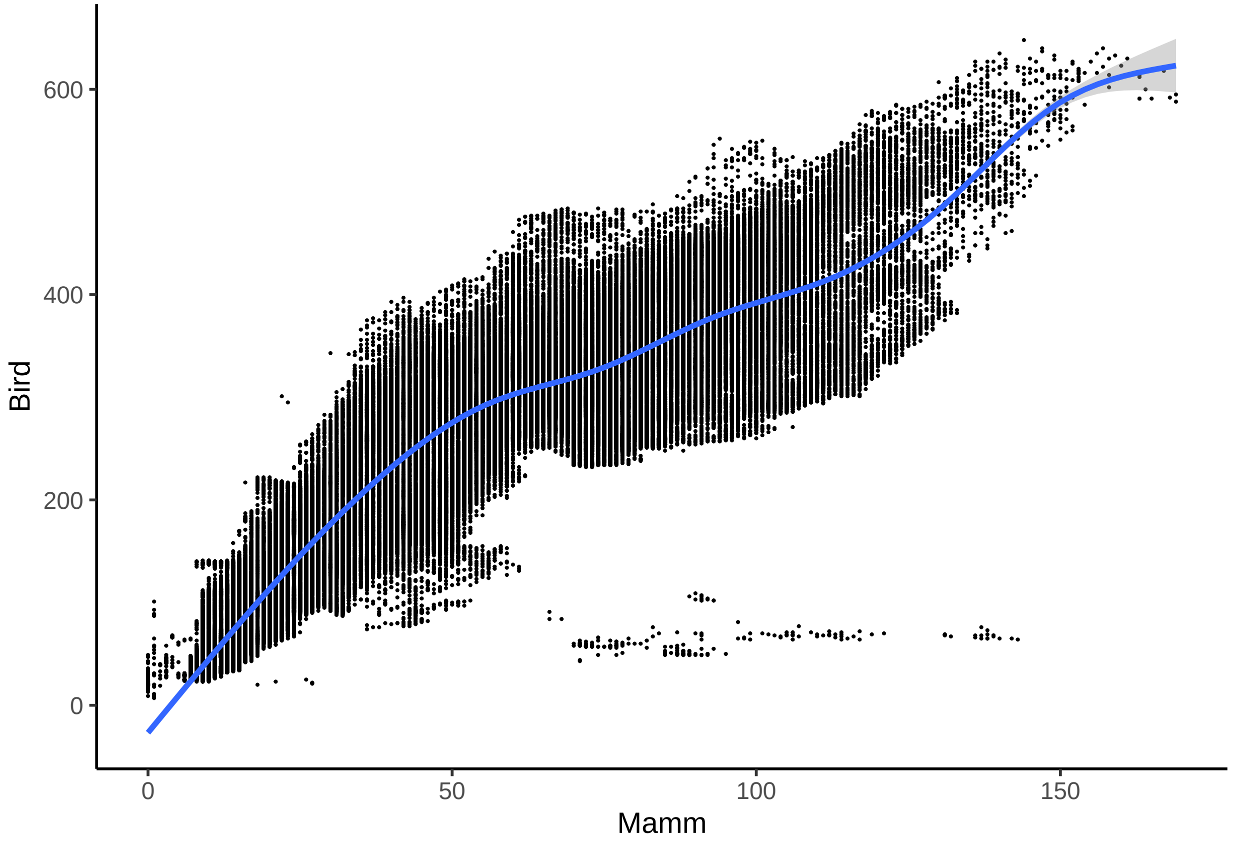

There are a lot of outliers with high mammal diversity and low bird diversity. We can identify these points spatially.

```
outliers=comparedf%>%filter(Mamm>60,Bird<125)

plot(comparedf$Long,comparedf$Lat,pch=".",col="black",asp=1,
     ylab="Latitude",xlab="Longitude")
points(outliers$Long,outliers$Lat,pch=19,col="red")
```

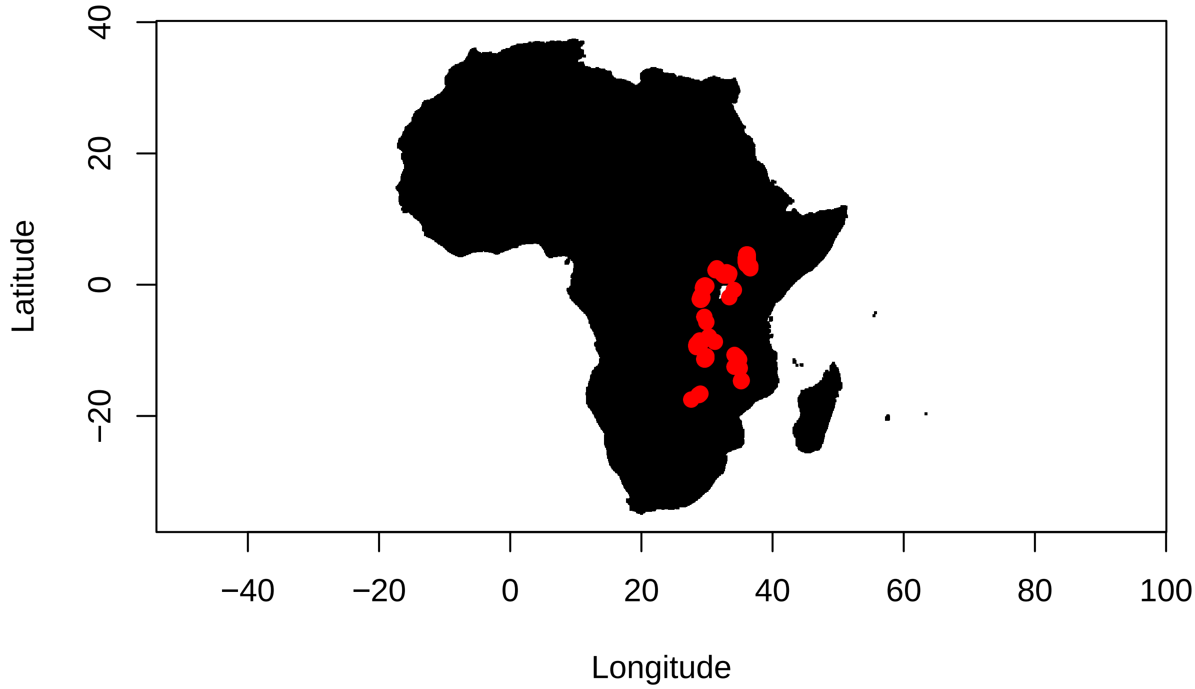


```
print(paste0("There are ",nrow(outliers),
             " outliers. This represents ",round(nrow(outliers)/nrow(comparedf),6),"%."))
```

```
## [1] "There are 202 outliers. This represents 0.000794%."
```

These areas in the rift have disproportionately higher mammal diversity than bird diversity. These areas are all near water bodies, and are almost certainly issues with how the maps are made.

Other Visualizations We visualized these data a few other ways as well.

```
coordinates(spdv.mamm.dat) <- c("Var1","Var2")
gridded(spdv.mamm.dat) <- TRUE
```

```
## Warning in points2grid(points, tolerance, round): grid has empty column/rows in
## dimension 1
```

```
coordinates(spdv.bird.dat) <- c("Var1","Var2")
gridded(spdv.bird.dat) <- TRUE
```

```
## Warning in points2grid(points, tolerance, round): grid has empty column/rows in
## dimension 1
```

```
spdv.dat.sgdf.mamm <- as(spdv.mamm.dat, "SpatialGridDataFrame")
spdv.dat.sgdf.bird <- as(spdv.bird.dat, "SpatialGridDataFrame")
```

```
n=max(na.omit(spdv.dat.sgdf.bird$spdv.bird))
bird.map=raster(spdv.dat.sgdf.bird)

N=max(na.omit(spdv.dat.sgdf.mamm$spdv.mamm))
```

```
mamm.map=raster(spdv.dat.sgdf.mamm)
```

```
levelplot(bird.map)
```

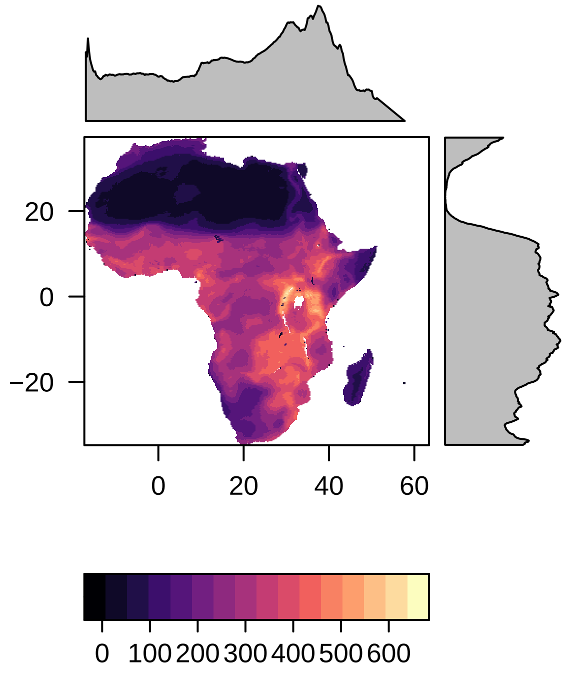


```
levelplot(mamm.map)
```

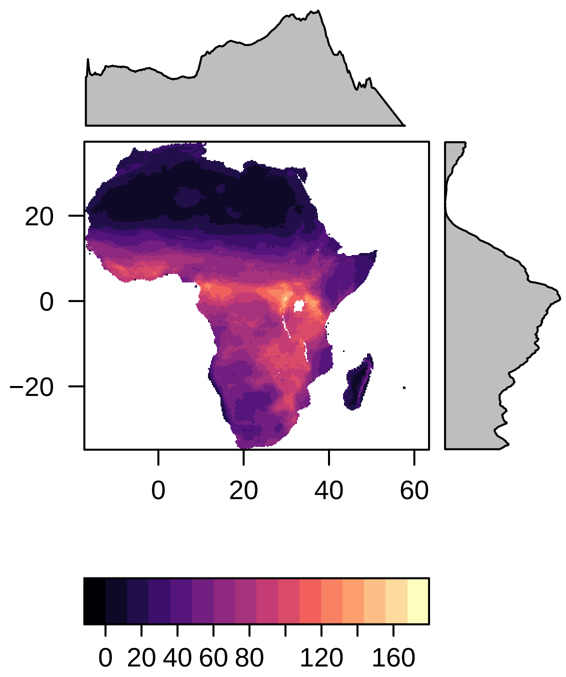


```
writeRaster(bird.map,paste0(filepath,"bird_richness.asc"))
writeRaster(mamm.map,paste0(filepath,"mamm_richness.asc"))
```

2.2.2 Mean Phylogenetic Distance

```
mpd.df.mamm=read_csv(paste0(filepath,"all.mpd.runs.csv"))
mpd.df.bird=read_csv(paste0(filepath,"all.mpd.runs_birds.csv"))

mean.mpd.mamm <- apply(mpd.df.mamm, 1, mean)
mean.mpd.bird <- apply(mpd.df.bird, 1, mean)

# Some cells have 0 or 1 species present -- therefore create 0 values
length(mean.mpd.mamm[mean.mpd.mamm == 0])
length(mean.mpd.bird[mean.mpd.bird == 0])
```

```
## Rows: 254361 Columns: 2
```

```
## -- Column specification --------------------------------------------------------
## Delimiter: ","
## dbl (2): mean.mpd.mamm, mean.mpd.bird
```

```
##
## i Use `spec()` to retrieve the full column specification for this data.
## i Specify the column types or set `show_col_types = FALSE` to quiet this message.
```

```r
# bind MPD data to coordinates

mpd.mamm.dat=cbind(coords,mean.mpd.mamm)
mpd.bird.dat=cbind(coords,mean.mpd.bird)
```

```r
a=ggplot()+
  geom_raster(data=mpd.mamm.dat,
              aes(x=Var1,y=Var2,fill=mean.mpd.mamm))+
  labs(x="Longitude",y="Latitude",fill="MPD",title="Mammal MPD")+
  scale_fill_viridis_c(option="inferno")+
  theme_classic()+
  theme(plot.title=element_text(hjust=0.5))+
  coord_quickmap()

b=ggplot()+
  geom_raster(data=mpd.bird.dat,
              aes(x=Var1,y=Var2,fill=mean.mpd.bird))+
  labs(x="Longitude",y="Latitude",fill="MPD",title="Bird MPD")+
  scale_fill_viridis_c(option="inferno")+
  theme_classic()+
  theme(plot.title=element_text(hjust=0.5))+
  coord_quickmap()
```

```r
grid.arrange(a,b,ncol=2)
```

```
## Warning: Raster pixels are placed at uneven horizontal intervals and will be
## shifted. Consider using geom_tile() instead.

## Warning: Raster pixels are placed at uneven horizontal intervals and will be
## shifted. Consider using geom_tile() instead.
```

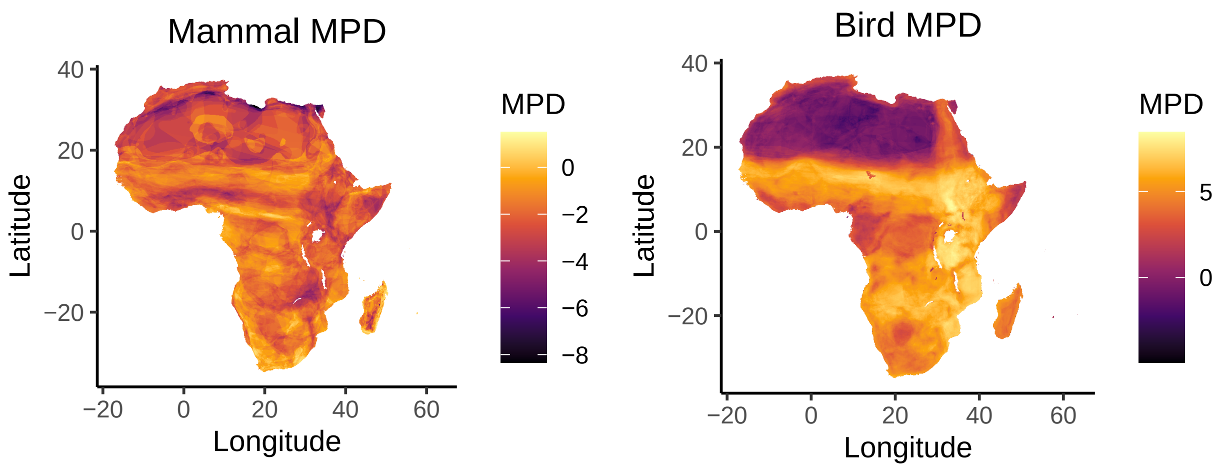


```
# save plots
# mammal
a=ggplot()+
  geom_raster(data=mpd.mamm.dat,
              aes(x=Var1,y=Var2,fill=mean.mpd.mamm))+
  labs(x="Longitude",y="Latitude",fill="MPD")+
  scale_fill_viridis_c(option="inferno")+
  theme_classic()+
  #theme(plot.title=element_text(hjust=0.5))+
  coord_quickmap()
# bird
b=ggplot()+
  geom_raster(data=mpd.bird.dat,
              aes(x=Var1,y=Var2,fill=mean.mpd.bird))+
  labs(x="Longitude",y="Latitude",fill="MPD")+
  scale_fill_viridis_c(option="inferno")+
  theme_classic()+
  #theme(plot.title=element_text(hjust=0.5))+
  coord_quickmap()

ggsave(paste0(filepath,"mpd_mammal.png"),a,dpi=400)
```

```
## Saving 6.5 x 4.5 in image
```

```
## Warning: Raster pixels are placed at uneven horizontal intervals and will be
## shifted. Consider using geom_tile() instead.
```

```r
ggsave(paste0(filepath,"mpd_bird.png"),b,dpi=400)
```

```
## Saving 6.5 x 4.5 in image
## Warning: Raster pixels are placed at uneven horizontal intervals and will be
## shifted. Consider using geom_tile() instead.
```

Now we can rasterize these and compare them directly. First we have to normalize a vector x between zero and one by using the equation $\frac{x'-min(x')}{max(x')-min(x')}$.

```r
# standardize values

mpd.mamm.stand=mpd.mamm.dat
mpd.bird.stand=mpd.bird.dat

mpd.mamm.stand$mean.mpd.mamm=normalize(mpd.mamm.stand$mean.mpd.mamm)
mpd.bird.stand$mean.mpd.bird=normalize(mpd.bird.stand$mean.mpd.bird)

# randomize

mpd.mamm.random=randomize(mpd.mamm.stand,seed=81507)
mpd.bird.random=randomize(mpd.bird.stand,seed=62901)

# rasterize

mpd.mamm.rast=rasterFromXYZ(mpd.mamm.random)
mpd.bird.rast=rasterFromXYZ(mpd.bird.random)
```

```r
raster.compare(stack1=mpd.mamm.rast,stack2=mpd.bird.rast,metric="I",category="Mean Phylogenetic Distanc
```

```
## [1] "Test: Mammals vs. Random Birds: Mean Phylogenetic Distance"
##
##  One Sample t-test
##
## data:  mamm.stats
## t = -4845.8, df = 99, p-value < 2.2e-16
## alternative hypothesis: true mean is not equal to 0.9923956
## 95 percent confidence interval:
##  0.8737611 0.8738582
## sample estimates:
## mean of x
## 0.8738096
##
## [1] "Test: 0.992395637641325"
## [1] "Test: Birds vs. Random MammalsMean Phylogenetic Distance"
##
##  One Sample t-test
##
## data:  mamm.stats
## t = -4845.8, df = 99, p-value < 2.2e-16
## alternative hypothesis: true mean is not equal to 0.9923956
## 95 percent confidence interval:
##  0.8737611 0.8738582
## sample estimates:
## mean of x
## 0.8738096
```

```
##
## [1] "Test: 0.992395637641325"
## [1] "Visualization:"
```

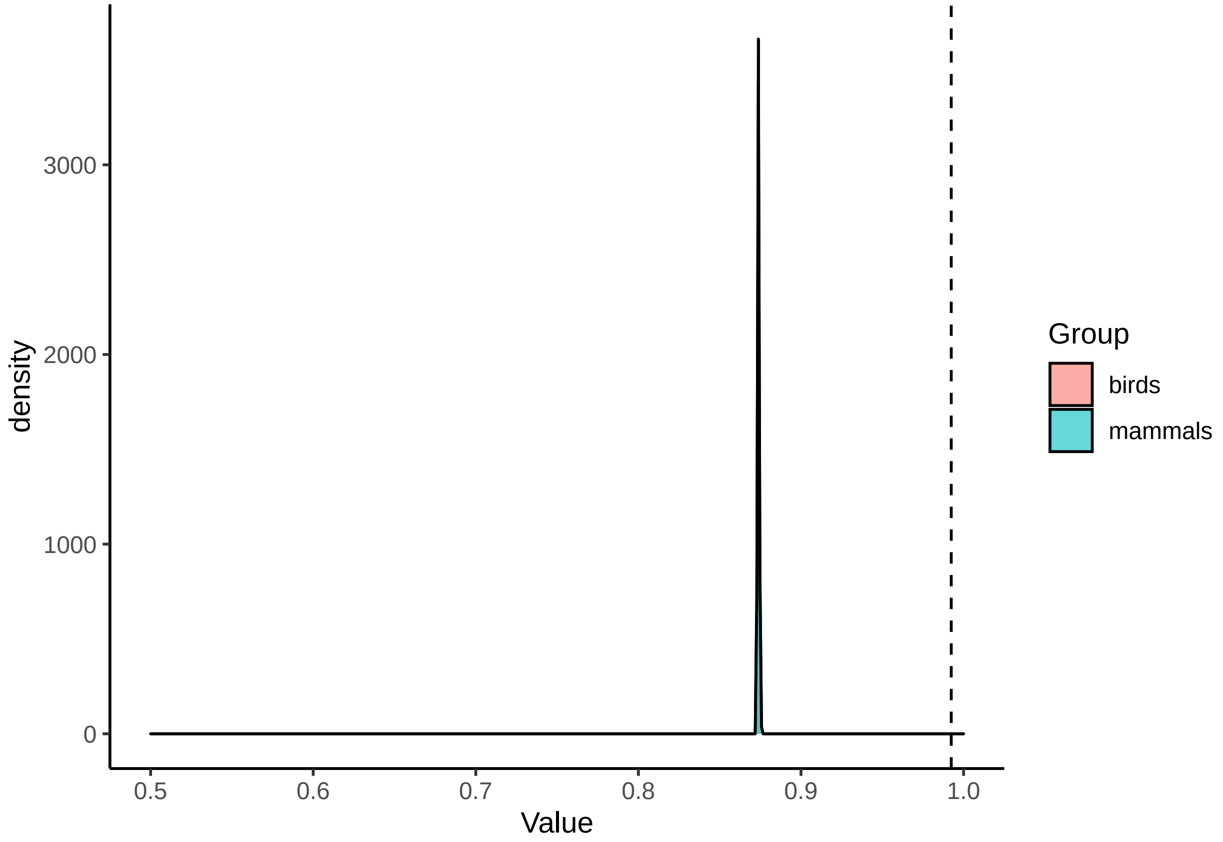


```
raster.compare(stack1=mpd.mamm.rast,stack2=mpd.bird.rast,metric="D",category="Mean Phylogenetic Distanc
```

```
## [1] "Test: Mammals vs. Random Birds: Mean Phylogenetic Distance"
##
##  One Sample t-test
##
## data:  mamm.stats
## t = -6299.3, df = 99, p-value < 2.2e-16
## alternative hypothesis: true mean is not equal to 0.9065873
## 95 percent confidence interval:
##  0.6314348 0.6316080
## sample estimates:
## mean of x
## 0.6315214
##
## [1] "Test: 0.906587331161116"
## [1] "Test: Birds vs. Random MammalsMean Phylogenetic Distance"
##
##  One Sample t-test
##
## data:  mamm.stats
## t = -6299.3, df = 99, p-value < 2.2e-16
## alternative hypothesis: true mean is not equal to 0.9065873
```

```
## 95 percent confidence interval:
##  0.6314348 0.6316080
## sample estimates:
## mean of x
## 0.6315214
##
## [1] "Test: 0.906587331161116"
## [1] "Visualization:"
```

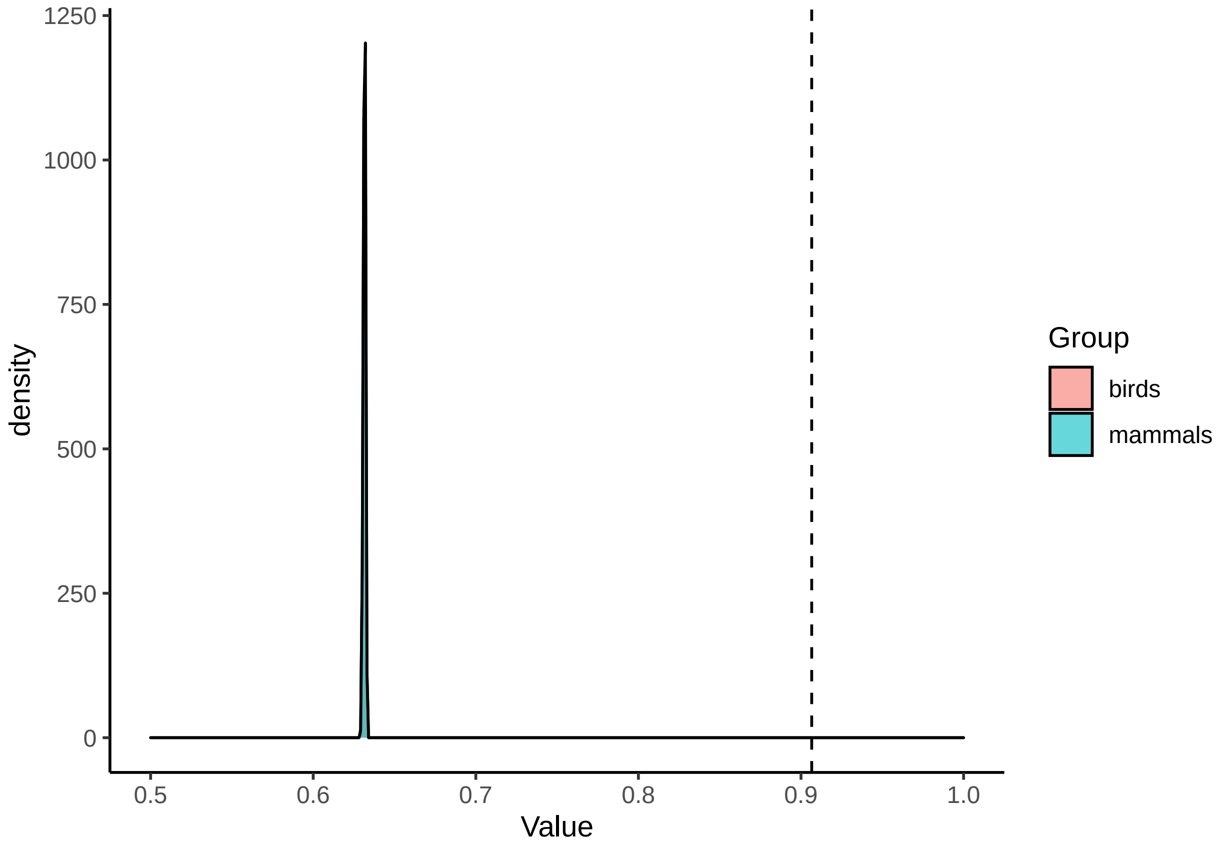

These distributions are overall quite similar still, when compared using these raster metrics.

```
comparedf=cbind(mpd.mamm.dat,mpd.bird.dat$mean.mpd.bird)%>%as.data.frame()

colnames(comparedf)=c("Long","Lat","Mamm","Bird")

df.lm=lm(Bird~Mamm,comparedf)

summary(df.lm)
```

```
##
## Call:
## lm(formula = Bird ~ Mamm, data = comparedf)
##
## Residuals:
##      Min       1Q   Median       3Q      Max
## -10.4016  -1.9072   0.1771   2.0161   6.3732
##
```

```
## Coefficients:
##             Estimate Std. Error t value Pr(>|t|)
## (Intercept) 5.474186   0.010811   506.4   <2e-16 ***
## Mamm        0.896045   0.004693   190.9   <2e-16 ***
## ---
## Signif. codes:  0 '***' 0.001 '**' 0.01 '*' 0.05 '.' 0.1 ' ' 1
##
## Residual standard error: 2.476 on 254359 degrees of freedom
## Multiple R-squared:  0.1254, Adjusted R-squared:  0.1254
## F-statistic: 3.646e+04 on 1 and 254359 DF,  p-value: < 2.2e-16
```

```
cor(comparedf[,-c(1:2)])
```

```
##           Mamm      Bird
## Mamm 1.0000000 0.3540723
## Bird 0.3540723 1.0000000
```

```
a=ggplot(comparedf,aes(x=Mamm,y=Bird))+
  geom_point(size=.05)+
  geom_smooth(method="lm",formula=y~x)+
  theme_classic()

a
```

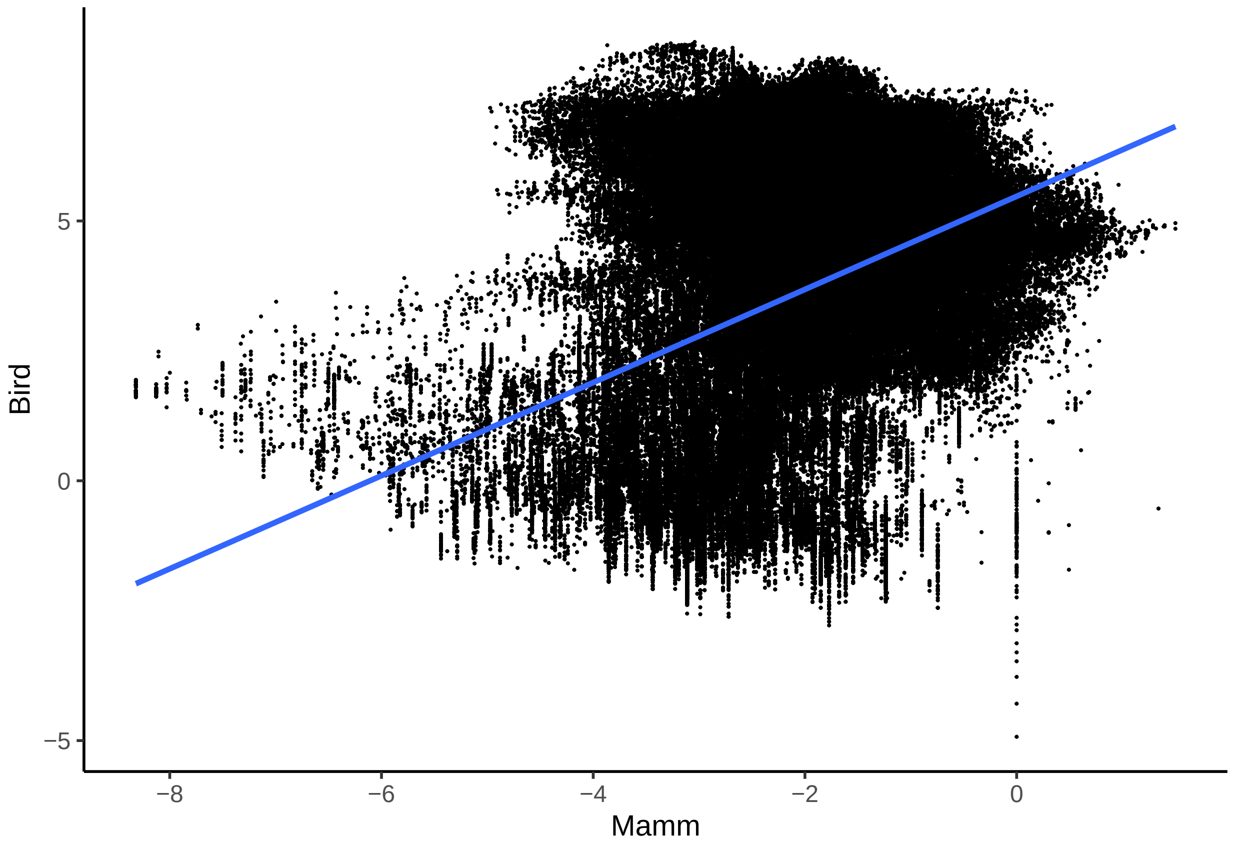

Range metrics Calculating the range of MPD values.

```
range.diff=function(x){
  result <- diff(range(x, na.rm=T))
```

```
  return(result)
}
```

```
range.mamm.mpd=apply(mpd.df.mamm,1,range.diff)
range.bird.mpd=apply(mpd.df.bird,1,range.diff)
```

```
## Rows: 254361 Columns: 2

## -- Column specification --------------------------------------------------------
## Delimiter: ","
## dbl (2): range.mamm.mpd, range.bird.mpd

##
## i Use `spec()` to retrieve the full column specification for this data.
## i Specify the column types or set `show_col_types = FALSE` to quiet this message.
```

```
mpd.mamm.dat2=cbind(coords,range.mamm.mpd)
mpd.bird.dat2=cbind(coords,range.bird.mpd)
```

```
a=ggplot()+
  geom_raster(data=mpd.mamm.dat2,
              aes(x=Var1,y=Var2,fill=range.mamm.mpd))+
  labs(x="Longitude",y="Latitude",fill="MPD",title="Mammal MPD Range")+
  scale_fill_viridis_c(option="inferno")+
  theme_classic()+
  theme(plot.title=element_text(hjust=0.5))+
  coord_quickmap()

b=ggplot()+
  geom_raster(data=mpd.bird.dat2,
              aes(x=Var1,y=Var2,fill=range.bird.mpd))+
  labs(x="Longitude",y="Latitude",fill="MPD",title="Bird MPD Range")+
  scale_fill_viridis_c(option="inferno")+
  theme_classic()+
  theme(plot.title=element_text(hjust=0.5))+
  coord_quickmap()
```

```
grid.arrange(a,b,ncol=2)
```

```
## Warning: Raster pixels are placed at uneven horizontal intervals and will be
## shifted. Consider using geom_tile() instead.

## Warning: Raster pixels are placed at uneven horizontal intervals and will be
## shifted. Consider using geom_tile() instead.
```

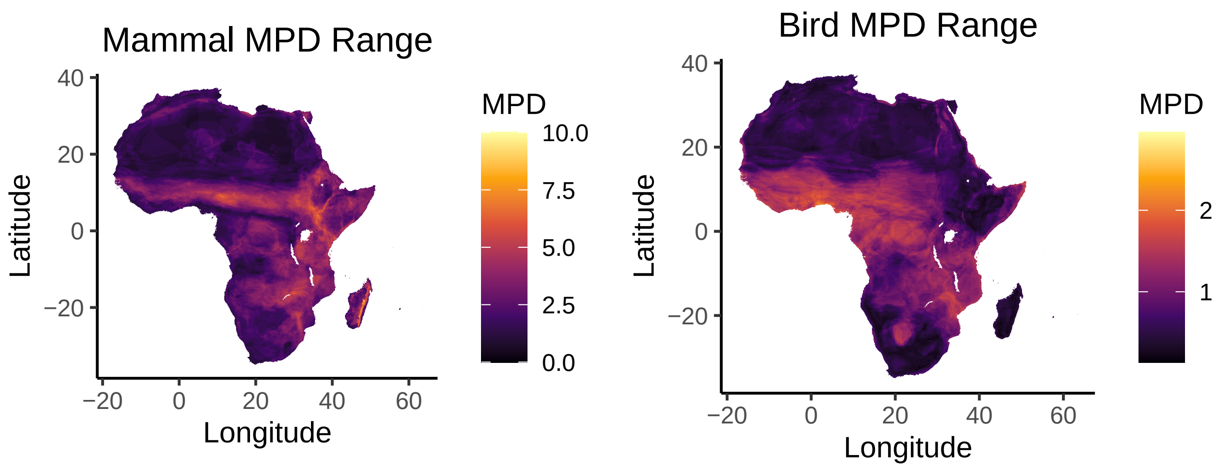

2.2.3 Mean Nearest Taxon Distance

```
mntd.mamm=read_csv(paste0(filepath,"all.mntd.runs.csv"))
mntd.bird=read_csv(paste0(filepath,"all.mntd.runs_birds.csv"))

# mean mntd
mean.mntd.mamm=apply(mntd.mamm,1,mean)
mean.mntd.bird=apply(mntd.bird,1,mean)
```

```
## Rows: 254361 Columns: 2

## -- Column specification --------------------------------------------------------
## Delimiter: ","
## dbl (2): mean.mntd.mamm, mean.mntd.bird

##
## i Use `spec()` to retrieve the full column specification for this data.
## i Specify the column types or set `show_col_types = FALSE` to quiet this message.
```

```
mntd.mamm.dat=cbind(coords,mean.mntd.mamm)
mntd.bird.dat=cbind(coords,mean.mntd.bird)
```

```
a=ggplot()+
  geom_raster(data=mntd.mamm.dat,
              aes(x=Var1,y=Var2,fill=mean.mntd.mamm))+
  labs(x="Longitude",y="Latitude",fill="MNTD",title="Mammal MNTD")+
  scale_fill_viridis_c(option="cividis")+
```

```
  theme_classic()+
  theme(plot.title=element_text(hjust=0.5))+
  coord_quickmap()

b=ggplot()+
  geom_raster(data=mntd.bird.dat,
              aes(x=Var1,y=Var2,fill=mean.mntd.bird))+
  labs(x="Longitude",y="Latitude",fill="MNTD",title="Bird MNTD")+
  scale_fill_viridis_c(option="cividis")+
  theme_classic()+
  theme(plot.title=element_text(hjust=0.5))+
  coord_quickmap()
```

```
grid.arrange(a,b,ncol=2)
```

```
## Warning: Raster pixels are placed at uneven horizontal intervals and will be
## shifted. Consider using geom_tile() instead.

## Warning: Raster pixels are placed at uneven horizontal intervals and will be
## shifted. Consider using geom_tile() instead.
```

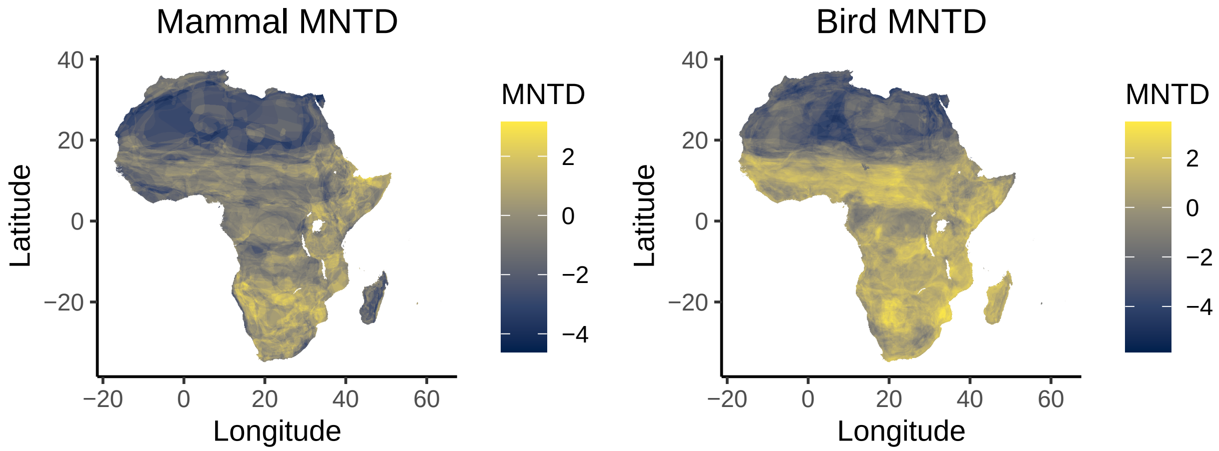


```
a=ggplot()+
  geom_raster(data=mntd.mamm.dat,
              aes(x=Var1,y=Var2,fill=mean.mntd.mamm))+
  labs(x="Longitude",y="Latitude",fill="MNTD")+
  scale_fill_viridis_c(option="cividis")+
```

```r
  theme_classic()+
  #theme(plot.title=element_text(hjust=0.5))+
  coord_quickmap()

b=ggplot()+
  geom_raster(data=mntd.bird.dat,
              aes(x=Var1,y=Var2,fill=mean.mntd.bird))+
  labs(x="Longitude",y="Latitude",fill="MNTD")+
  scale_fill_viridis_c(option="cividis")+
  theme_classic()+
  #theme(plot.title=element_text(hjust=0.5))+
  coord_quickmap()

ggsave(filename=paste0(filepath,"mntd_mamm.png"),a,dpi=400)
```

```
## Saving 6.5 x 4.5 in image

## Warning: Raster pixels are placed at uneven horizontal intervals and will be
## shifted. Consider using geom_tile() instead.
```

```r
ggsave(filename=paste0(filepath,"mntd_bird.png"),b,dpi=400)
```

```
## Saving 6.5 x 4.5 in image

## Warning: Raster pixels are placed at uneven horizontal intervals and will be
## shifted. Consider using geom_tile() instead.
```

Now we can rasterize these and compare them directly. First we have to normalize a vector x between zero and one by using the equation $\frac{x'-min(x')}{max(x')-min(x')}$.

```r
# standardize values

mntd.mamm.stand=mntd.mamm.dat
mntd.bird.stand=mntd.bird.dat

mntd.mamm.stand$mean.mntd.mamm=normalize(mntd.mamm.stand$mean.mntd.mamm)
mntd.bird.stand$mean.mntd.bird=normalize(mntd.bird.stand$mean.mntd.bird)

# randomize

mntd.mamm.random=randomize(mntd.mamm.stand,seed=81507)
mntd.bird.random=randomize(mntd.bird.stand,seed=62901)

# rasterize

mntd.mamm.rast=rasterFromXYZ(mntd.mamm.random)
mntd.bird.rast=rasterFromXYZ(mntd.bird.random)
```

```r
raster.compare(stack1 = mntd.mamm.rast,stack2 = mntd.bird.rast,
               metric="I",category = "Mean Nearest Taxon Distance")
```

```
## [1] "Test: Mammals vs. Random Birds: Mean Nearest Taxon Distance"
##
##  One Sample t-test
##
## data:  mamm.stats
## t = -4845.8, df = 99, p-value < 2.2e-16
```

```
## alternative hypothesis: true mean is not equal to 0.9923956
## 95 percent confidence interval:
##  0.8737611 0.8738582
## sample estimates:
## mean of x
## 0.8738096
##
## [1] "Test: 0.992395637641325"
## [1] "Test: Birds vs. Random MammalsMean Nearest Taxon Distance"
##
##  One Sample t-test
##
## data:  mamm.stats
## t = -4845.8, df = 99, p-value < 2.2e-16
## alternative hypothesis: true mean is not equal to 0.9923956
## 95 percent confidence interval:
##  0.8737611 0.8738582
## sample estimates:
## mean of x
## 0.8738096
##
## [1] "Test: 0.992395637641325"
## [1] "Visualization:"
```

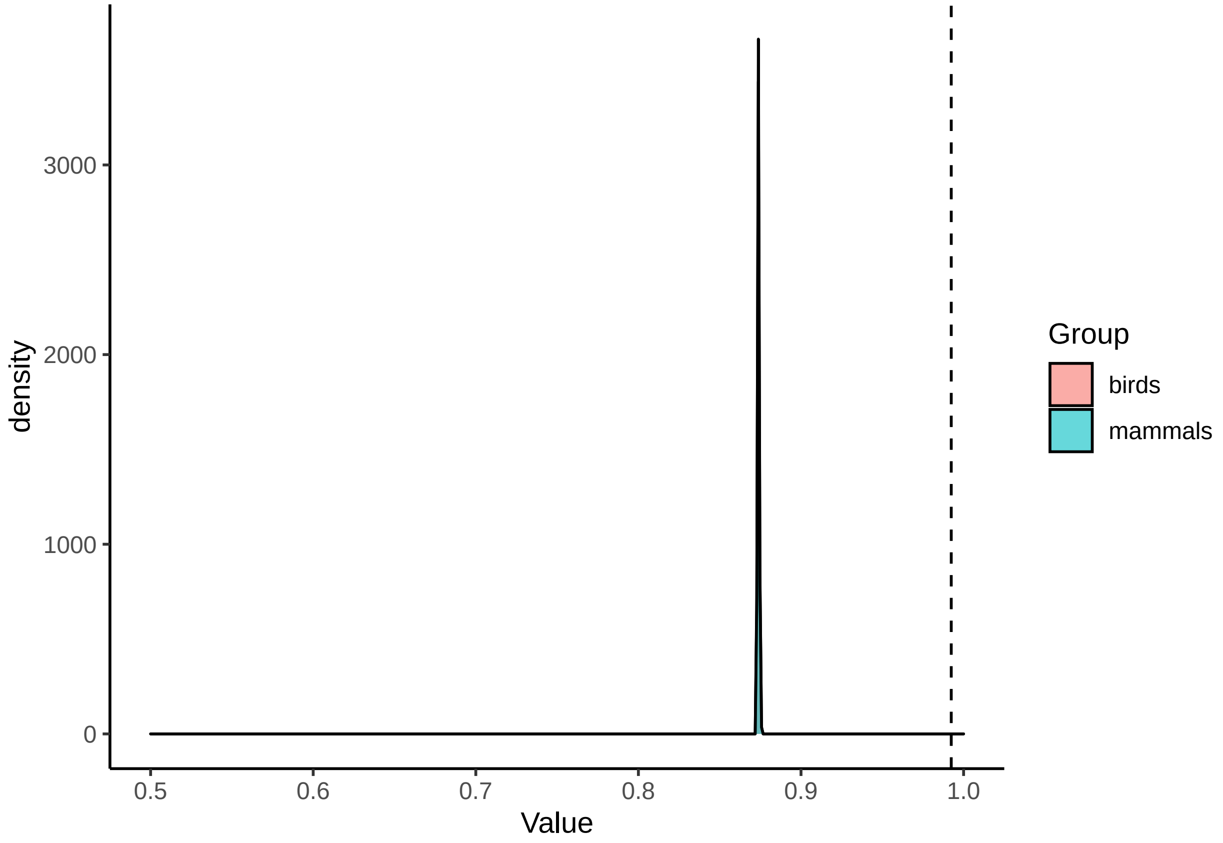


```
raster.compare(stack1 = mntd.mamm.rast,stack2 = mntd.bird.rast,
               metric="D",category = "Mean Nearest Taxon Distance")
```

```
## [1] "Test: Mammals vs. Random Birds: Mean Nearest Taxon Distance"
##
##  One Sample t-test
##
## data:  mamm.stats
## t = -6299.3, df = 99, p-value < 2.2e-16
## alternative hypothesis: true mean is not equal to 0.9065873
## 95 percent confidence interval:
##  0.6314348 0.6316080
## sample estimates:
## mean of x
## 0.6315214
##
## [1] "Test: 0.906587331161116"
## [1] "Test: Birds vs. Random MammalsMean Nearest Taxon Distance"
##
##  One Sample t-test
##
## data:  mamm.stats
## t = -6299.3, df = 99, p-value < 2.2e-16
## alternative hypothesis: true mean is not equal to 0.9065873
## 95 percent confidence interval:
##  0.6314348 0.6316080
## sample estimates:
## mean of x
## 0.6315214
##
## [1] "Test: 0.906587331161116"
## [1] "Visualization:"
```

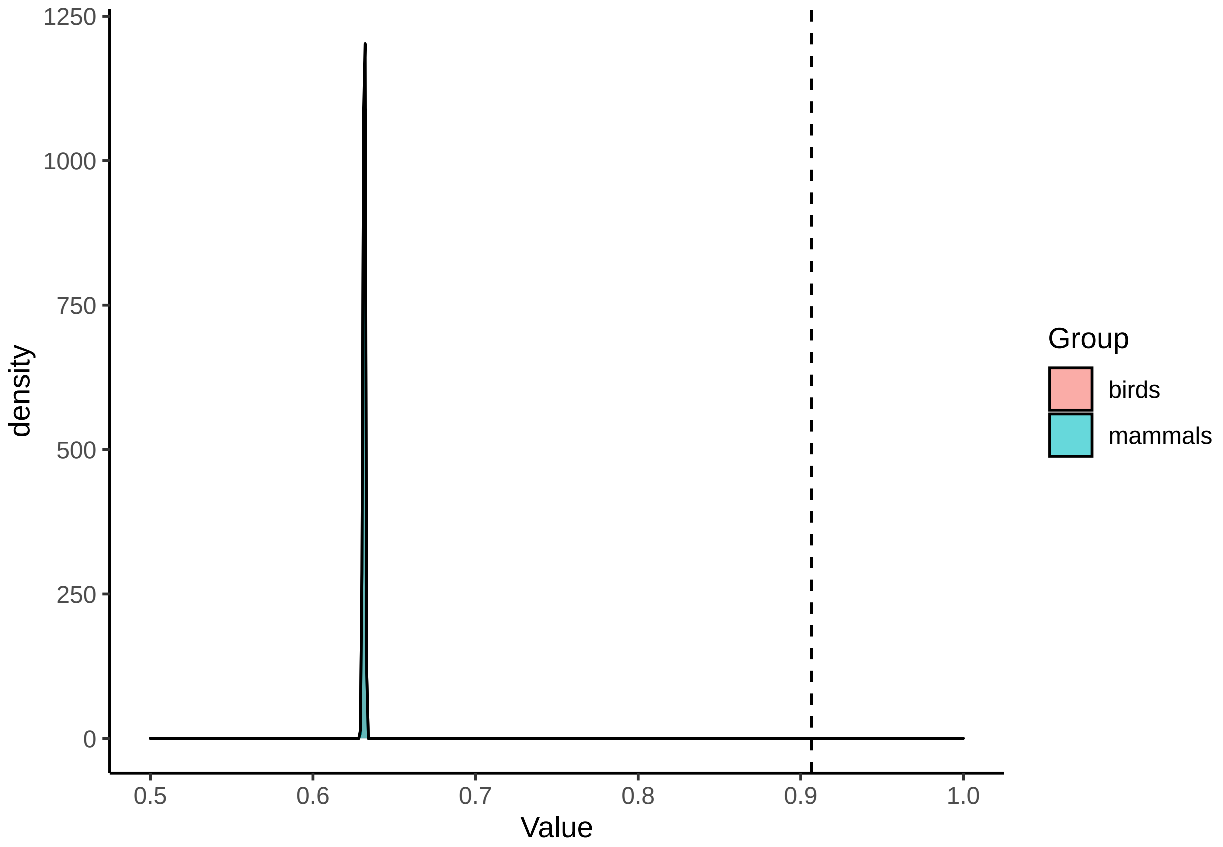

These distributions are overall quite similar still, when compared using these raster metrics.

```
comparedf=cbind(mntd.mamm.dat,mntd.bird.dat$mean.mntd.bird)%>%as.data.frame()

colnames(comparedf)=c("Long","Lat","Mamm","Bird")

df.lm=lm(Bird~Mamm,comparedf)

summary(df.lm)
```

```
##
## Call:
## lm(formula = Bird ~ Mamm, data = comparedf)
##
## Residuals:
##     Min      1Q  Median      3Q     Max
## -6.3483 -0.8839 -0.0082  0.9015  4.2865
##
## Coefficients:
##             Estimate Std. Error t value Pr(>|t|)
## (Intercept) 0.513417   0.003002     171   <2e-16 ***
## Mamm        0.985297   0.002040     483   <2e-16 ***
## ---
## Signif. codes:  0 '***' 0.001 '**' 0.01 '*' 0.05 '.' 0.1 ' ' 1
##
## Residual standard error: 1.273 on 254359 degrees of freedom
## Multiple R-squared:  0.4784, Adjusted R-squared:  0.4784
```

```
## F-statistic: 2.333e+05 on 1 and 254359 DF,  p-value: < 2.2e-16
```

```
cor(comparedf[,-c(1:2)])
```

```
##          Mamm     Bird
## Mamm 1.000000 0.691641
## Bird 0.691641 1.000000
```

```
a=ggplot(comparedf,aes(x=Mamm,y=Bird))+
  geom_point(size=.05)+
  geom_smooth(method="lm",formula=y~x)+
  theme_classic()

a
```

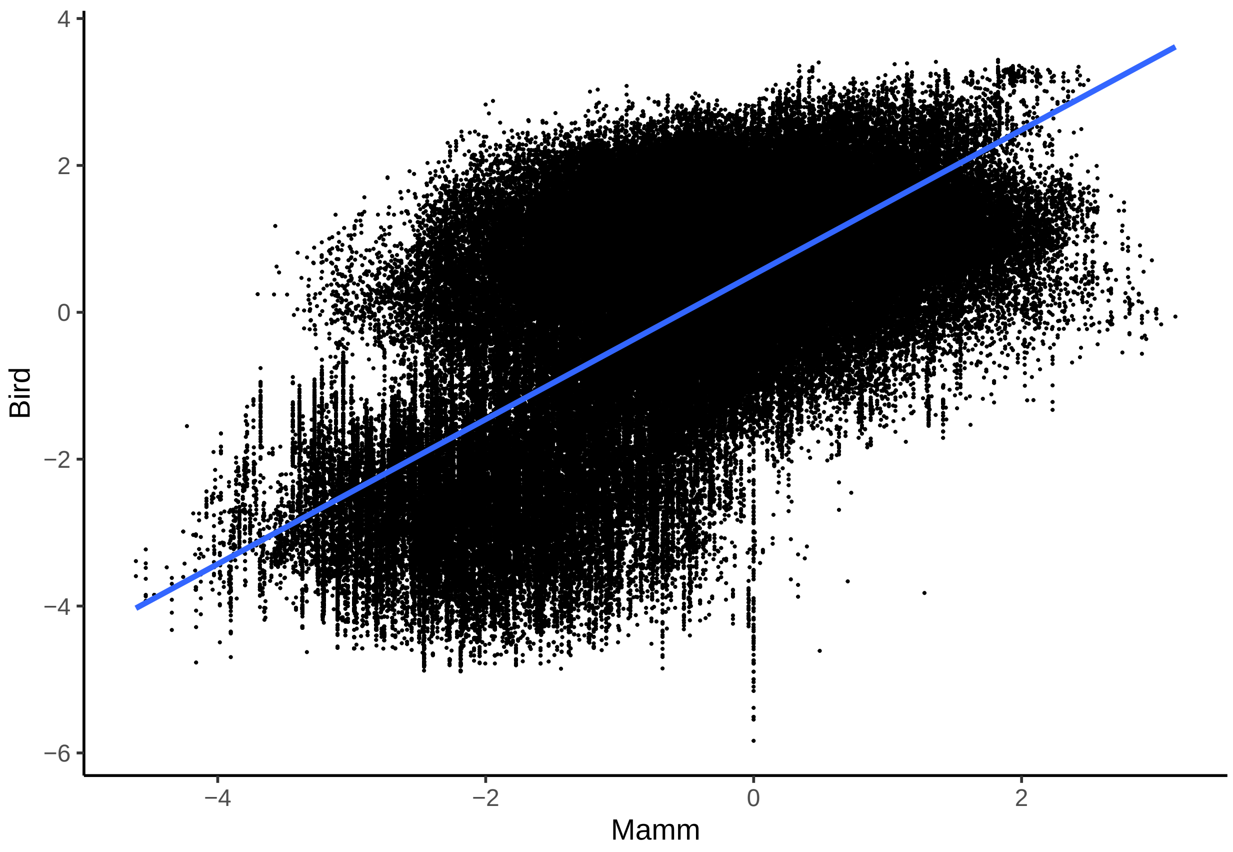

Range Calculations Range calculations.

```
range.mamm.mntd=apply(mntd.mamm,1,range.diff)
range.bird.mntd=apply(mntd.bird,1,range.diff)
```

```
## Rows: 254361 Columns: 2
```

```
## -- Column specification --------------------------------------------------------
## Delimiter: ","
## dbl (2): range.mamm.mntd, range.bird.mntd
```

```
##
## i Use `spec()` to retrieve the full column specification for this data.
## i Specify the column types or set `show_col_types = FALSE` to quiet this message.
```

```r
mpd.mamm.dat2=cbind(coords,range.mamm.mntd)
mpd.bird.dat2=cbind(coords,range.bird.mntd)
```

```r
a=ggplot()+
  geom_raster(data=mpd.mamm.dat2,
              aes(x=Var1,y=Var2,fill=range.mamm.mntd))+
  labs(x="Longitude",y="Latitude",fill="MPD",title="Mammal MPD Range")+
  scale_fill_viridis_c(option="cividis")+
  theme_classic()+
  theme(plot.title=element_text(hjust=0.5))+
  coord_quickmap()

b=ggplot()+
  geom_raster(data=mpd.bird.dat2,
              aes(x=Var1,y=Var2,fill=range.bird.mntd))+
  labs(x="Longitude",y="Latitude",fill="MPD",title="Bird MPD Range")+
  scale_fill_viridis_c(option="cividis")+
  theme_classic()+
  theme(plot.title=element_text(hjust=0.5))+
  coord_quickmap()
```

```r
grid.arrange(a,b,ncol=2)
```

```
## Warning: Raster pixels are placed at uneven horizontal intervals and will be
## shifted. Consider using geom_tile() instead.

## Warning: Raster pixels are placed at uneven horizontal intervals and will be
## shifted. Consider using geom_tile() instead.
```

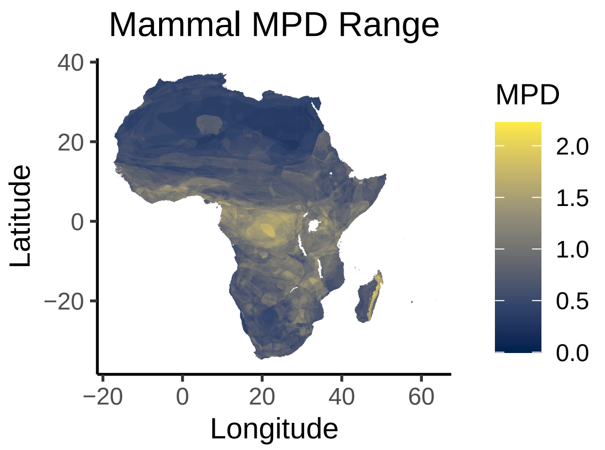

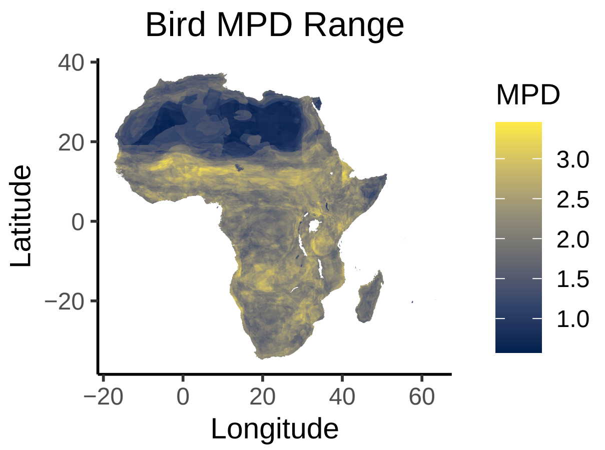

3 Over and Under Dispersion Metrics

```
source(paste0(filepath,"under.over.dispersion.R"))
```

We are going to perform the `over.under.dispersion` code manually. This has to be partially run on the cluster due to memory requirements. Note that we load the phylogenetic trees here, but the codes are hidden.

```
pdq.df.mamm=read_csv(paste0(filepath,"mammal_pd_query.csv"))
pdq.df.bird=read_csv(paste0(filepath,"bird_pd_query.csv"))

mean.pdq.mamm <- apply(pdq.df.mamm, 1, mean)
mean.pdq.bird <- apply(pdq.df.bird, 1, mean)
```

```
## Rows: 254361 Columns: 2

## -- Column specification --------------------------------------------------------
## Delimiter: ","
## dbl (2): mean.pdq.mamm, mean.pdq.bird

##
## i Use `spec()` to retrieve the full column specification for this data.
## i Specify the column types or set `show_col_types = FALSE` to quiet this message.
```

```
mamm.lin.mod=lm(mean.pdq.mamm~spdv.mamm)

mamm.modelConfInt=predict(mamm.lin.mod,
                          level=0.95,
```

```
                          interval="confidence")

mamm.insideInterval=mamm.modelConfInt[,'lwr']<mean.pdq.mamm&
  mean.pdq.mamm<mamm.modelConfInt[,'upr']

mamm.aboveInterval=mean.pdq.mamm>mamm.modelConfInt[,'upr']

colors=viridis(3,option="plasma")

mamm.plot.cols=rep(colors[1],length(spdv.mamm))
mamm.plot.cols[mamm.insideInterval==TRUE] <- colors[2]
mamm.plot.cols[mamm.aboveInterval==TRUE] <- colors[3]

mamm.res.df <- data.frame(res = rep(0, length(spdv.mamm)))
mamm.res.df$res[mamm.insideInterval==TRUE] <- 1
mamm.res.df$res[mamm.aboveInterval==TRUE] <- 2

mamm.all.res.df <- cbind(coords, mamm.res.df)

mamm.num.0=grep(0,mamm.res.df$res)%>%length
mamm.num.1=grep(1,mamm.res.df$res)%>%length
mamm.num.2=grep(2,mamm.res.df$res)%>%length

mamm.number.res=c(mamm.num.0,mamm.num.1,mamm.num.2)
names(mamm.number.res)=c("Points Below",
                         "Points Inside",
                         "Points Above")

mamm.coords=coords
mamm.coords$val=mamm.res.df$res

coordinates(mamm.coords)=colnames(coords)[1:2]
gridded(mamm.coords)=T
```

```
## Warning in points2grid(points, tolerance, round): grid has empty column/rows in
## dimension 1
```

```
mamm.res.sgdf=as(mamm.coords,"SpatialGridDataFrame")

plot(mamm.res.sgdf,col=colors)
```

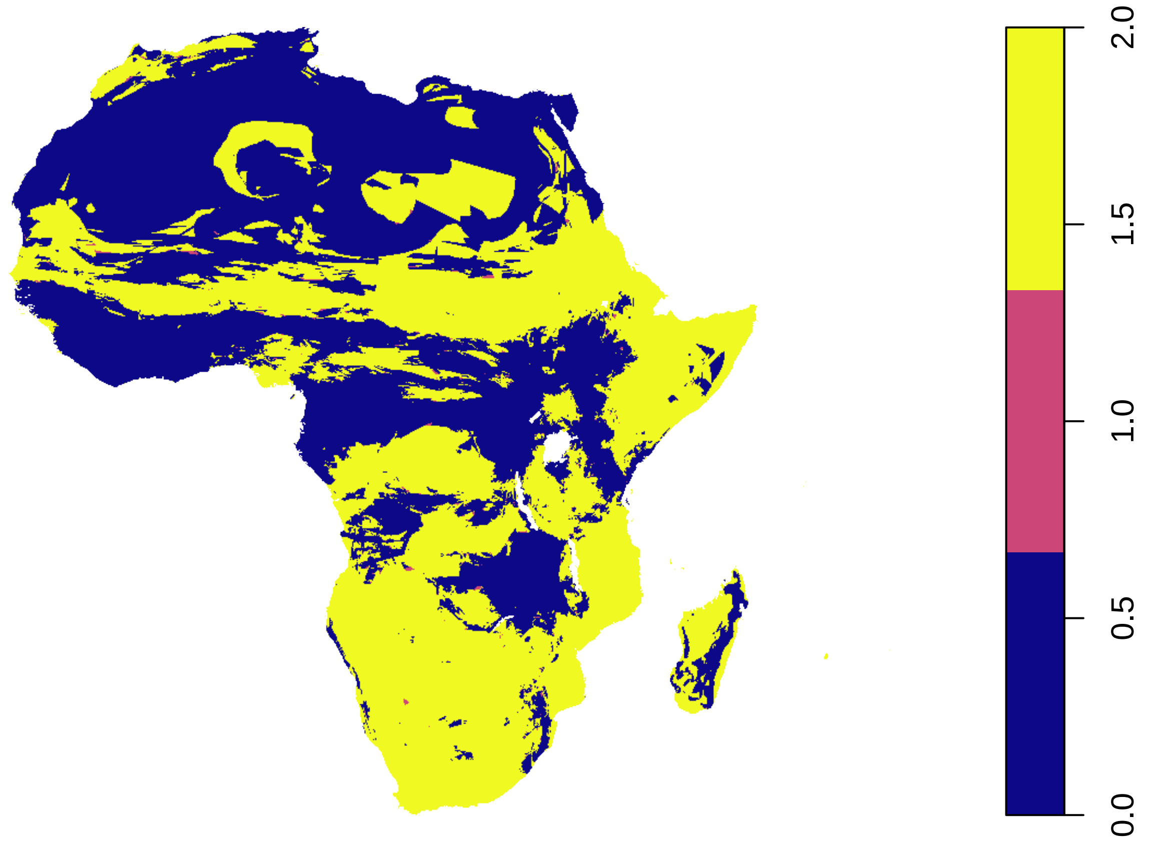


```
mamm.output=vector(mode="list",length=3)
names(mamm.output)=c("Point Count",
                     "Data.Frame Results",
                     "Spatial Grid Data.Frame Results")

mamm.output[[1]]=mamm.number.res
mamm.output[[2]]=mamm.all.res.df
mamm.output[[3]]=mamm.res.sgdf
#print(mamm.output)
```

```
print(mamm.number.res)
```

```
##  Points Below Points Inside  Points Above
##        128762           797        124802
```

```
bird.lin.mod=lm(mean.pdq.bird~spdv.bird)

bird.modelConfInt=predict(bird.lin.mod,
                          level=0.95,
                          interval="confidence")

bird.insideInterval=bird.modelConfInt[,'lwr']<mean.pdq.bird&
  mean.pdq.bird<bird.modelConfInt[,'upr']

bird.aboveInterval=mean.pdq.bird>bird.modelConfInt[,'upr']

colors=viridis(3,option="plasma")
```

```r
bird.plot.cols=rep(colors[1],length(spdv.bird))
bird.plot.cols[bird.insideInterval==TRUE] <- colors[2]
bird.plot.cols[bird.aboveInterval==TRUE] <- colors[3]

bird.res.df <- data.frame(res = rep(0, length(spdv.bird)))
bird.res.df$res[bird.insideInterval==TRUE] <- 1
bird.res.df$res[bird.aboveInterval==TRUE] <- 2

bird.all.res.df <- cbind(coords, bird.res.df)

bird.num.0=grep(0,bird.res.df$res)%>%length
bird.num.1=grep(1,bird.res.df$res)%>%length
bird.num.2=grep(2,bird.res.df$res)%>%length

bird.number.res=c(bird.num.0,bird.num.1,bird.num.2)
names(bird.number.res)=c("Points Below",
                         "Points Inside",
                         "Points Above")

bird.coords=coords
bird.coords$val=bird.res.df$res

coordinates(bird.coords)=colnames(coords)[1:2]
gridded(bird.coords)=T
```

```
## Warning in points2grid(points, tolerance, round): grid has empty column/rows in
## dimension 1
```

```r
bird.res.sgdf=as(bird.coords,"SpatialGridDataFrame")

plot(bird.res.sgdf,col=colors)
```

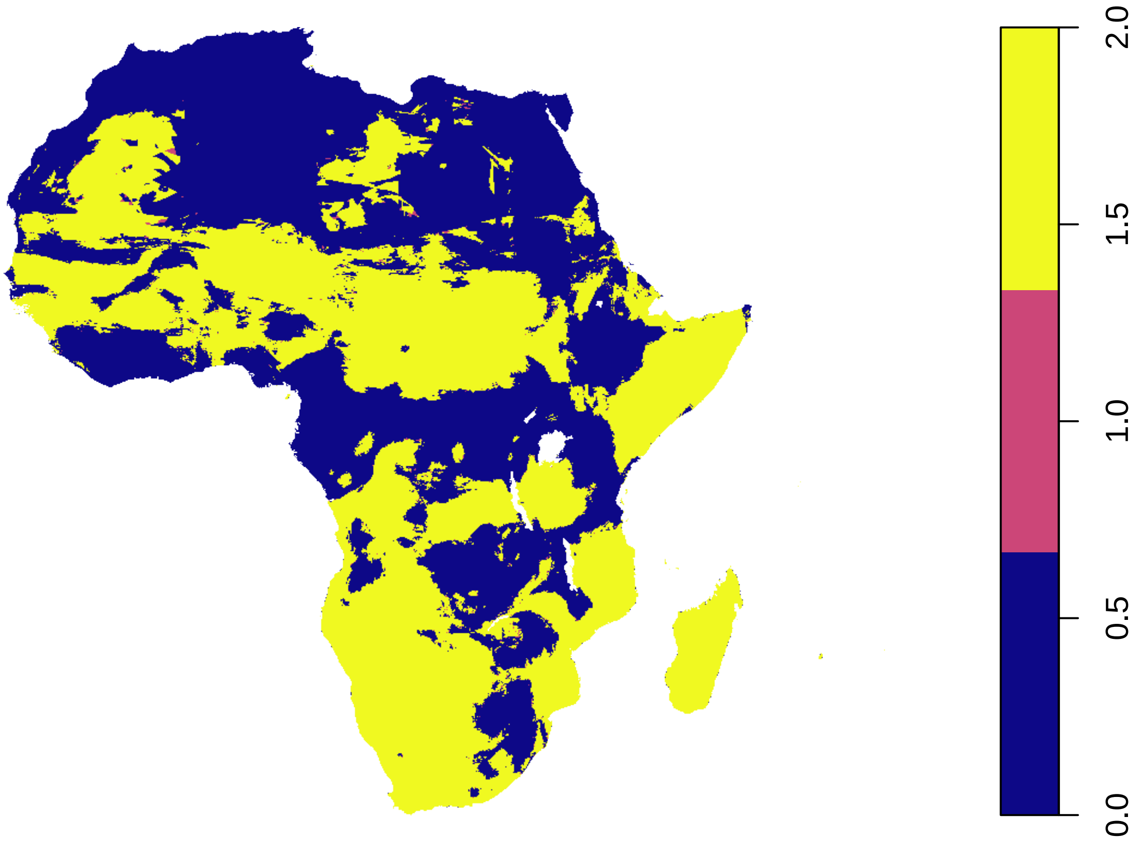


```
bird.output=vector(mode="list",length=3)
names(bird.output)=c("Point Count",
                     "Data.Frame Results",
                     "Spatial Grid Data.Frame Results")

bird.output[[1]]=bird.number.res
bird.output[[2]]=bird.all.res.df
bird.output[[3]]=bird.res.sgdf
#print(bird.output)
```

```
print(bird.number.res)
```

```
##  Points Below Points Inside  Points Above
##        127382          1136        125843
```

Now we can compare the rasters of these outputs as well.

```
pdq.mamm.rast=raster(mamm.res.sgdf)
pdq.bird.rast=raster(bird.res.sgdf)

# Richness raster similarity, I

nicheOverlap(pdq.mamm.rast,pdq.bird.rast,stat="I")
```

```
## [1] 0.649825
```

```
# Richness raster similarity, I
```

```r
nicheOverlap(pdq.mamm.rast,pdq.bird.rast,stat="D")
```

```
## [1] 0.6452045
```

We can also look at niche overlap for the phylogenetic diversity measures themselves.

```r
# normalize values

pdq.mamm.stand=cbind(coords,mean.pdq.mamm)%>%as.data.frame()
pdq.bird.stand=cbind(coords,mean.pdq.bird)%>%as.data.frame()

pdq.mamm.stand$mean.pdq.mamm=normalize(pdq.mamm.stand$mean.pdq.mamm)
pdq.bird.stand$mean.pdq.bird=normalize(pdq.bird.stand$mean.pdq.bird)

# randomize

pdq.mamm.random=randomize(pdq.mamm.stand,seed=81507)
pdq.bird.random=randomize(pdq.bird.stand,seed=62901)

# rasterize

pdq.mamm.rast=rasterFromXYZ(pdq.mamm.random)
pdq.bird.rast=rasterFromXYZ(pdq.bird.random)
```

```r
raster.compare(stack1 = pdq.mamm.rast,stack2 = pdq.bird.rast,
               metric="I",category = "Dispersion")
```

```
## [1] "Test: Mammals vs. Random Birds: Dispersion"
##
##  One Sample t-test
##
## data:  mamm.stats
## t = -4845.8, df = 99, p-value < 2.2e-16
## alternative hypothesis: true mean is not equal to 0.9923956
## 95 percent confidence interval:
##  0.8737611 0.8738582
## sample estimates:
## mean of x
## 0.8738096
##
## [1] "Test: 0.992395637641325"
## [1] "Test: Birds vs. Random MammalsDispersion"
##
##  One Sample t-test
##
## data:  mamm.stats
## t = -4845.8, df = 99, p-value < 2.2e-16
## alternative hypothesis: true mean is not equal to 0.9923956
## 95 percent confidence interval:
##  0.8737611 0.8738582
## sample estimates:
## mean of x
## 0.8738096
##
## [1] "Test: 0.992395637641325"
```

```
## [1] "Visualization:"
```

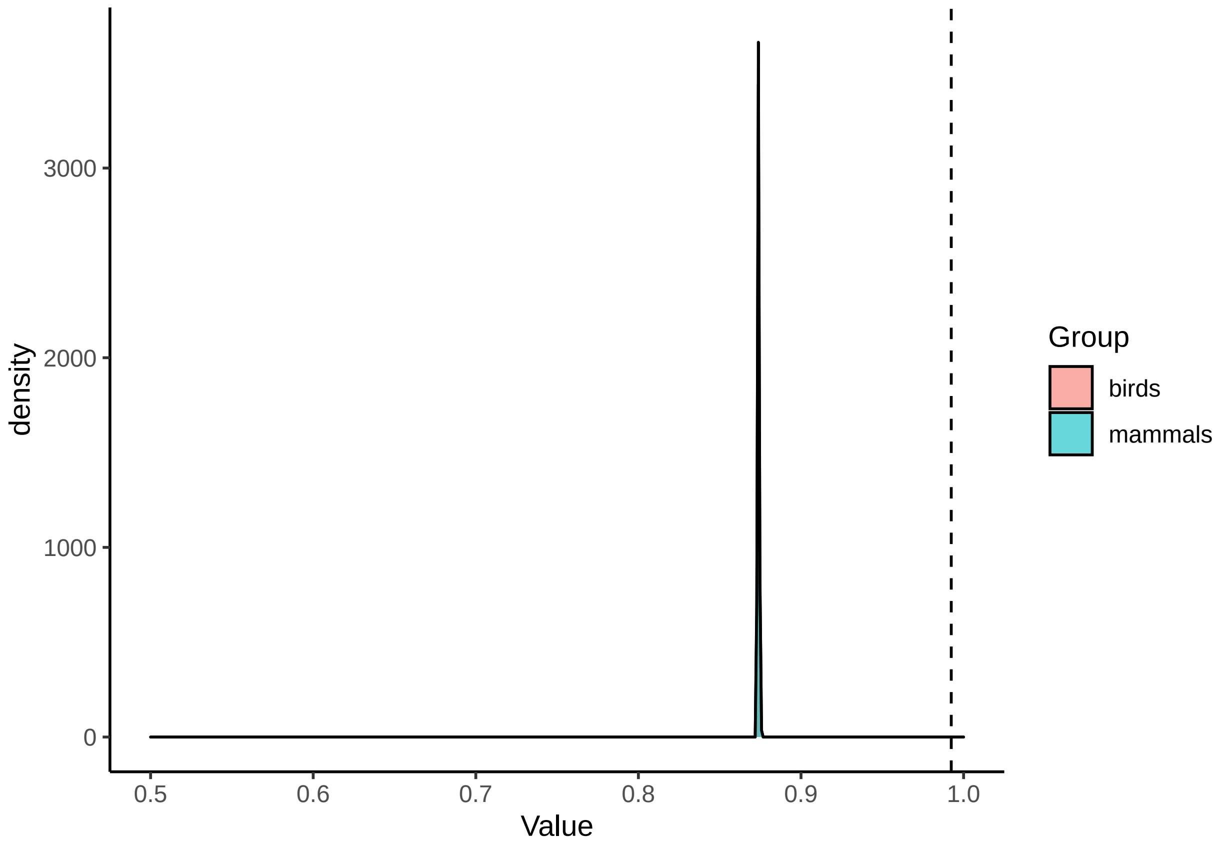


```r
raster.compare(stack1 = pdq.mamm.rast,stack2 = pdq.bird.rast,
               metric="D",category = "Dispersion")
```

```
## [1] "Test: Mammals vs. Random Birds: Dispersion"
##
##  One Sample t-test
##
## data:  mamm.stats
## t = -6299.3, df = 99, p-value < 2.2e-16
## alternative hypothesis: true mean is not equal to 0.9065873
## 95 percent confidence interval:
##  0.6314348 0.6316080
## sample estimates:
## mean of x
## 0.6315214
##
## [1] "Test: 0.906587331161116"
## [1] "Test: Birds vs. Random MammalsDispersion"
##
##  One Sample t-test
##
## data:  mamm.stats
## t = -6299.3, df = 99, p-value < 2.2e-16
## alternative hypothesis: true mean is not equal to 0.9065873
## 95 percent confidence interval:
```

```
##  0.6314348 0.6316080
## sample estimates:
## mean of x
## 0.6315214
##
## [1] "Test: 0.906587331161116"
## [1] "Visualization:"
```

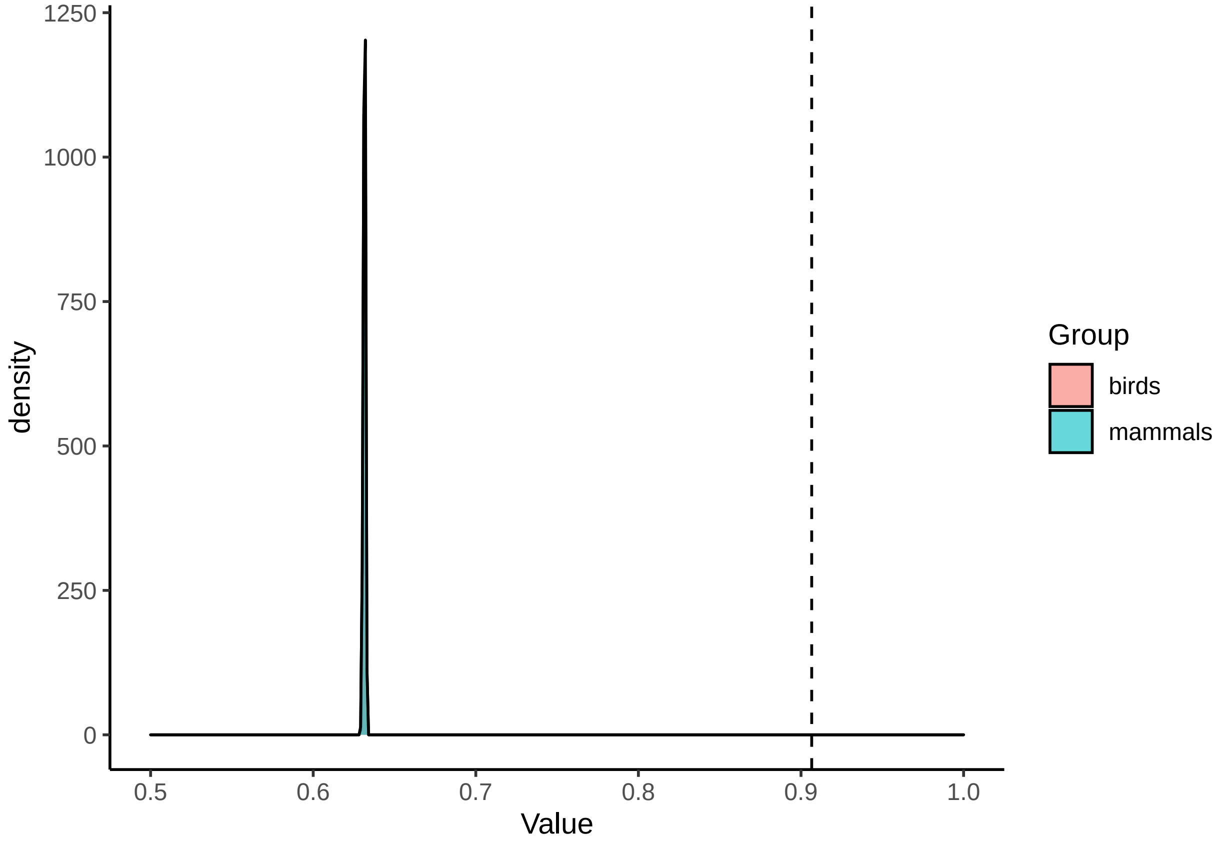


```
comparedf=cbind(mamm.all.res.df,bird.all.res.df$res)%>%as.data.frame()

colnames(comparedf)=c("Long","Lat","Mamm","Bird")

df.lm=lm(Bird~Mamm,comparedf)

summary(df.lm)
```

Comparing dispersion between groups

```
##
## Call:
## lm(formula = Bird ~ Mamm, data = comparedf)
##
## Residuals:
##     Min      1Q  Median      3Q     Max
## -1.3038 -0.6936 -0.6936  0.6962  1.3064
##
```

```
## Coefficients:
##             Estimate Std. Error t value Pr(>|t|)
## (Intercept) 0.693574   0.002646   262.1   <2e-16 ***
## Mamm        0.305126   0.001887   161.7   <2e-16 ***
## ---
## Signif. codes:  0 '***' 0.001 '**' 0.01 '*' 0.05 '.' 0.1 ' ' 1
##
## Residual standard error: 0.9501 on 254359 degrees of freedom
## Multiple R-squared:  0.09321,    Adjusted R-squared:  0.0932
## F-statistic: 2.614e+04 on 1 and 254359 DF,  p-value: < 2.2e-16
```

```
cor(comparedf[,-c(1:2)])
```

```
##           Mamm      Bird
## Mamm 1.0000000 0.3052982
## Bird 0.3052982 1.0000000
```

```
table(comparedf[,-c(1:2)])
```

```
##     Bird
## Mamm     0     1     2
##    0 83805   612 44345
##    1   395     0   402
##    2 43182   524 81096
```

We can also do a chi squared (χ^2) test of similarity between these categories.

```
print(chisq.test(comparedf$Mamm,comparedf$Bird))
```

```
## Warning in chisq.test(comparedf$Mamm, comparedf$Bird): Chi-squared approximation
## may be incorrect
```

```
##
##  Pearson's Chi-squared test
##
## data:  comparedf$Mamm and comparedf$Bird
## X-squared = 23717, df = 4, p-value < 2.2e-16
```

4 Ecoregion Mapping

Load the African ecoregion shapefile.

```
ecos=readOGR(paste0(shp.path,"wwf_terr_ecos.shp"))
```

```
plot(ecos)
```

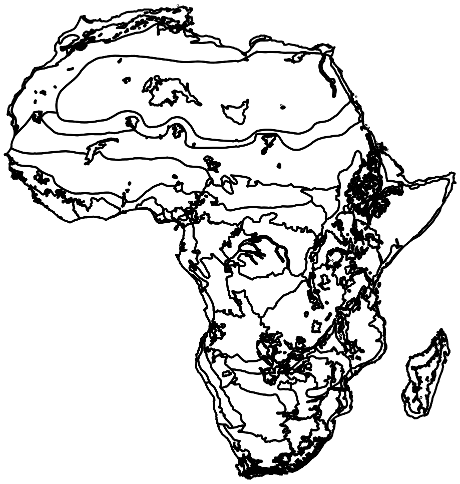

```
# prepare for subsetting

ecos.names=unique(ecos$ECO_NAME)
```

Now, we can load MPD and MNTD data by ecoregion.

```
colnames(coords)=c("Long","Lat")

mntd=read_csv(paste0(filepath,'mean_mntd.csv'))

mpd=read_csv(paste0(filepath,"mean_mpd.csv"))

pdq=read_csv(paste0(filepath,"mean_pdq.csv"))

df=cbind(coords,mntd,mpd,pdq)

# identify rows for each ecoregion
# save as a new file
# limited to mainland Africa/Madagascar to exclude oceanic islands

x.coords=SpatialPoints(coords)
crs(x.coords)=crs(ecos)
overdf=over(x.coords,ecos)
overdf2=overdf%>%dplyr::select(ECO_NAME,G200_REGIO)
df2=cbind(df,overdf2)
```

```
## Rows: 254361 Columns: 10
```

```
## -- Column specification --------------------------------------------------------
## Delimiter: ","
## chr (2): ECO_NAME, G200_REGIO
## dbl (8): Long, Lat, mean.mntd.mamm, mean.mntd.bird, mean.mpd.mamm, mean.mpd....
```

```
##
## i Use `spec()` to retrieve the full column specification for this data.
## i Specify the column types or set `show_col_types = FALSE` to quiet this message.
```

First, we can see which points are not assigned to region in this assessment.

```
na.x=which(is.na(df2$ECO_NAME))
plot(x=df2$Long,y=df2$Lat,pch=".",col="black",asp=1)
points(x=df2$Long[na.x],y=df2$Lat[na.x],pch=".",col="red")
```

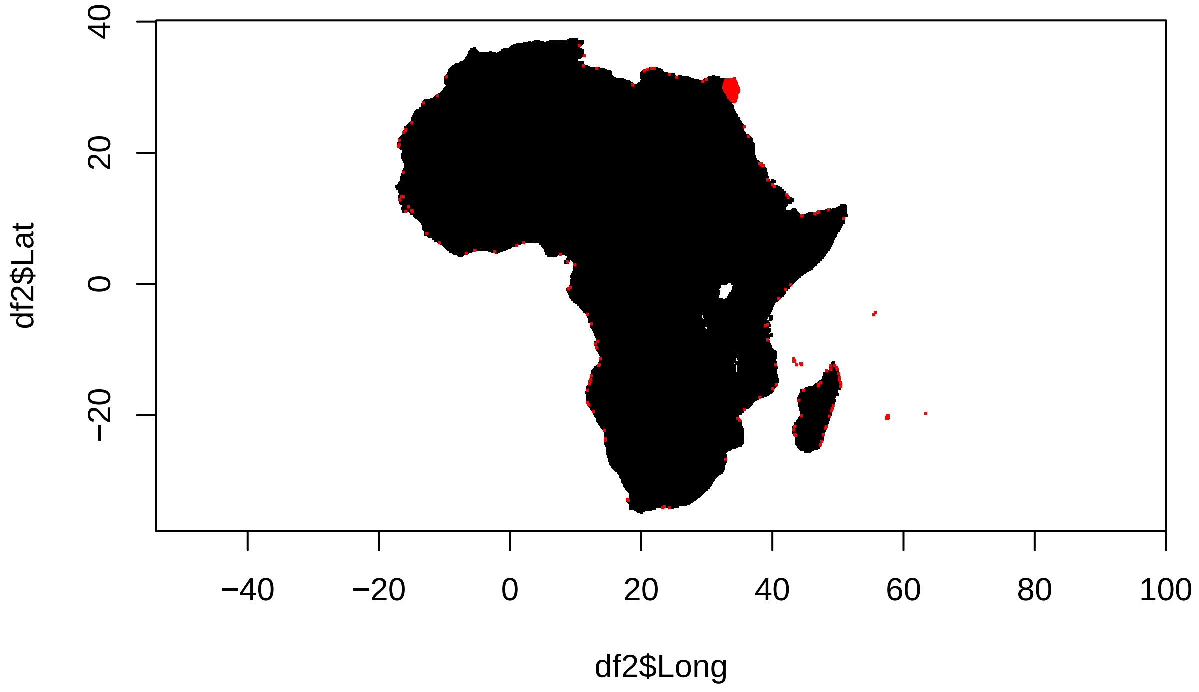

NA areas are along coasts, the very edges of water bodies, and small islands/ancillary areas that were removed.

Now, we can compare distributions of different regions and different ecological areas.

First, we need to create long vectors of mammals and birds for easier comparisons in the same plots.

```
mamm=df2%>%dplyr::select(Long,Lat,
                         mean.mntd.mamm,
                         mean.mpd.mamm,
                         mean.pdq.mamm,
                         ECO_NAME,G200_REGIO)
bird=df2%>%dplyr::select(Long,Lat,
                         mean.mntd.bird,
```

```
                           mean.mpd.bird,
                           mean.pdq.bird,
                           ECO_NAME,
                           G200_REGIO)

mamm=cbind("Mammals",mamm)
bird=cbind("Birds",bird)

colnames(mamm)=colnames(bird)=c("Group","Long","Lat",
                                "MNTD","MPD","PDQ","Ecoregion","Region")

bx.df=rbind(mamm,bird)

bx.df=bx.df[-which(is.na(bx.df$Ecoregion)),]
```

```
a=ggplot(bx.df,aes(x=Region,y=MNTD,fill=Group))
b=geom_boxplot(notch=T,outlier.shape=".",outlier.colour = "red")
c=coord_flip()
d=theme_classic()

a+b+c+d
```

```
## notch went outside hinges. Try setting notch=FALSE.
```

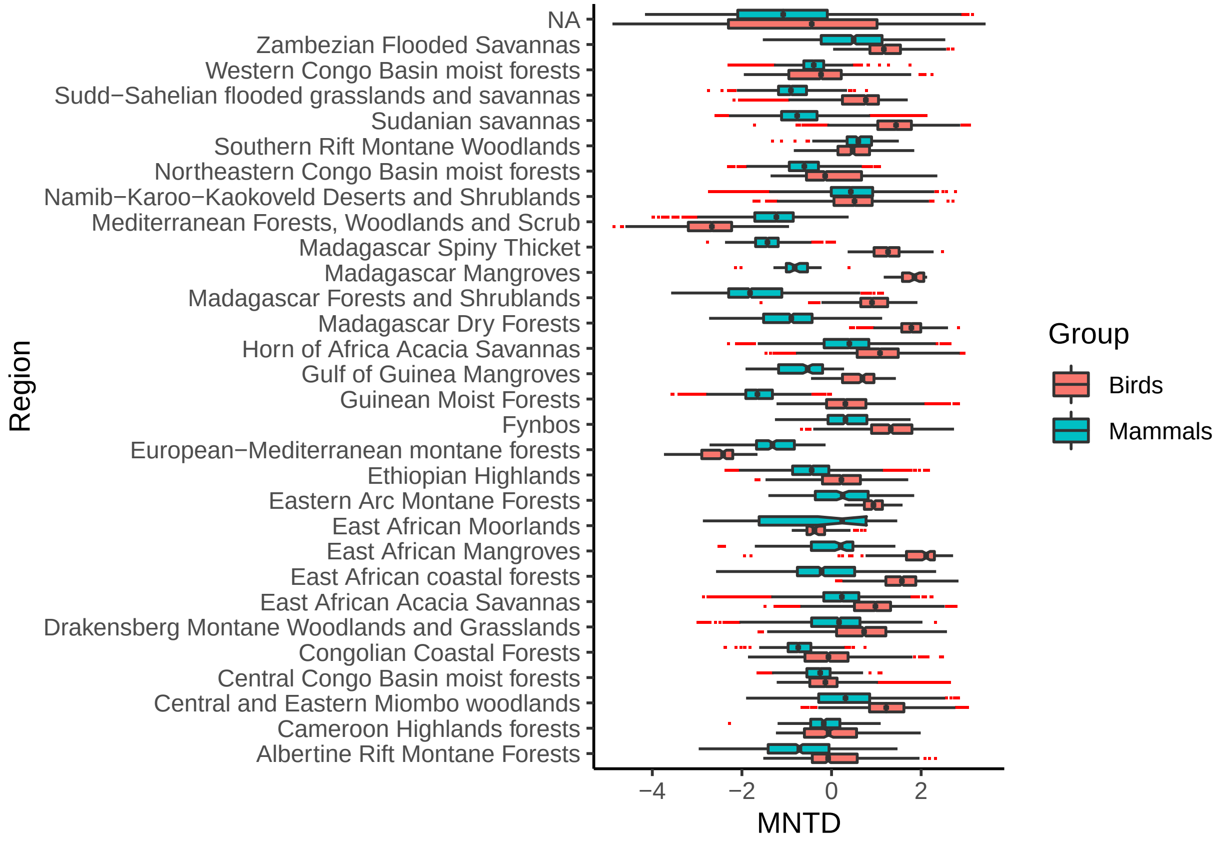

We can see a lot of discrepancies for MNTd between regions, though there are some similarities. This is essentially what is being told to us by the regressions as well, except corrected for the ecoregions. Combinations of this can be performed for other variables and groups as well; however, these are not largely informative /

do not show patterns that have not already been described or are not discernible above.

We can move on to the visualization in geographic space for dispersion.

```
mamm.coords=coords
mamm.coords$o.u.val=mamm.res.df$res
bird.coords=coords
bird.coords$o.u.val=bird.res.df$res

colnames(mamm.coords)=colnames(bird.coords)=c("Long","Lat","o.u.val")

bx.df.mamm=bx.df%>%filter(Group=="Mammals")%>%
  inner_join(mamm.coords,by=c('Long','Lat'))
bx.df2=bx.df%>%filter(Group=="Birds")%>%
  inner_join(bird.coords,by=c("Long","Lat"))%>%
  rbind(bx.df.mamm)
```

```
ecoregion.means.bird=NULL
ecoregion.means.mamm=NULL

df.x=bx.df2
df.x$Ecoregion=as.character(df.x$Ecoregion)
df.x$Region=as.character(df.x$Region)
ecoregions=unique(df.x$Ecoregion)

for(i in 1:length(ecoregions)){
  df.x2=df.x[which(df.x$Ecoregion==ecoregions[i]),]
  sub.bird=df.x2%>%filter(Group=="Birds")
  sub.mamm=df.x2%>%filter(Group=="Mammals")
  ecoregion.means.bird[i]=mean(sub.bird$o.u.val)
  ecoregion.means.mamm[i]=mean(sub.mamm$o.u.val)
}

d1=cbind("Birds",ecoregions,ecoregion.means.bird)
d2=cbind("Mammals",ecoregions,ecoregion.means.mamm)

colnames(d1)=colnames(d2)=c("Group","Ecoregion","OU_Avg")

d3=as.data.frame(rbind(d1,d2))
d3$OU_Avg=as.numeric(d3$OU_Avg)

df.x=df.x%>%inner_join(d3,by=c("Group","Ecoregion"))
```

```
b.df=df.x%>%filter(Group=="Birds")

vals=b.df$OU_Avg

cols=colourvalues::colour_values(vals)

cols.max=cols[which(vals==max(vals))[1]]
cols.min=cols[which(vals==min(vals))[1]]

plot(b.df$Long,b.df$Lat,asp=1,col=cols,pch=".",
     xlab="Longitude",ylab="Latitude",main="Bird Dispersion")
legend("bottomright",legend=c("Underdispersed","Overdispersed"),
       fill=c(cols.min,cols.max),border="black")
```

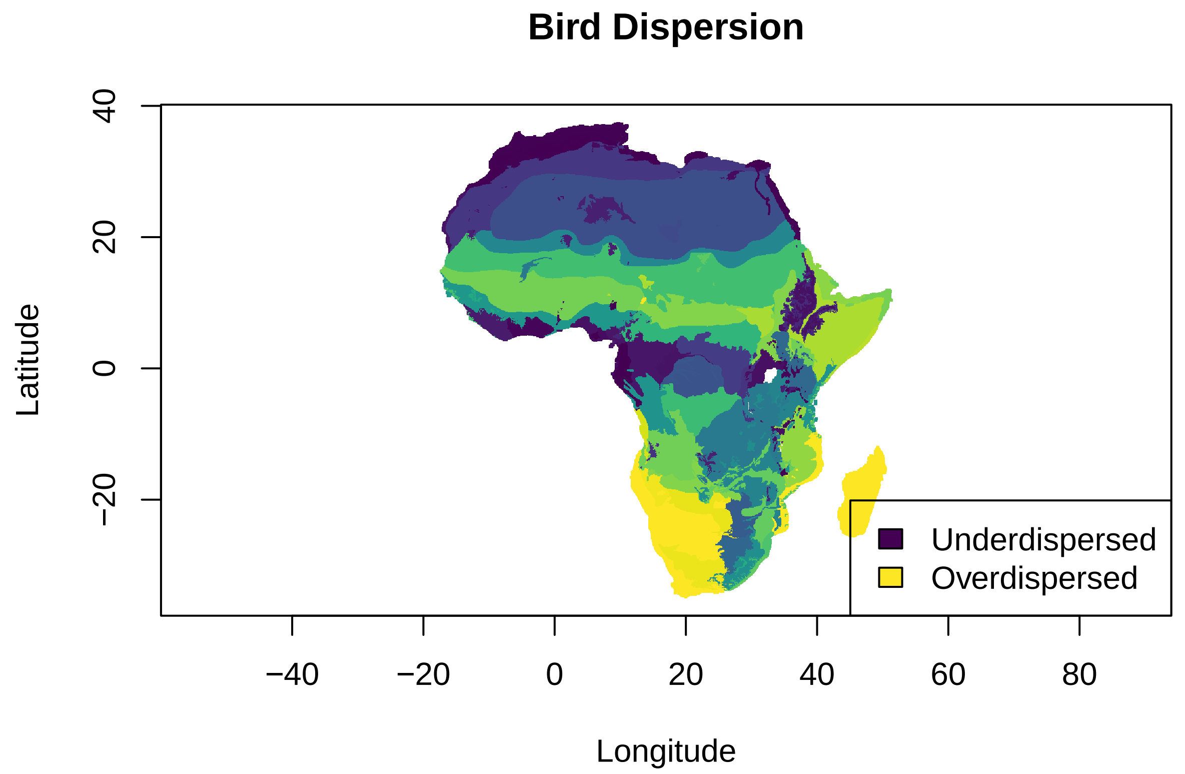


```
# plot and add legend
jpeg("bird_dispersion_eco.jpeg",quality=100)
plot(b.df$Long,b.df$Lat,asp=1,col=cols,pch=".",
     xlab="Longitude",ylab="Latitude",main="Bird Dispersion")
legend("bottomright",legend=c("Underdispersed","Overdispersed"),
       fill=c(cols.min,cols.max),border="black")
dev.off()
```

```
b.df=df.x%>%filter(Group=="Mammals")

vals=b.df$OU_Avg

cols=colourvalues::colour_values(vals)

cols.max=cols[which(vals==max(vals))[1]]
cols.min=cols[which(vals==min(vals))[1]]

plot(b.df$Long,b.df$Lat,asp=1,col=cols,pch=".",
     xlab="Longitude",ylab="Latitude",main="Mammal Dispersion")
legend("bottomright",legend=c("Underdispersed","Overdispersed"),
       fill=c(cols.min,cols.max),border="black")
```

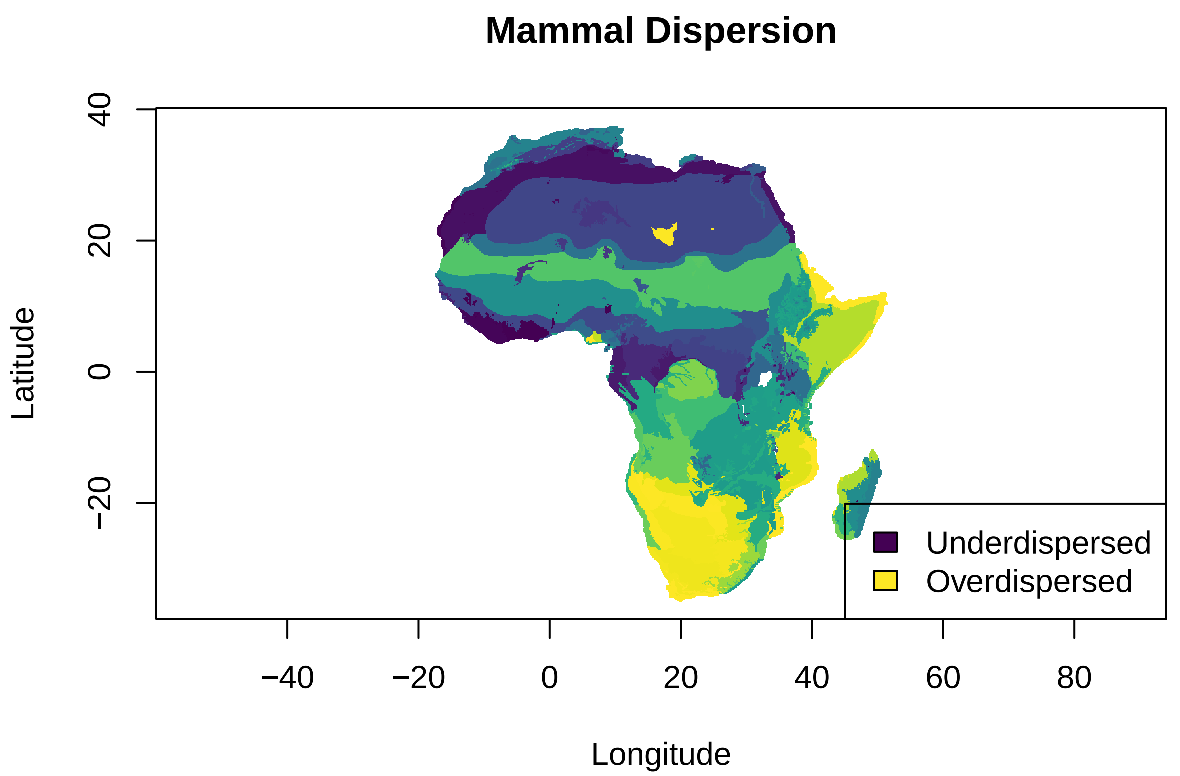


```
# plot and add legend
jpeg("mammal_dispersion_eco.jpeg",quality=100)
plot(b.df$Long,b.df$Lat,asp=1,col=cols,pch=".",
     xlab="Longitude",ylab="Latitude",main="Mammal Dispersion")
legend("bottomright",legend=c("Underdispersed","Overdispersed"),
       fill=c(cols.min,cols.max),border="black")
dev.off()
```

```
# combined
b.df=df.x%>%filter(Group=="Mammals")
vals1=b.df$OU_Avg

b.df=df.x%>%filter(Group=="Birds")
vals2=b.df$OU_Avg

vals=cbind(vals1,vals2)
vals=rowMeans(vals)

cols=colourvalues::colour_values(vals)

cols.max=cols[which(vals==max(vals))[1]]
cols.min=cols[which(vals==min(vals))[1]]

plot(b.df$Long,b.df$Lat,asp=1,col=cols,pch=".",
     xlab="Longitude",ylab="Latitude",main="Mammal & Bird Dispersion")
legend("bottomright",legend=c("Underdispersed","Overdispersed"),
       fill=c(cols.min,cols.max),border="black")
```

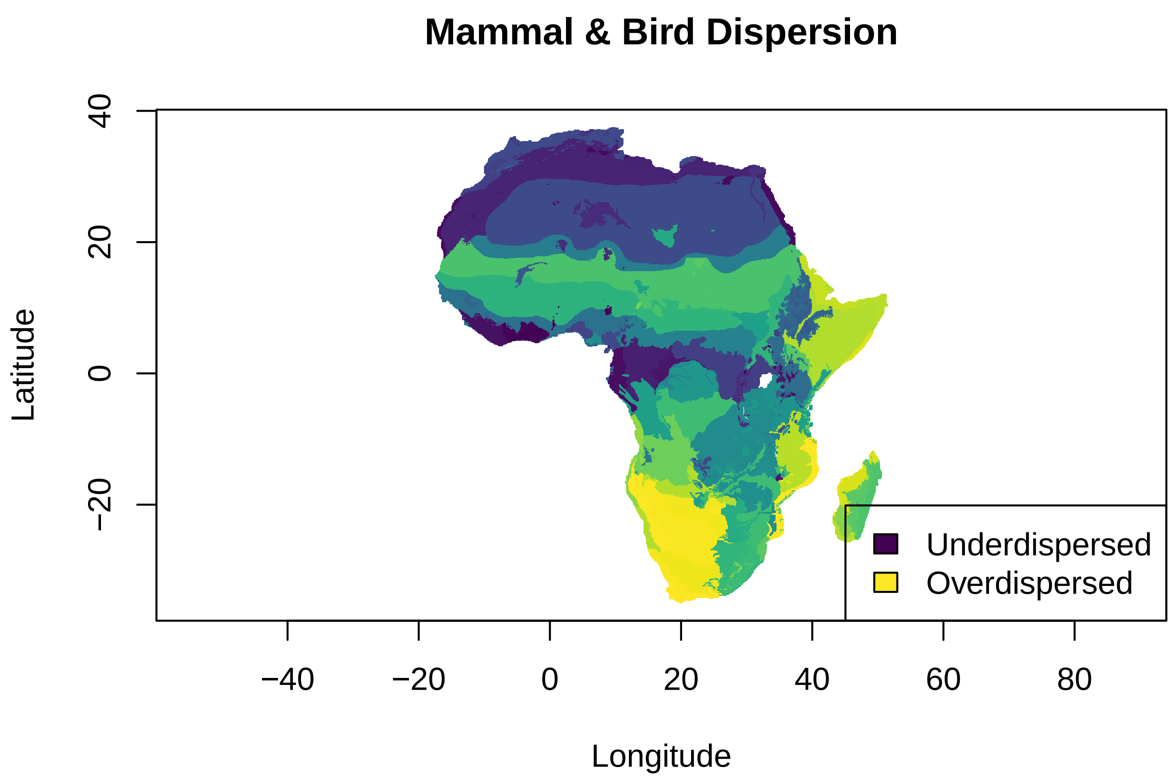


```
# plot and add legend
jpeg("mammal-n-bird_dispersion_eco.jpeg",quality=100)
plot(b.df$Long,b.df$Lat,asp=1,col=cols,pch=".",
     xlab="Longitude",ylab="Latitude",main="Mammal & Bird Dispersion")
legend("bottomright",legend=c("Underdispersed","Overdispersed"),
       fill=c(cols.min,cols.max),border="black")
dev.off()
```

5 Theoretical Analyses

Here, we perform our theoretical models of phylogenetic metrics with respect to identical communities that differ only in their extinction rates. We first define a function, `disperse.sim`, that is capable of creating random trees with a specified variable set. For all models, we hold constant low levels of speciation and extinction, and define the variable extinction via mass extinction events that are always more severe for areas that lack refugia.

5.1 Required Functions

```
# function for creating trees with specific species pools and extinction rates

disperse.sim=function(n, # number of taxa in a community
                      numbsim, # number of simulations to perform
                      lambda, # vector of speciation rates
                      mu, # vector of extinction rates
```

```
                    frac, # fraction survival (mass extinctions)
                    times){ # times at which events occur

  out.sim=sim.rateshift.taxa(n=n,
                             numbsim=numbsim,
                             lambda=lambda,
                             mu=mu,
                             frac=frac,
                             times=times,
                             complete=F) # extinct species not returned

  mntd=NULL
  mpd=NULL

  for(i in 1:numbsim){
    tips=out.sim[[i]]$tip.label

    # create matrix for performing tests
    tip.vals=rep(1,length(tips))
    tip.mat=t(as.matrix(tip.vals))
    colnames(tip.mat)=tips

    mpd[i]=mpd.query(out.sim[[i]],
                     tip.mat,
                     standardize=F) # don't standardize so they are all comparable
    mntd[i]=mntd.query(out.sim[[i]],
                       tip.mat,
                       standardize=F)
  }
  out.mat=cbind(mpd,mntd)
  return(as.data.frame(out.mat))
}

# histogram plotting function

plot.hist=function(x.stable,y.unstable,val){
  x=cbind("Stable",x.stable)
  y=cbind("Unstable",y.unstable)
  colnames(x)[1]=colnames(y)[1]="State"

  xy=rbind(x,y)
  cols=viridis(2)
  p.x=gghistogram(xy,x=val,add="mean",rug=F,
                  color="State",fill="State",
                  palette=cols,bins=30)
  print(p.x)
}
```

5.2 Tests of community makeup

5.2.1 100 species, low extinction

```r
# define parameters

numbsim=200
n.suit=100 # community size
n.unsuit=n.suit/10 # unsuitable habitat community size
frac.unstab=c(0.9, # % survival during mass extinction
              0.9,
              1)
frac.stab=1-((1-frac.unstab)/10) # mass extinction with refugia
mu=rep(0.1,3) # constant low extinction rates
lambda=rep(1.75,3) # constant speciation rates
times=c(0,0.33,0.66) # define times across simulation
```

```r
suitable.stable=disperse.sim(n = n.suit,numbsim = numbsim,
                             lambda = lambda,mu = mu,
                             frac = frac.stab,
                             times = times)

unsuitable.stable=disperse.sim(n = n.unsuit,numbsim = numbsim,
                               lambda = lambda,mu = mu,
                               frac = frac.stab,times = times)

suitable.unstable=disperse.sim(n = n.suit,numbsim = numbsim,
                               lambda = lambda,mu = mu,
                               frac = frac.unstab,times = times)

unsuitable.unstable=disperse.sim(n = n.unsuit,numbsim = numbsim,
                                 lambda = lambda,mu = mu,
                                 frac = frac.unstab,times = times)
```

```
## [1] "MPD, suitable"
```

```
## [1] "Means (stab/unstab): 3.94 / 4.08"
```

```r
t.test(suitable.stable$mpd,suitable.unstable$mpd)
```

```
##
##  Welch Two Sample t-test
##
## data:  suitable.stable$mpd and suitable.unstable$mpd
## t = -1.9616, df = 397.89, p-value = 0.0505
## alternative hypothesis: true difference in means is not equal to 0
## 95 percent confidence interval:
##  -0.2627259000  0.0002891905
## sample estimates:
## mean of x mean of y
##  3.943814  4.075032
```

```r
plot.hist(x.stable = suitable.stable,
          y.unstable = suitable.unstable,
          val = "mpd")
```

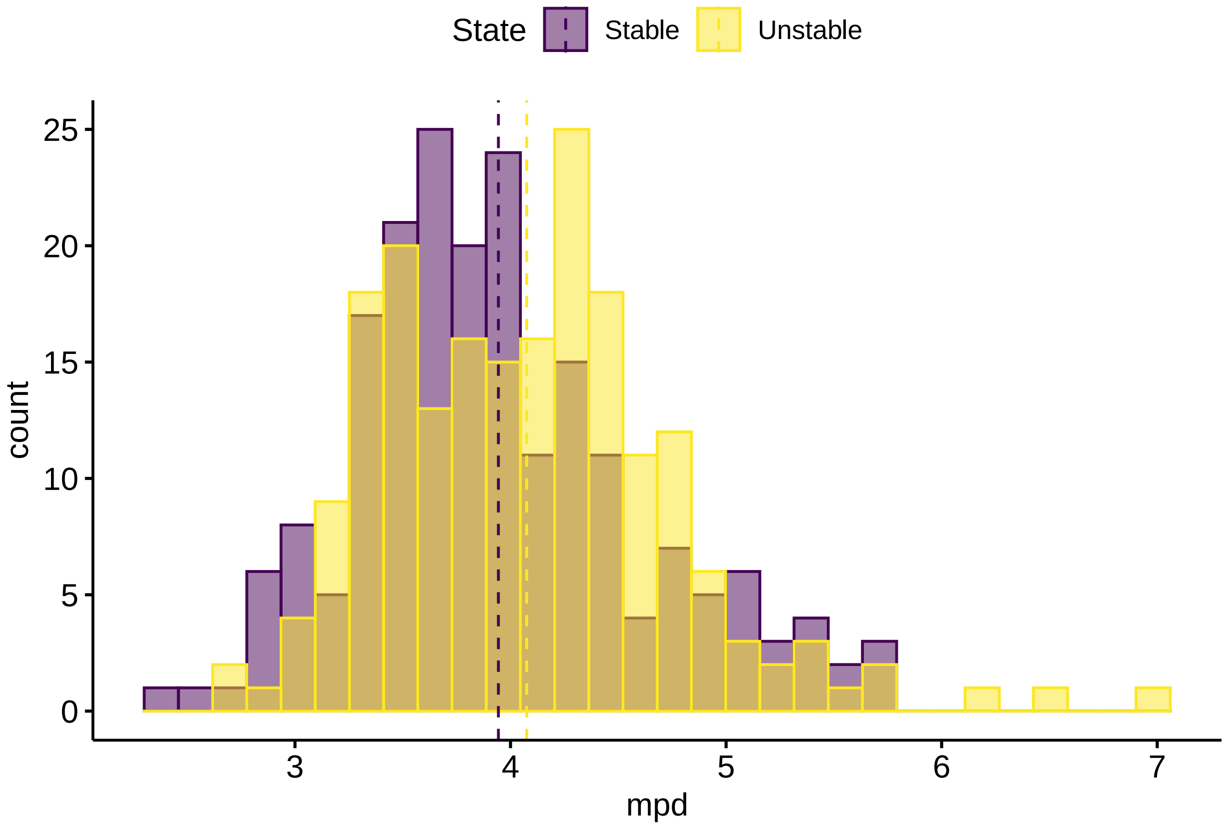


```
## [1] "MPD, unsuitable"

## [1] "Means (stab/unstab): 1.68 / 1.78"
```

```
t.test(unsuitable.stable$mpd,unsuitable.unstable$mpd)
```

```
##
##  Welch Two Sample t-test
##
## data:  unsuitable.stable$mpd and unsuitable.unstable$mpd
## t = -1.7194, df = 393.78, p-value = 0.08632
## alternative hypothesis: true difference in means is not equal to 0
## 95 percent confidence interval:
##  -0.20953485  0.01401981
## sample estimates:
## mean of x mean of y
##  1.684376  1.782134
```

```
plot.hist(x.stable = unsuitable.stable,
          y.unstable = unsuitable.unstable,
          val = "mpd")
```

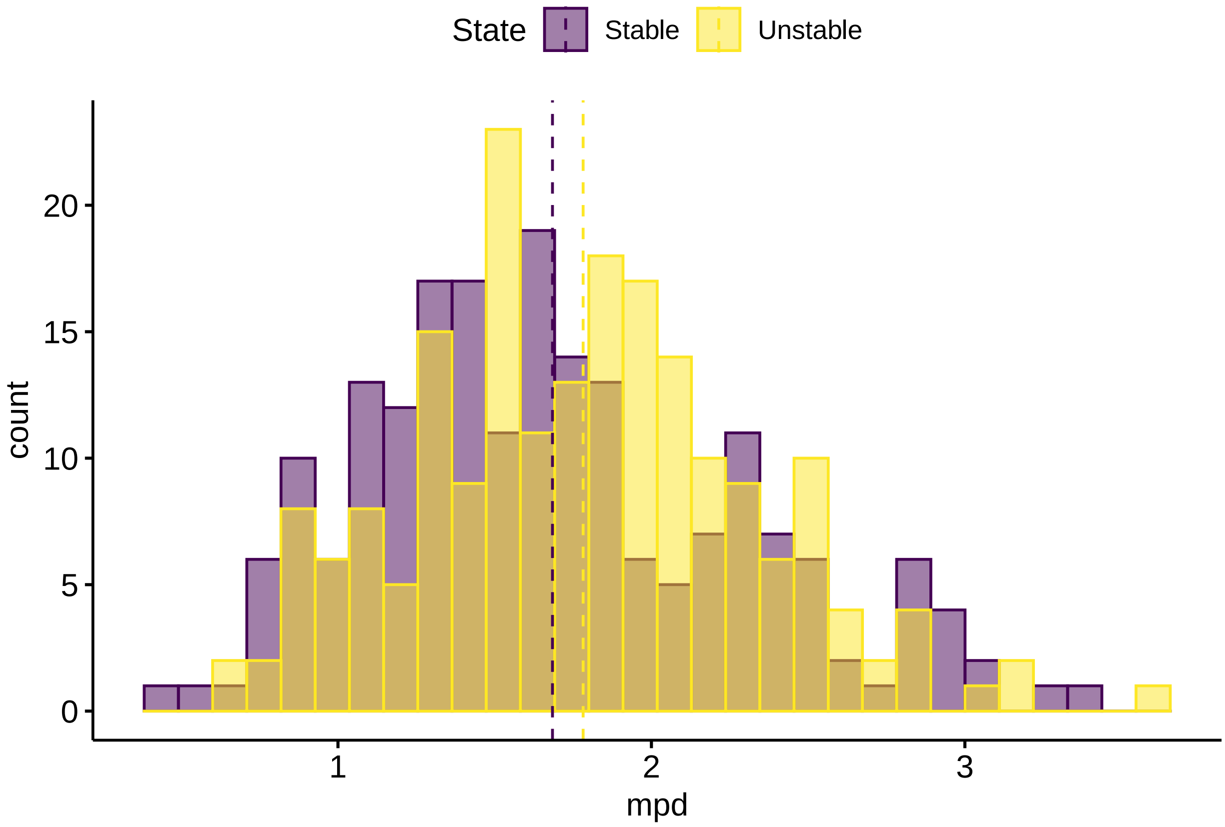


```
## [1] "MNTD, suitable"

## [1] "Means (stab/unstab): 0.59 / 0.64"
```

```
t.test(suitable.stable$mntd,suitable.unstable$mntd)
```

```
##
##  Welch Two Sample t-test
##
## data:  suitable.stable$mntd and suitable.unstable$mntd
## t = -6.4563, df = 397.8, p-value = 3.13e-10
## alternative hypothesis: true difference in means is not equal to 0
## 95 percent confidence interval:
##  -0.06564794 -0.03500037
## sample estimates:
## mean of x mean of y
## 0.5850764 0.6354006
```

```
plot.hist(x.stable = suitable.stable,
          y.unstable = suitable.unstable,
          val = "mntd")
```

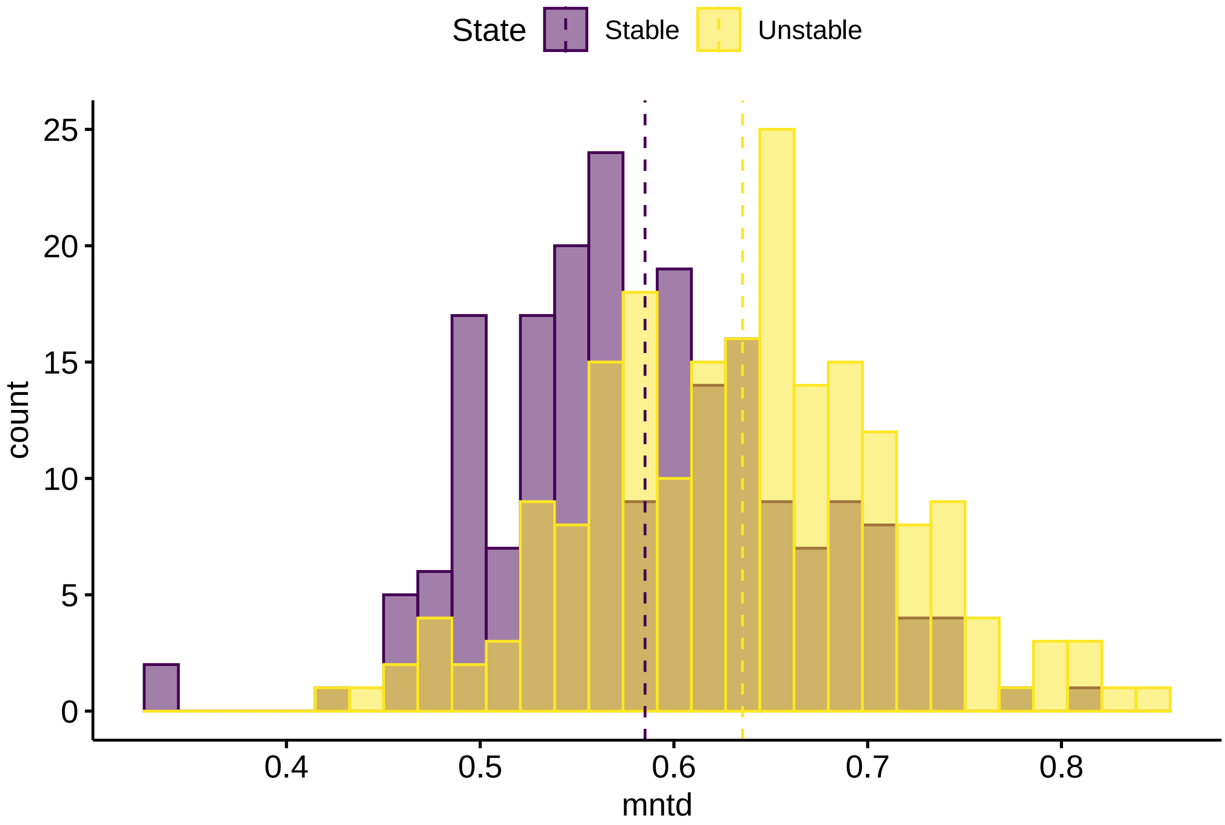


```
## [1] "MNTD, unsuitable"

## [1] "Means (stab/unstab): 0.61 / 0.66"
```

```
t.test(unsuitable.stable$mntd,unsuitable.unstable$mntd)
```

```
##
##  Welch Two Sample t-test
##
## data:  unsuitable.stable$mntd and unsuitable.unstable$mntd
## t = -1.9217, df = 397.63, p-value = 0.05536
## alternative hypothesis: true difference in means is not equal to 0
## 95 percent confidence interval:
##  -0.100825812  0.001148316
## sample estimates:
## mean of x mean of y
## 0.6082904 0.6581291
```

```
plot.hist(x.stable = unsuitable.stable,
          y.unstable = unsuitable.unstable,
          val = "mntd")
```

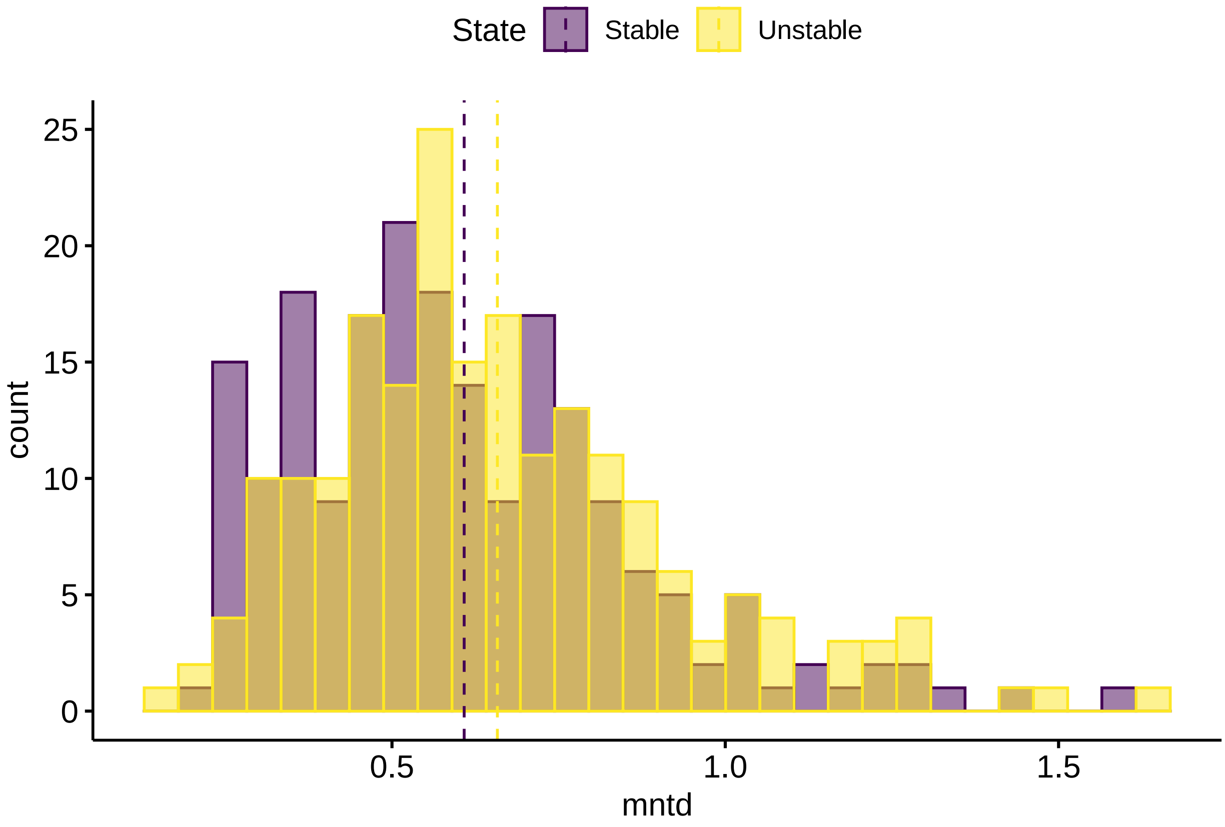

5.2.2 100 species, high extinction

```
# define parameters

numbsim=200
n.suit=100 # community size
n.unsuit=n.suit/10 # unsuitable habitat community size
frac.unstab=c(0.3, # % survival during mass extinction
              0.3,
              1)
frac.stab=1-((1-frac.unstab)/10) # mass extinction with refugia
mu=rep(0.1,3) # constant low extinction rates
lambda=rep(1.75,3) # constant speciation rates
times=c(0,0.33,0.66) # define times across simulation
```

```
suitable.stable=disperse.sim(n = n.suit,numbsim = numbsim,
                             lambda = lambda,mu = mu,
                             frac = frac.stab,
                             times = times)

unsuitable.stable=disperse.sim(n = n.unsuit,numbsim = numbsim,
                               lambda = lambda,mu = mu,
                               frac = frac.stab,times = times)

suitable.unstable=disperse.sim(n = n.suit,numbsim = numbsim,
                               lambda = lambda,mu = mu,
```

```
                                frac = frac.unstab,times = times)

unsuitable.unstable=disperse.sim(n = n.unsuit,numbsim = numbsim,
                                 lambda = lambda,mu = mu,
                                 frac = frac.unstab,times = times)
```

```
## [1] "MPD, suitable"

## [1] "Means (stab/unstab): 4.06 / 6.71"
```

```
t.test(suitable.stable$mpd,suitable.unstable$mpd)
```

```
##
##  Welch Two Sample t-test
##
## data:  suitable.stable$mpd and suitable.unstable$mpd
## t = -41.517, df = 397.99, p-value < 2.2e-16
## alternative hypothesis: true difference in means is not equal to 0
## 95 percent confidence interval:
##  -2.774845 -2.523933
## sample estimates:
## mean of x mean of y
##  4.062587  6.711976
```

```
plot.hist(x.stable = suitable.stable,
          y.unstable = suitable.unstable,
          val = "mpd")
```

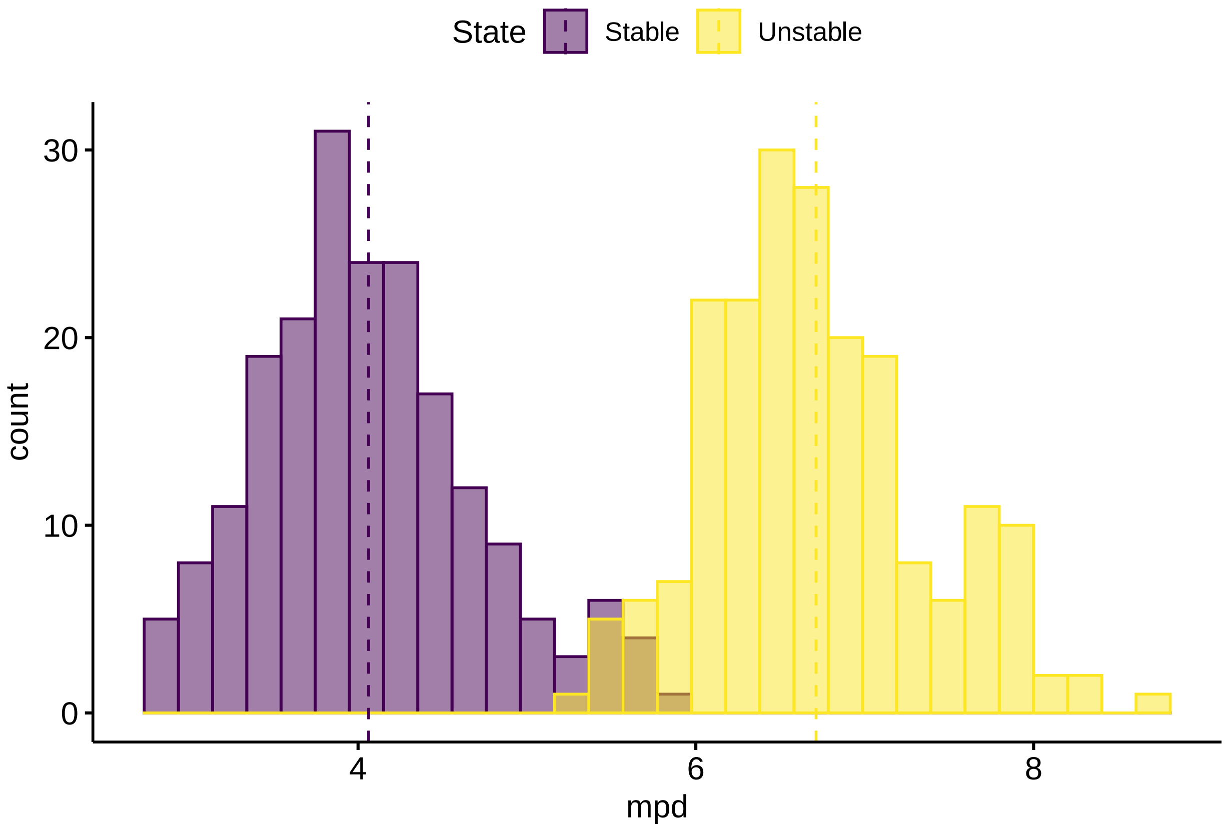

```
## [1] "MPD, unsuitable"
## [1] "Means (stab/unstab): 1.73 / 3.89"
```

```
t.test(unsuitable.stable$mpd,unsuitable.unstable$mpd)
```

```
##
##  Welch Two Sample t-test
##
## data:  unsuitable.stable$mpd and unsuitable.unstable$mpd
## t = -32.999, df = 368.81, p-value < 2.2e-16
## alternative hypothesis: true difference in means is not equal to 0
## 95 percent confidence interval:
##  -2.289039 -2.031577
## sample estimates:
## mean of x mean of y
##  1.733259  3.893567
```

```
plot.hist(x.stable = unsuitable.stable,
          y.unstable = unsuitable.unstable,
          val = "mpd")
```

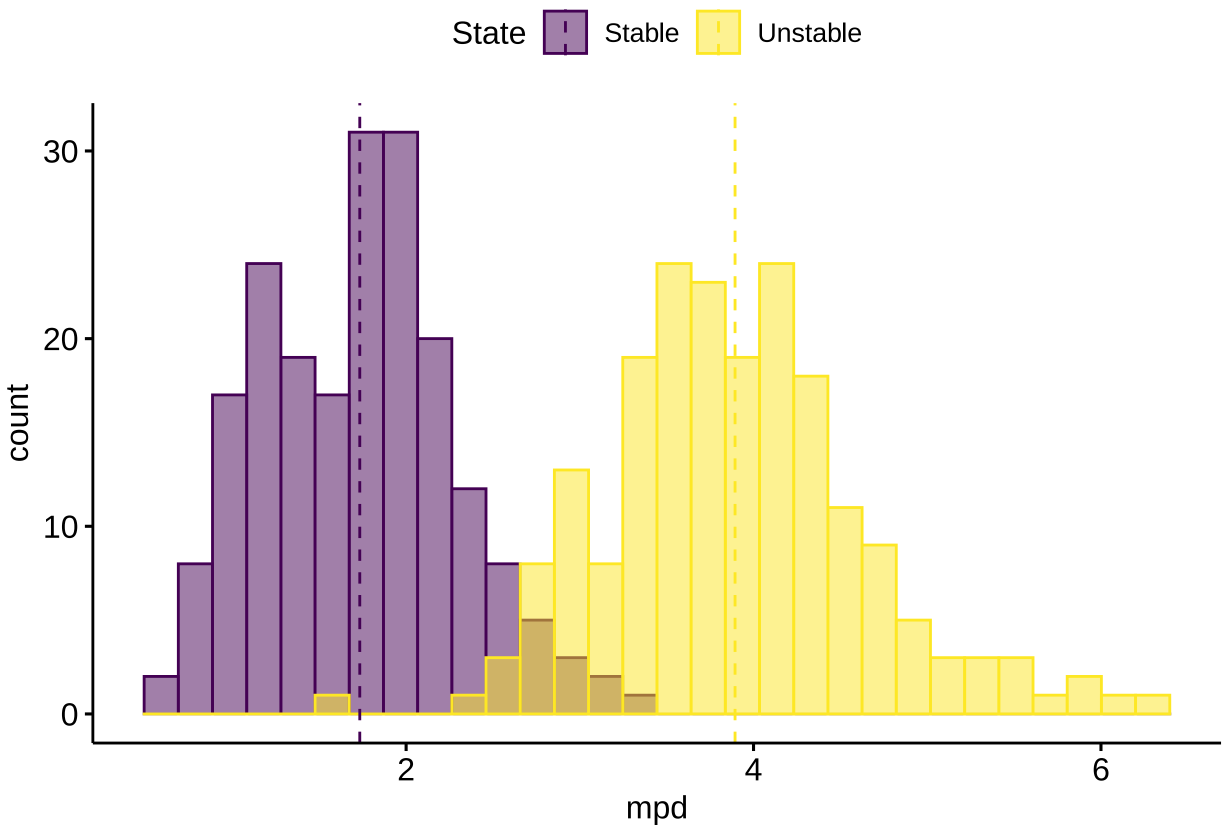


```
## [1] "MNTD, suitable"
## [1] "Means (stab/unstab): 0.63 / 1.77"
```

```
t.test(suitable.stable$mntd,suitable.unstable$mntd)
```

```
##
```

```
##  Welch Two Sample t-test
##
## data:  suitable.stable$mntd and suitable.unstable$mntd
## t = -89.703, df = 298.52, p-value < 2.2e-16
## alternative hypothesis: true difference in means is not equal to 0
## 95 percent confidence interval:
##  -1.159304 -1.109530
## sample estimates:
## mean of x mean of y
## 0.6311898 1.7656067
```

```
plot.hist(x.stable = suitable.stable,
          y.unstable = suitable.unstable,
          val = "mntd")
```

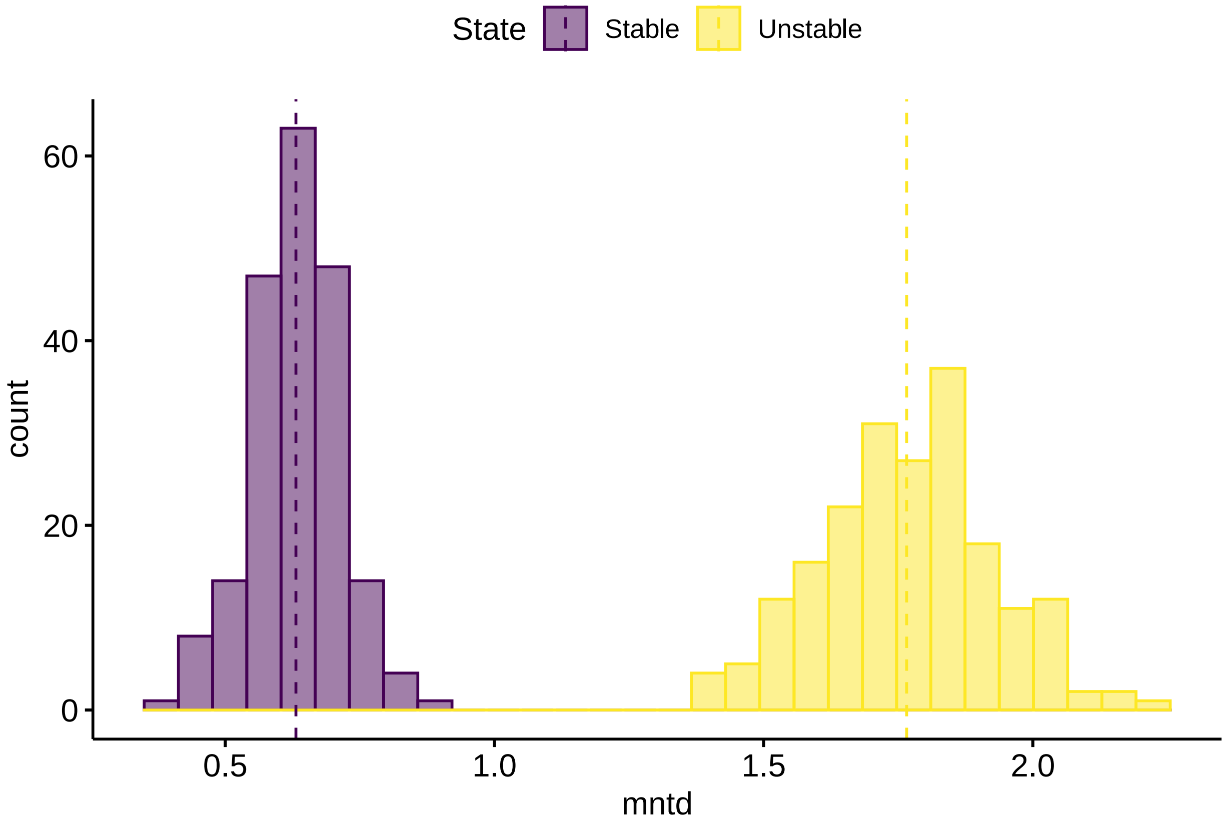


```
## [1] "MNTD, unsuitable"

## [1] "Means (stab/unstab): 0.65 / 1.81"
```

```
t.test(unsuitable.stable$mntd,unsuitable.unstable$mntd)
```

```
##
##  Welch Two Sample t-test
##
## data:  unsuitable.stable$mntd and unsuitable.unstable$mntd
## t = -29.894, df = 303.39, p-value < 2.2e-16
## alternative hypothesis: true difference in means is not equal to 0
## 95 percent confidence interval:
```

```
##  -1.239328 -1.086242
## sample estimates:
## mean of x mean of y
## 0.6496413 1.8124260
```

```
plot.hist(x.stable = unsuitable.stable,
          y.unstable = unsuitable.unstable,
          val = "mntd")
```

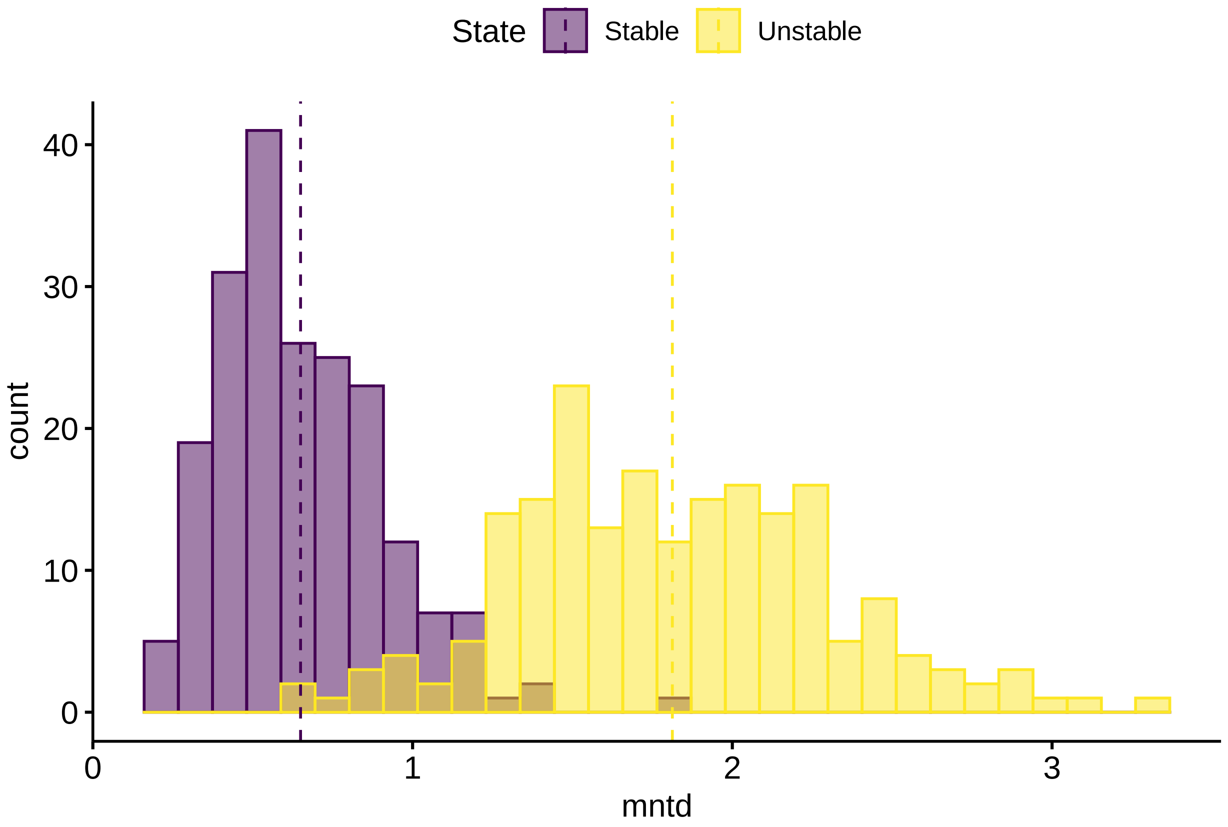

5.2.3 100 species, high extinction in past

```
# define parameters

numbsim=200
n.suit=100 # community size
n.unsuit=n.suit/10 # unsuitable habitat community size
frac.unstab=c(0.2, # % survival during mass extinction
              0.9,
              1)
frac.stab=1-((1-frac.unstab)/10) # mass extinction with refugia
mu=rep(0.1,3) # constant low extinction rates
lambda=rep(1.75,3) # constant speciation rates
times=c(0,0.33,0.66) # define times across simulation
```

```
suitable.stable=disperse.sim(n = n.suit,numbsim = numbsim,
                             lambda = lambda,mu = mu,
                             frac = frac.stab,
```

```
                              times = times)

unsuitable.stable=disperse.sim(n = n.unsuit,numbsim = numbsim,
                               lambda = lambda,mu = mu,
                               frac = frac.stab,times = times)

suitable.unstable=disperse.sim(n = n.suit,numbsim = numbsim,
                               lambda = lambda,mu = mu,
                               frac = frac.unstab,times = times)

unsuitable.unstable=disperse.sim(n = n.unsuit,numbsim = numbsim,
                                 lambda = lambda,mu = mu,
                                 frac = frac.unstab,times = times)
```

```
## [1] "MPD, suitable"
```

```
## [1] "Means (stab/unstab): 3.99 / 5.88"
```

```
t.test(suitable.stable$mpd,suitable.unstable$mpd)
```

```
##
##  Welch Two Sample t-test
##
## data:  suitable.stable$mpd and suitable.unstable$mpd
## t = -30.95, df = 396.52, p-value < 2.2e-16
## alternative hypothesis: true difference in means is not equal to 0
## 95 percent confidence interval:
##  -2.013782 -1.773231
## sample estimates:
## mean of x mean of y
##  3.986418  5.879925
```

```
plot.hist(x.stable = suitable.stable,
          y.unstable = suitable.unstable,
          val = "mpd")
```

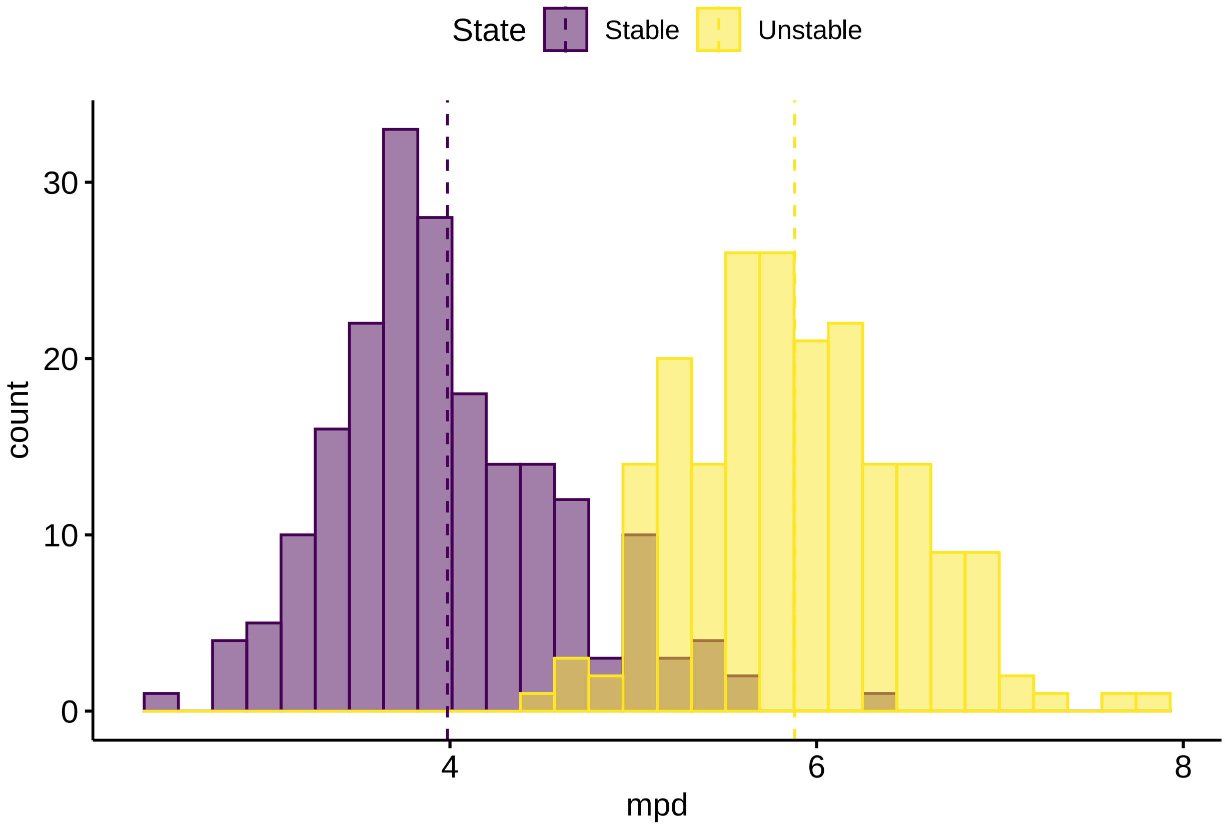


```
## [1] "MPD, unsuitable"
```

```
## [1] "Means (stab/unstab): 1.7 / 3.27"
```

```
t.test(unsuitable.stable$mpd,unsuitable.unstable$mpd)
```

```
##
##  Welch Two Sample t-test
##
## data:  unsuitable.stable$mpd and unsuitable.unstable$mpd
## t = -24.42, df = 391.67, p-value < 2.2e-16
## alternative hypothesis: true difference in means is not equal to 0
## 95 percent confidence interval:
##  -1.698317 -1.445234
## sample estimates:
## mean of x mean of y
##  1.695085  3.266861
```

```
plot.hist(x.stable = unsuitable.stable,
          y.unstable = unsuitable.unstable,
          val = "mpd")
```

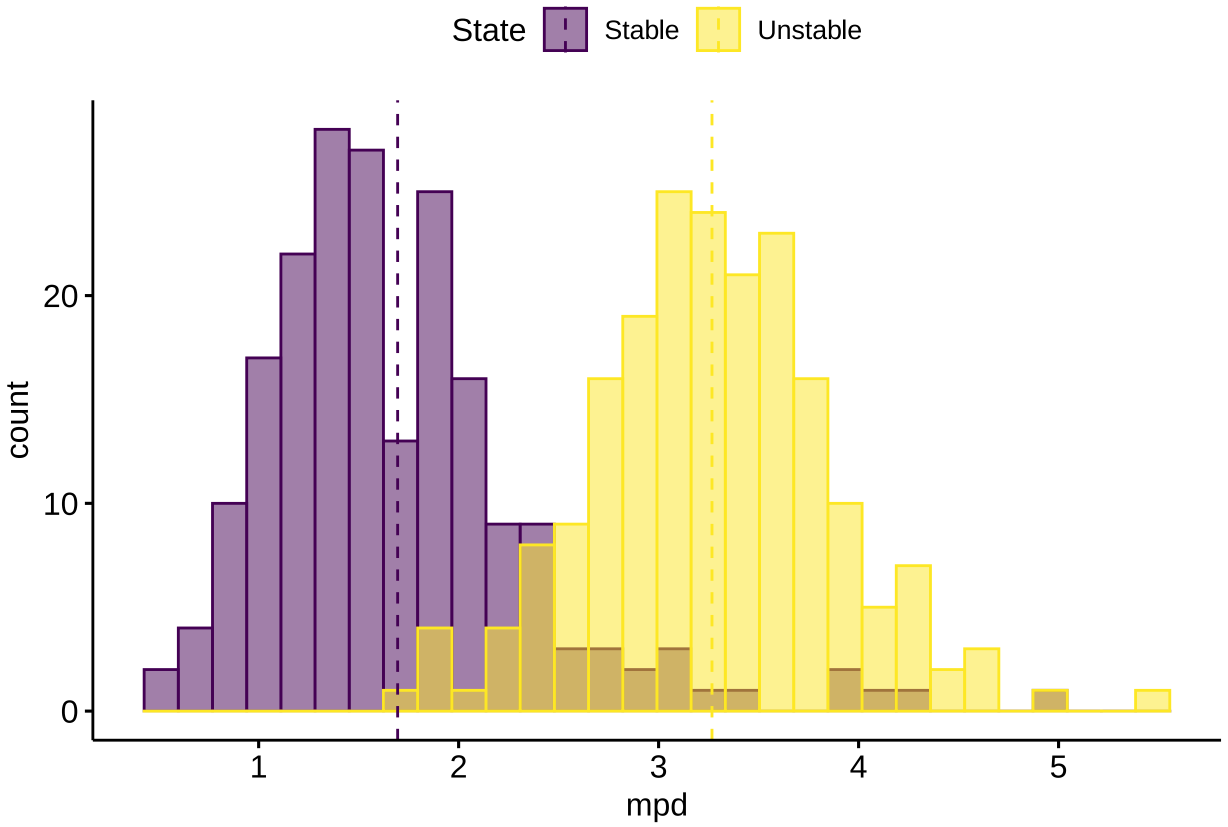


```
## [1] "MNTD, suitable"

## [1] "Means (stab/unstab): 0.62 / 1.55"
```

```
t.test(suitable.stable$mntd,suitable.unstable$mntd)
```

```
##
##  Welch Two Sample t-test
##
## data:  suitable.stable$mntd and suitable.unstable$mntd
## t = -83.027, df = 322.28, p-value < 2.2e-16
## alternative hypothesis: true difference in means is not equal to 0
## 95 percent confidence interval:
##  -0.9572087 -0.9128962
## sample estimates:
## mean of x mean of y
## 0.6199362 1.5549887
```

```
plot.hist(x.stable = suitable.stable,
          y.unstable = suitable.unstable,
          val = "mntd")
```

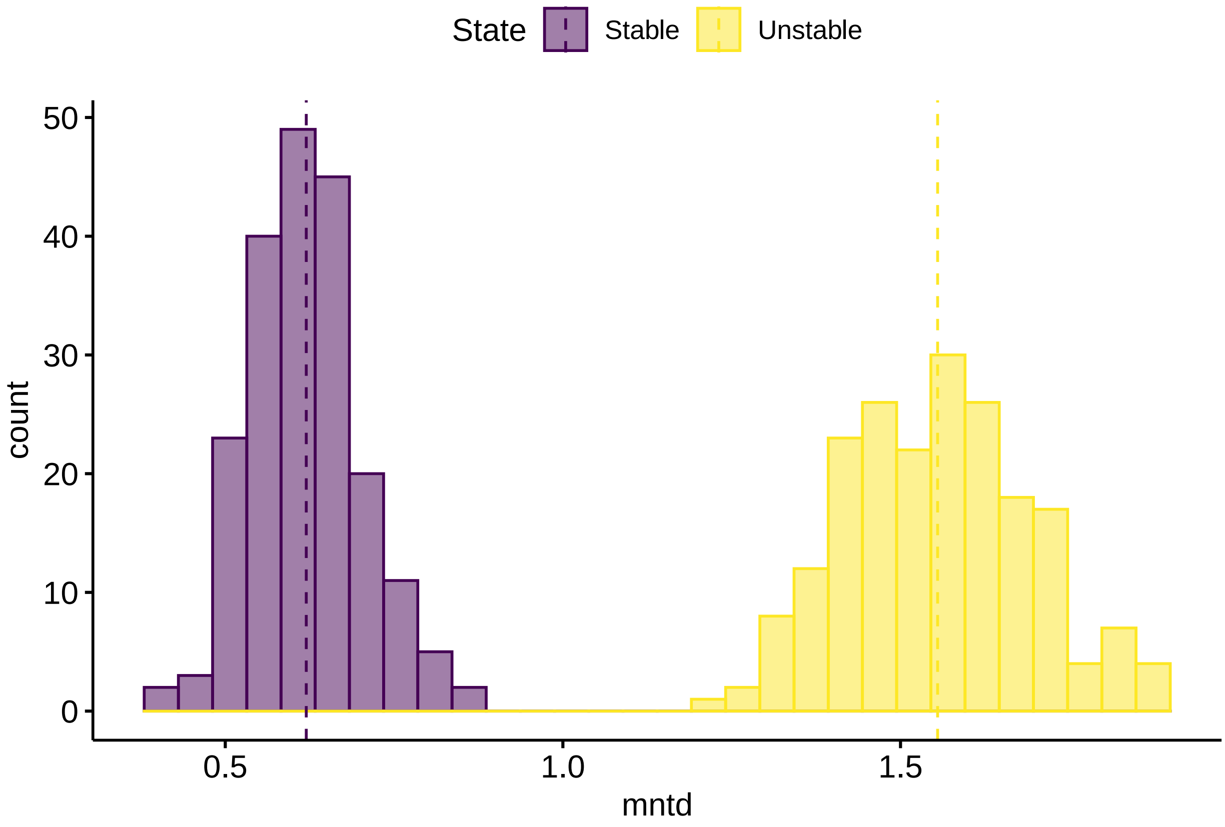


```
## [1] "MNTD, unsuitable"
## [1] "Means (stab/unstab): 0.63 / 1.6"
```

```
t.test(unsuitable.stable$mntd,unsuitable.unstable$mntd)
```

```
##
##  Welch Two Sample t-test
##
## data:  unsuitable.stable$mntd and unsuitable.unstable$mntd
## t = -27.935, df = 329.75, p-value < 2.2e-16
## alternative hypothesis: true difference in means is not equal to 0
## 95 percent confidence interval:
##  -1.0402470 -0.9033761
## sample estimates:
## mean of x mean of y
##  0.625558  1.597370
```

```
plot.hist(x.stable = unsuitable.stable,
          y.unstable = unsuitable.unstable,
          val = "mntd")
```

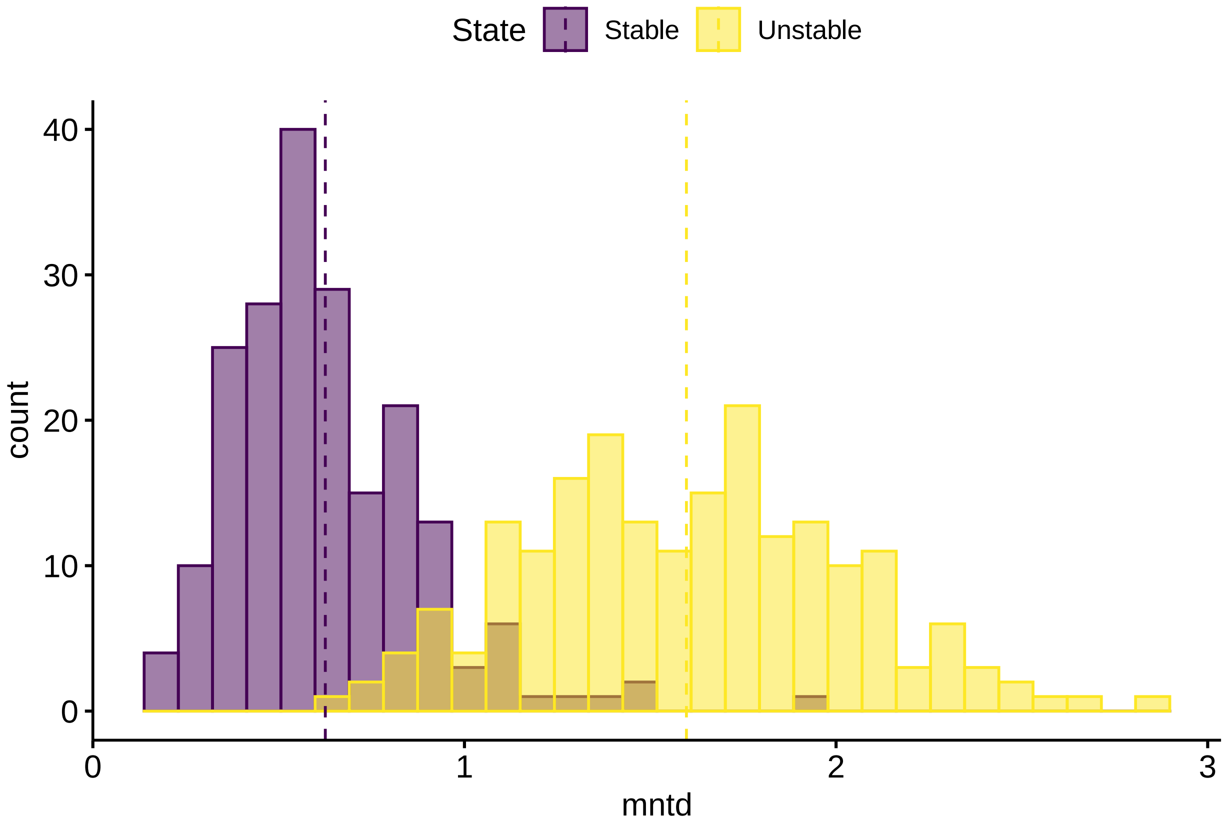

5.2.4 100 species, high recent extinction

```
# define parameters

numbsim=200
n.suit=100 # community size
n.unsuit=n.suit/10 # unsuitable habitat community size
frac.unstab=c(1, # % survival during mass extinction
              0.9,
              0.3)
frac.stab=1-((1-frac.unstab)/10) # mass extinction with refugia
mu=rep(0.1,3) # constant low extinction rates
lambda=rep(1.75,3) # constant speciation rates
times=c(0,0.33,0.66) # define times across simulation
```

```
suitable.stable=disperse.sim(n = n.suit,numbsim = numbsim,
                             lambda = lambda,mu = mu,
                             frac = frac.stab,
                             times = times)

unsuitable.stable=disperse.sim(n = n.unsuit,numbsim = numbsim,
                               lambda = lambda,mu = mu,
                               frac = frac.stab,times = times)

suitable.unstable=disperse.sim(n = n.suit,numbsim = numbsim,
                               lambda = lambda,mu = mu,
```

```
                                frac = frac.unstab,times = times)

unsuitable.unstable=disperse.sim(n = n.unsuit,numbsim = numbsim,
                                 lambda = lambda,mu = mu,
                                 frac = frac.unstab,times = times)
```

```
## [1] "MPD, suitable"

## [1] "Means (stab/unstab): 4 / 5.27"
```

```
t.test(suitable.stable$mpd,suitable.unstable$mpd)
```

```
##
##  Welch Two Sample t-test
##
## data:  suitable.stable$mpd and suitable.unstable$mpd
## t = -20.119, df = 397.94, p-value < 2.2e-16
## alternative hypothesis: true difference in means is not equal to 0
## 95 percent confidence interval:
##  -1.388244 -1.141088
## sample estimates:
## mean of x mean of y
##  4.002896  5.267562
```

```
plot.hist(x.stable = suitable.stable,
          y.unstable = suitable.unstable,
          val = "mpd")
```

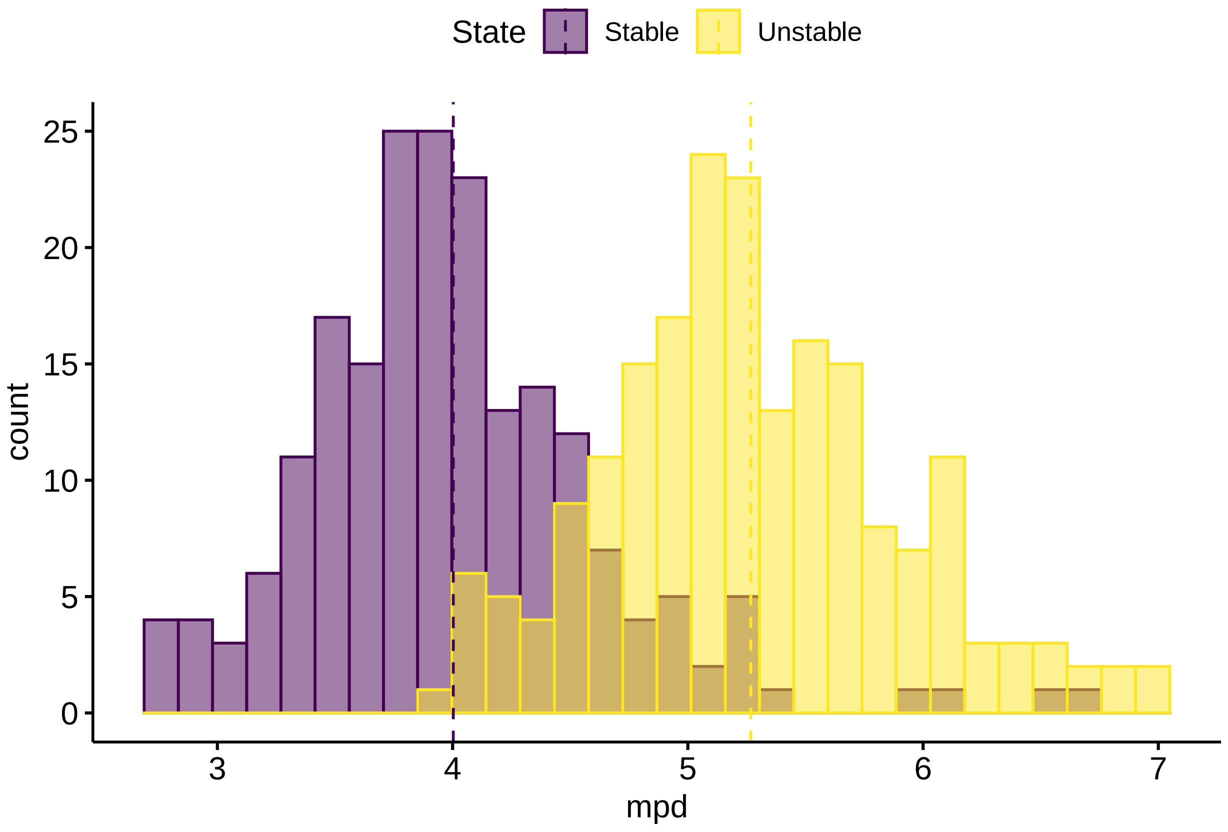

```
## [1] "MPD, unsuitable"

## [1] "Means (stab/unstab): 1.57 / 2.31"
```

```
t.test(unsuitable.stable$mpd,unsuitable.unstable$mpd)
```

```
##
##  Welch Two Sample t-test
##
## data:  unsuitable.stable$mpd and unsuitable.unstable$mpd
## t = -9.9688, df = 332.96, p-value < 2.2e-16
## alternative hypothesis: true difference in means is not equal to 0
## 95 percent confidence interval:
##  -0.8823061 -0.5914875
## sample estimates:
## mean of x mean of y
##  1.569785  2.306682
```

```
plot.hist(x.stable = unsuitable.stable,
          y.unstable = unsuitable.unstable,
          val = "mpd")
```

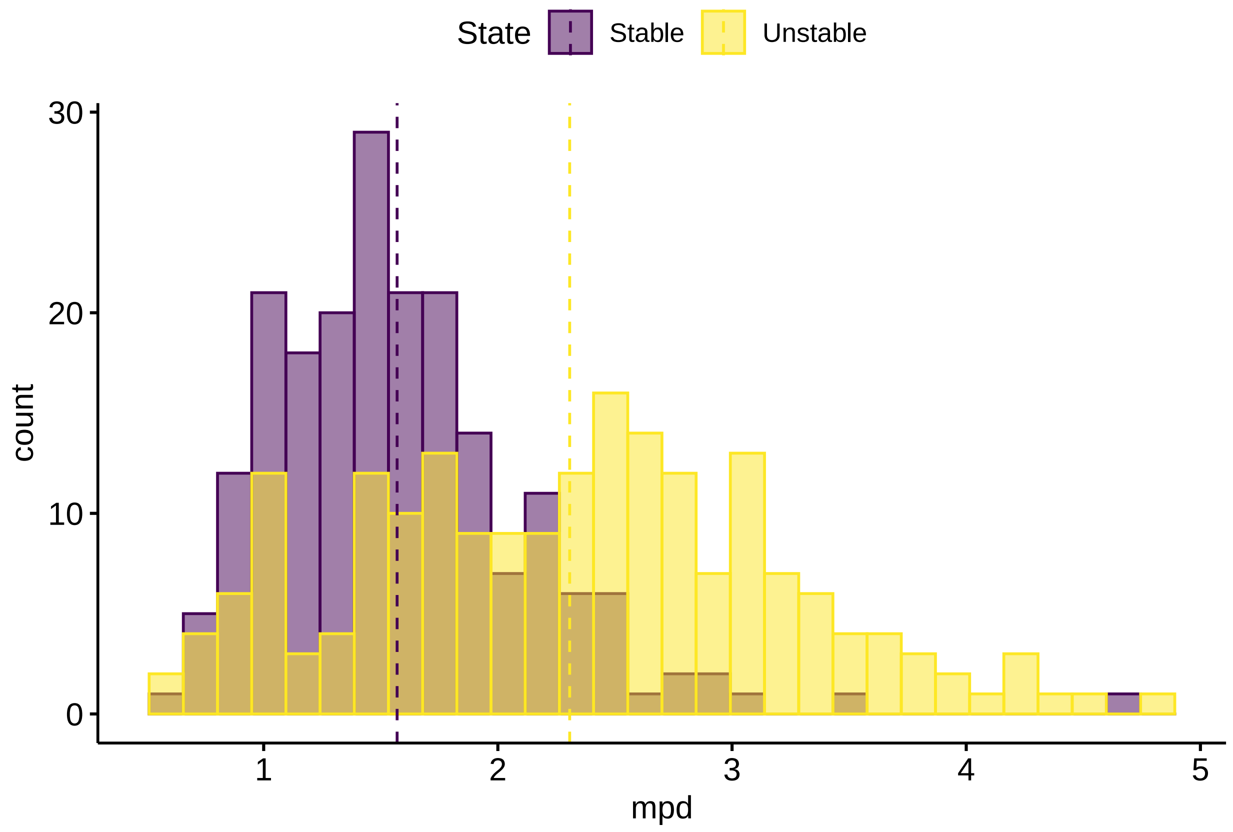


```
## [1] "MNTD, suitable"

## [1] "Means (stab/unstab): 0.59 / 0.68"
```

```
t.test(suitable.stable$mntd,suitable.unstable$mntd)
```

```
##
```

```
##  Welch Two Sample t-test
##
## data:  suitable.stable$mntd and suitable.unstable$mntd
## t = -10.838, df = 378.7, p-value < 2.2e-16
## alternative hypothesis: true difference in means is not equal to 0
## 95 percent confidence interval:
##  -0.11138553 -0.07717744
## sample estimates:
## mean of x mean of y
## 0.5857064 0.6799879
```

```
plot.hist(x.stable = suitable.stable,
          y.unstable = suitable.unstable,
          val = "mntd")
```

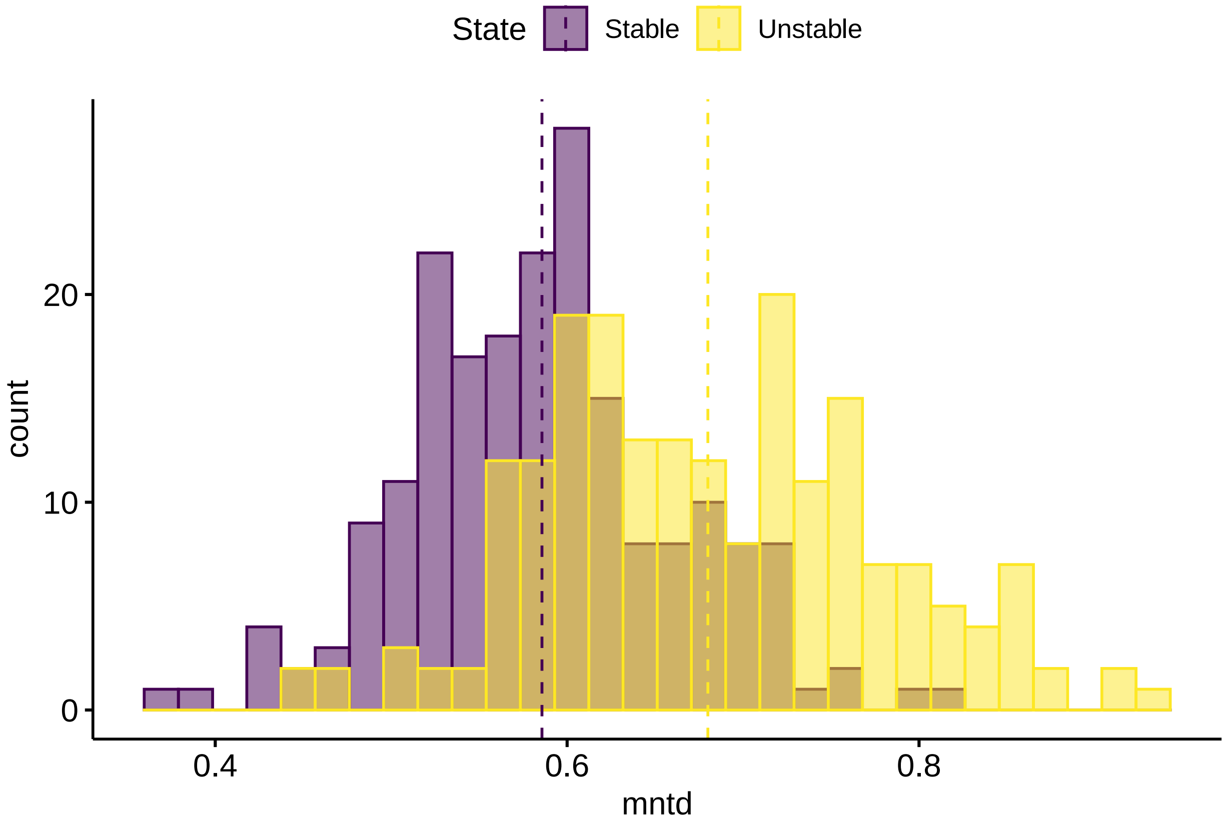


```
## [1] "MNTD, unsuitable"

## [1] "Means (stab/unstab): 0.57 / 0.7"
```

```
t.test(unsuitable.stable$mntd,unsuitable.unstable$mntd)
```

```
##
##  Welch Two Sample t-test
##
## data:  unsuitable.stable$mntd and unsuitable.unstable$mntd
## t = -4.6328, df = 346.75, p-value = 5.112e-06
## alternative hypothesis: true difference in means is not equal to 0
## 95 percent confidence interval:
```

```
##  -0.18598780 -0.07513086
## sample estimates:
## mean of x mean of y
## 0.5693162 0.6998756
```

```
plot.hist(x.stable = unsuitable.stable,
          y.unstable = unsuitable.unstable,
          val = "mntd")
```

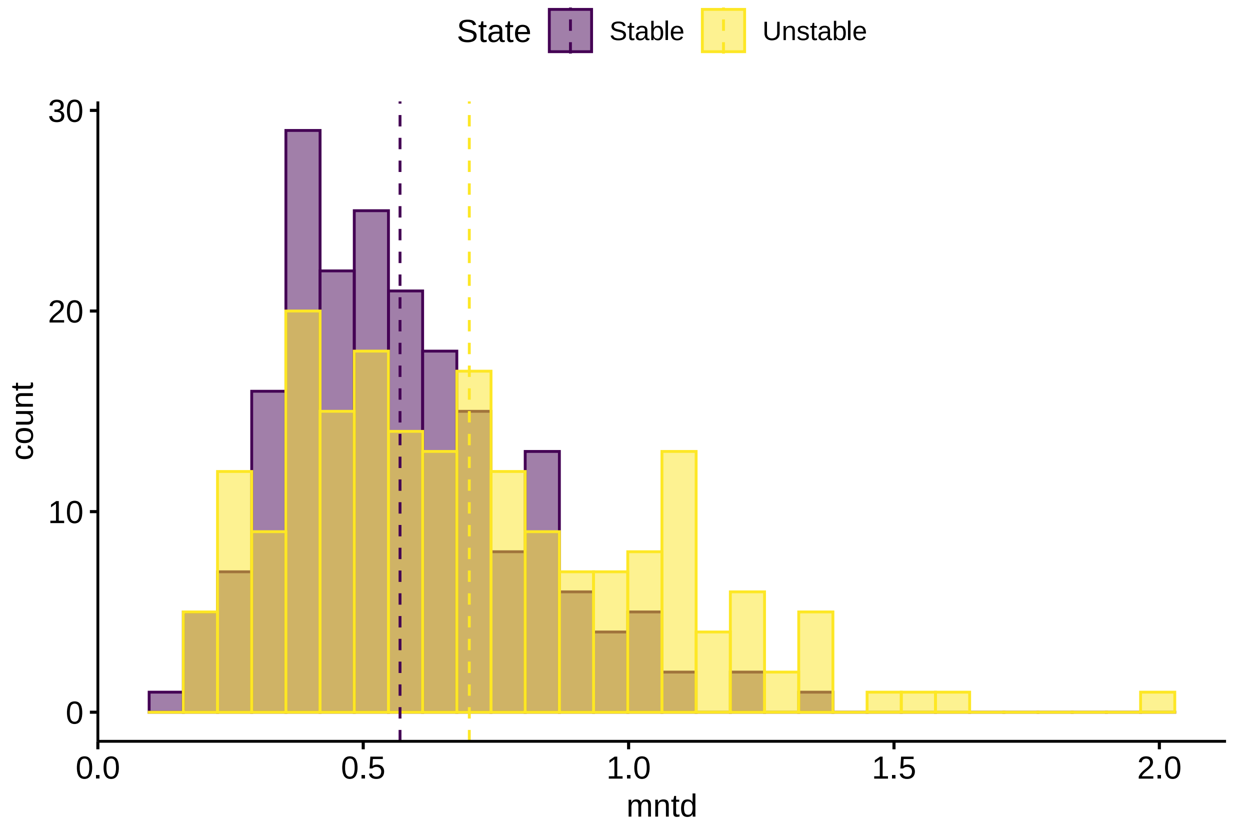

5.2.5 100 species, high extinction for both

```
# define parameters

numbsim=200
n.suit=100 # community size
n.unsuit=n.suit/10 # unsuitable habitat community size
frac.unstab=c(0.45, # % survival during mass extinction
              0.45,
              1)
frac.stab=c(0.5,0.5,1)
mu=rep(0.1,3) # constant low extinction rates
lambda=rep(1.75,3) # constant speciation rates
times=c(0,0.33,0.66) # define times across simulation
```

```
suitable.stable=disperse.sim(n = n.suit,numbsim = numbsim,
                             lambda = lambda,mu = mu,
                             frac = frac.stab,
```

```
                             times = times)

unsuitable.stable=disperse.sim(n = n.unsuit,numbsim = numbsim,
                               lambda = lambda,mu = mu,
                               frac = frac.stab,times = times)

suitable.unstable=disperse.sim(n = n.suit,numbsim = numbsim,
                               lambda = lambda,mu = mu,
                               frac = frac.unstab,times = times)

unsuitable.unstable=disperse.sim(n = n.unsuit,numbsim = numbsim,
                                 lambda = lambda,mu = mu,
                                 frac = frac.unstab,times = times)
```

```
## [1] "MPD, suitable"
```

```
## [1] "Means (stab/unstab): 5.5 / 5.71"
```

```
t.test(suitable.stable$mpd,suitable.unstable$mpd)
```

```
##
##  Welch Two Sample t-test
##
## data:  suitable.stable$mpd and suitable.unstable$mpd
## t = -3.5653, df = 397.17, p-value = 0.0004078
## alternative hypothesis: true difference in means is not equal to 0
## 95 percent confidence interval:
##  -0.33619824 -0.09720964
## sample estimates:
## mean of x mean of y
##  5.496357  5.713061
```

```
plot.hist(x.stable = suitable.stable,
          y.unstable = suitable.unstable,
          val = "mpd")
```

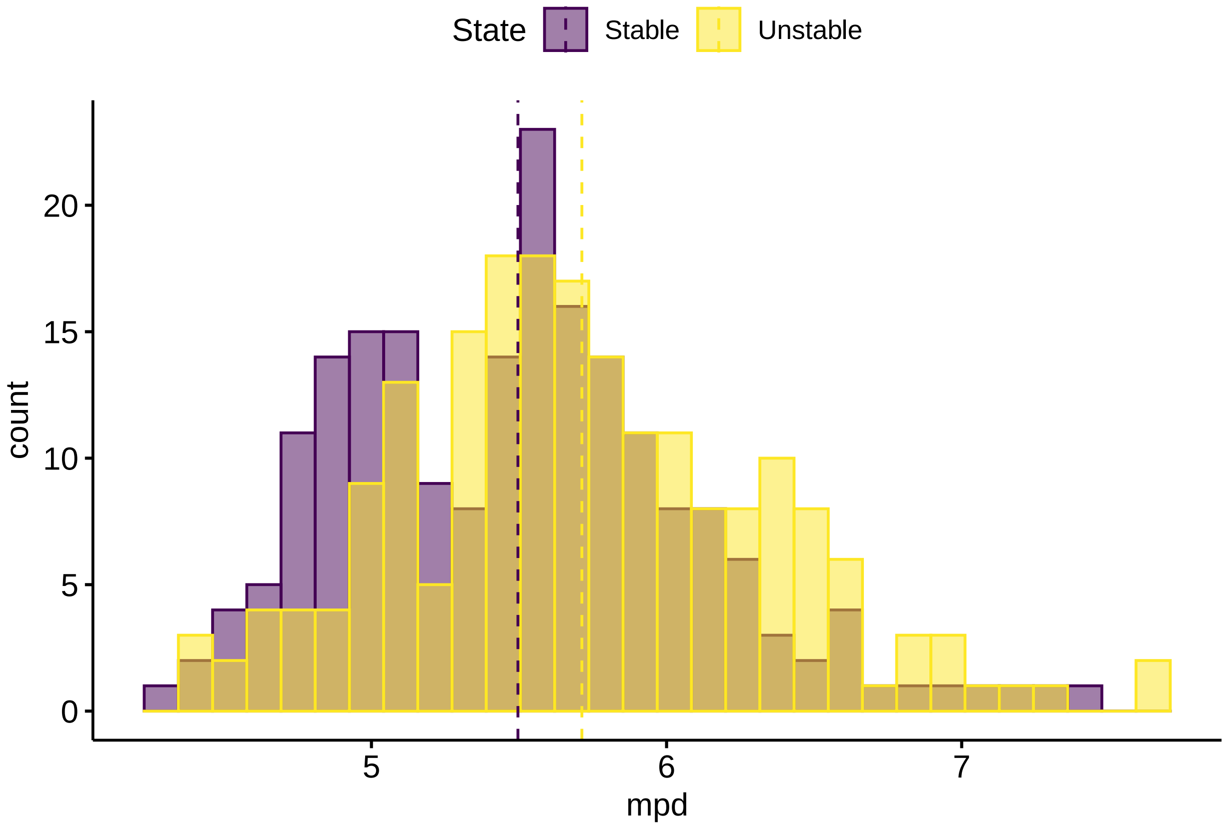


```
## [1] "MPD, unsuitable"

## [1] "Means (stab/unstab): 2.8 / 3"
```

```
t.test(unsuitable.stable$mpd,unsuitable.unstable$mpd)
```

```
##
##  Welch Two Sample t-test
##
## data:  unsuitable.stable$mpd and unsuitable.unstable$mpd
## t = -2.8447, df = 397.01, p-value = 0.004676
## alternative hypothesis: true difference in means is not equal to 0
## 95 percent confidence interval:
##  -0.32951230 -0.06018805
## sample estimates:
## mean of x mean of y
##   2.80203   2.99688
```

```
plot.hist(x.stable = unsuitable.stable,
          y.unstable = unsuitable.unstable,
          val = "mpd")
```

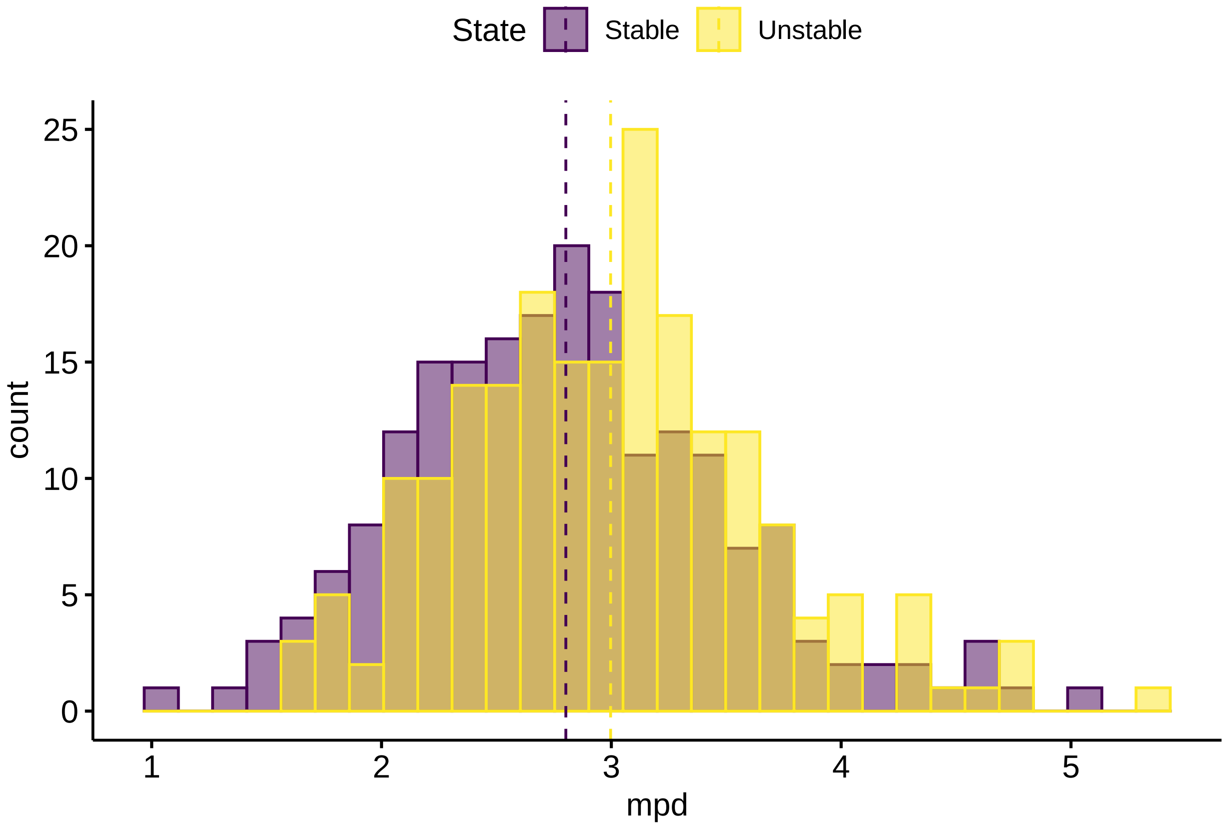


```
## [1] "MNTD, suitable"
```

```
## [1] "Means (stab/unstab): 1.12 / 1.24"
```

```
t.test(suitable.stable$mntd,suitable.unstable$mntd)
```

```
##
##  Welch Two Sample t-test
##
## data:  suitable.stable$mntd and suitable.unstable$mntd
## t = -9.4939, df = 394.79, p-value < 2.2e-16
## alternative hypothesis: true difference in means is not equal to 0
## 95 percent confidence interval:
##  -0.1439925 -0.0945875
## sample estimates:
## mean of x mean of y
##  1.116599  1.235889
```

```
plot.hist(x.stable = suitable.stable,
          y.unstable = suitable.unstable,
          val = "mntd")
```

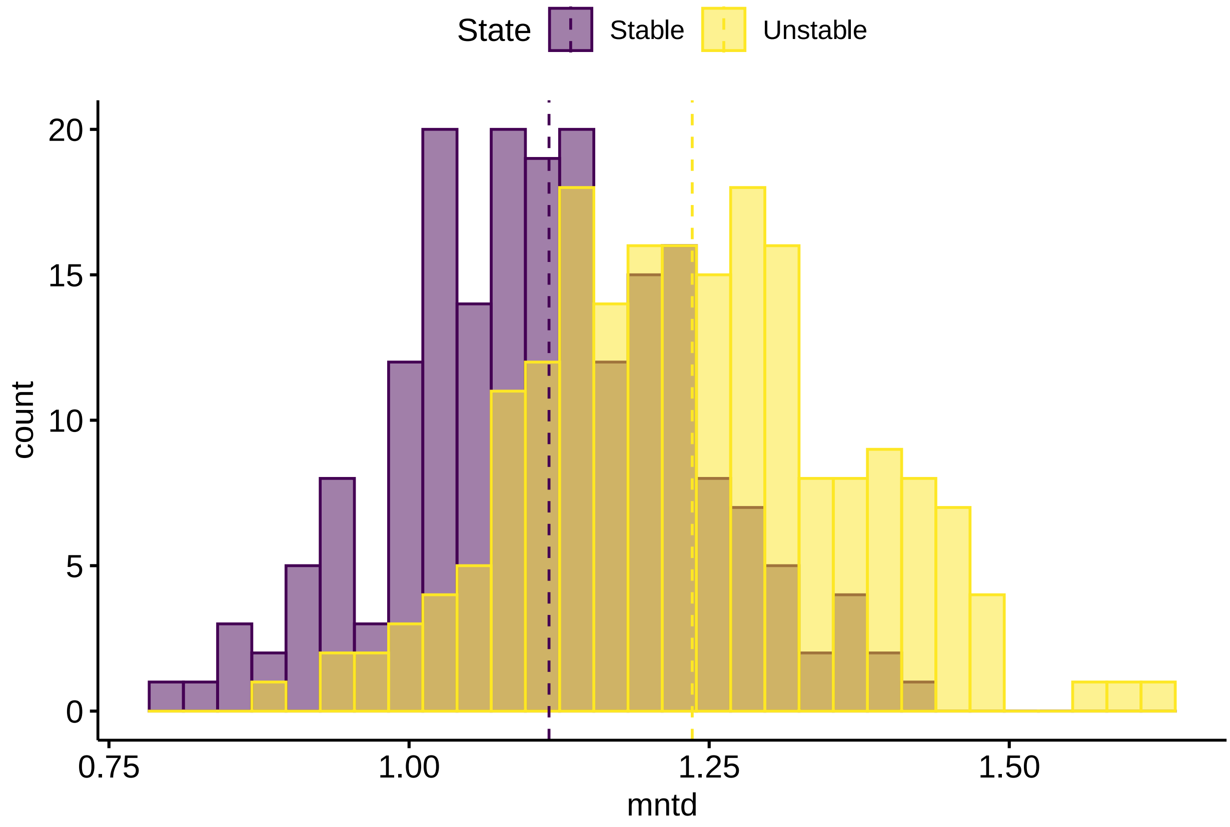


```
## [1] "MNTD, unsuitable"

## [1] "Means (stab/unstab): 1.12 / 1.25"
```

```
t.test(unsuitable.stable$mntd,unsuitable.unstable$mntd)
```

```
##
##  Welch Two Sample t-test
##
## data:  unsuitable.stable$mntd and unsuitable.unstable$mntd
## t = -3.1927, df = 397.32, p-value = 0.001522
## alternative hypothesis: true difference in means is not equal to 0
## 95 percent confidence interval:
##  -0.21335405 -0.05073728
## sample estimates:
## mean of x mean of y
##  1.122150  1.254196
```

```
plot.hist(x.stable = unsuitable.stable,
          y.unstable = unsuitable.unstable,
          val = "mntd")
```

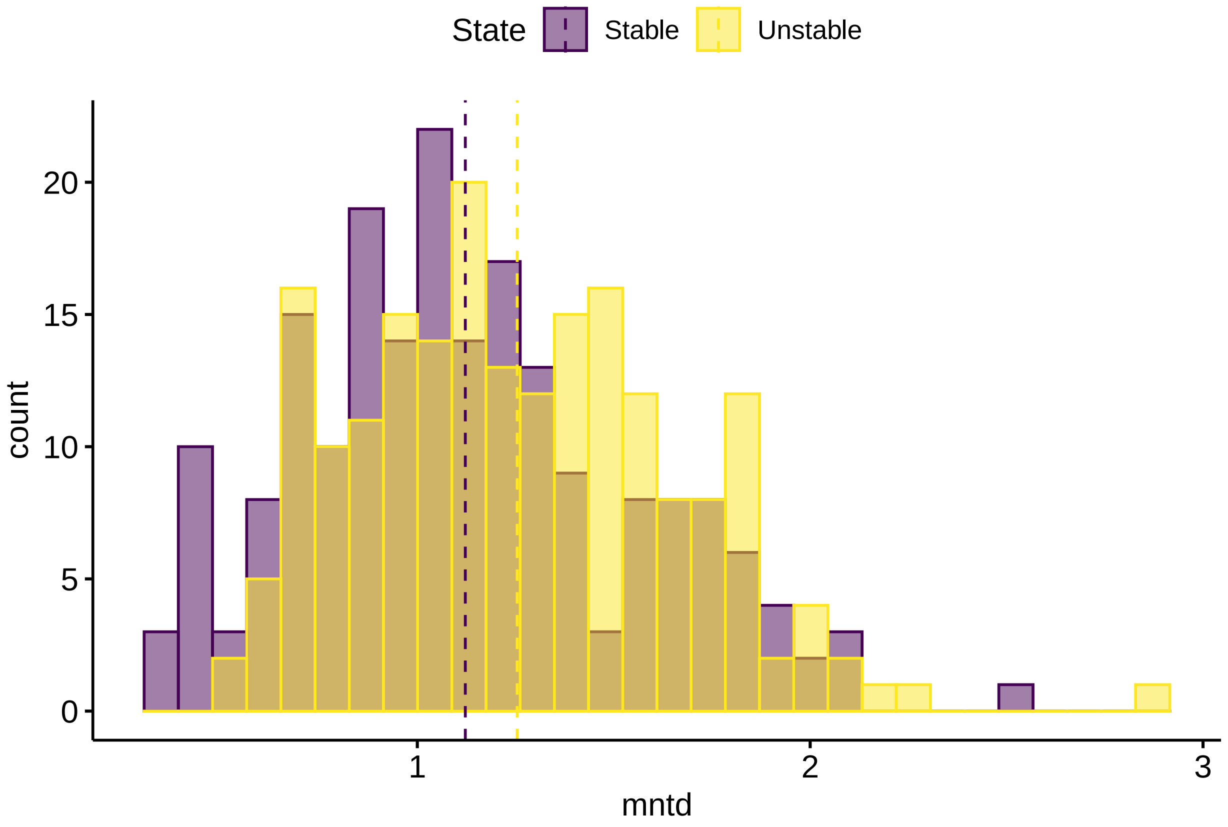

State
Stable
Unstable
count
20
15
10
5
0
1
2
3
mntd
